# Intravital FRAP Imaging using an E-cadherin-GFP Mouse Reveals Disease- and Drug-Dependent Dynamic Regulation of Cell-Cell Junctions in Live Tissue

**DOI:** 10.1016/j.celrep.2015.12.020

**Published:** 2015-12-24

**Authors:** Zahra Erami, David Herrmann, Sean C. Warren, Max Nobis, Ewan J. McGhee, Morghan C. Lucas, Wilfred Leung, Nadine Reischmann, Agata Mrowinska, Juliane P. Schwarz, Shereen Kadir, James R.W. Conway, Claire Vennin, Saadia A. Karim, Andrew D. Campbell, David Gallego-Ortega, Astrid Magenau, Kendelle J. Murphy, Rachel A. Ridgway, Andrew M. Law, Stacey N. Walters, Shane T. Grey, David R. Croucher, Lei Zhang, Herbert Herzog, Edna C. Hardeman, Peter W. Gunning, Christopher J. Ormandy, T.R. Jeffry Evans, Douglas Strathdee, Owen J. Sansom, Jennifer P. Morton, Kurt I. Anderson, Paul Timpson

**Affiliations:** 1 Cancer Research UK Beatson Institute, Switchback Road, Bearsden, Glasgow G61 1BD, UK; 2 The Garvan Institute of Medical Research and The Kinghorn Cancer Centre, Cancer Division, St Vincent’s Clinical School, Faculty of Medicine, University of New South Wales, Sydney, NSW 2010, Australia; 3 Neuromuscular and Regenerative Medicine Unit, School of Medical Sciences, University of New South Wales, Sydney, NSW 2052, Australia; 4 Oncology Research Unit, School of Medical Sciences, University of New South Wales, Sydney, NSW 2052, Australia

**Keywords:** intravital imaging, FRAP, E-cadherin, Src-kinase, pancreatic cancer, invasion and metastasis, cell adhesion and migration, Kras, p53

## Abstract

E-cadherin-mediated cell-cell junctions play a prominent role in maintaining the epithelial architecture. The disruption or deregulation of these adhesions in cancer can lead to the collapse of tumor epithelia that precedes invasion and subsequent metastasis. Here we generated an E-cadherin-GFP mouse that enables intravital photobleaching and quantification of E-cadherin mobility in live tissue without affecting normal biology. We demonstrate the broad applications of this mouse by examining E-cadherin regulation in multiple tissues, including mammary, brain, liver, and kidney tissue, while specifically monitoring E-cadherin mobility during disease progression in the pancreas. We assess E-cadherin stability in native pancreatic tissue upon genetic manipulation involving Kras and p53 or in response to anti-invasive drug treatment and gain insights into the dynamic remodeling of E-cadherin during in situ cancer progression. FRAP in the E-cadherin-GFP mouse, therefore, promises to be a valuable tool to fundamentally expand our understanding of E-cadherin-mediated events in native microenvironments.

## Highlights

• The E-cadherin-GFP mouse allows in situ quantification of E-cadherin mobility
• We monitored E-cadherin mobility during tissue homeostasis and disease development
• Invasive pancreatic cancer driven by mutant Kras/p53 increases E-cadherin mobility
• Dasatinib treatment reverts E-cadherin mobility and reinforces tumor cell junctions

## Graphical Abstract

**Figure:**
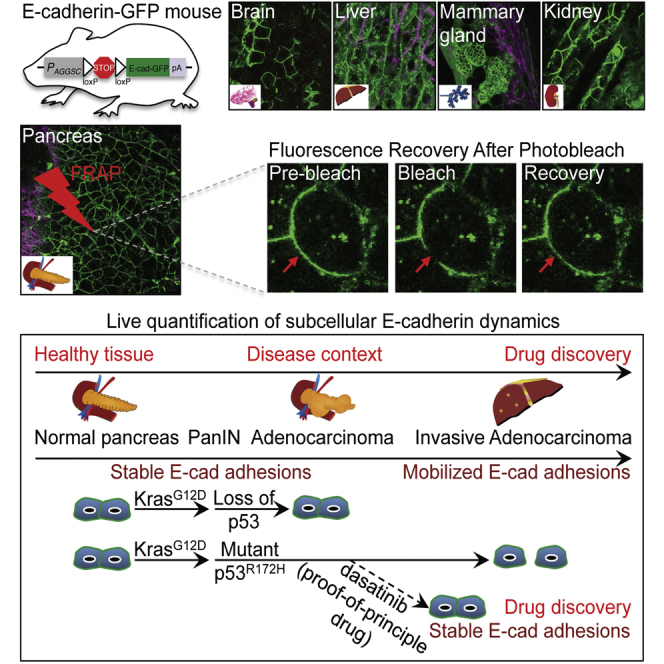

## Teaser

Erami et al. generate an E-cadherin-GFP mouse to demonstrate real-time quantification of E-cadherin mobility using intravital photobleaching in a range of tissue types. They show that changes in E-cadherin mobility correlate with changes in cell junction integrity and invasiveness while demonstrating applications of the mouse for future drug discovery studies.

## Introduction

The capacity of cancer cells to dissociate from primary tumors and invade requires the deregulation of interactions with adjacent cells and the surrounding tissue. A major challenge in biology is the real-time monitoring of protein dynamics involved in this process in their native context (Conway et al., 2014). The ability to quantify the intricate spatiotemporal regulation of cell adhesion molecules, such as E-cadherin, using techniques including fluorescence recovery after photobleaching (FRAP) has rapidly enhanced our understanding of E-cadherin’s subcellular roles in regulating cell-cell integrity and dissociation in vitro (Canel et al., 2010a, Shen et al., 2008, Sprague and McNally, 2005, Wu et al., 2014).

FRAP is commonly used for monitoring molecular movement within cells. A small fluorescent region is bleached, and fluorescence recovery into the bleached region is measured over time (Axelrod et al., 1976, Fritzsche and Charras, 2015, Sprague and McNally, 2005). From this, multiple readouts can be derived, including, but not limited to the half-time of recovery, a measure of the rate at which fluorescent molecules move in or out of the region of interest, and the immobile fraction, an indication of how much of the molecule remains trapped and unable to move out of the analyzed region (for in-depth insights into FRAP analysis, see Fritzsche and Charras, 2015). In the case of fluorescently labeled E-cadherin, the immobile fraction can indicate how much E-cadherin is trapped or engaged in cell-cell junctions and may provide a molecular readout of junction stability in real time (Canel et al., 2010b, Serrels et al., 2009).

FRAP has largely been used to probe molecular events within 2D cell culture using transfection-based approaches (Lippincott-Schwartz et al., 2001, Shen et al., 2008), whereas its utility in vivo has been limited (Ellenbroek and van Rheenen, 2014). Recently, we and others have used FRAP in more complex and physiologically relevant environments ranging from application in *Drosophila* (Cavey et al., 2008) to the use of E-cadherin-GFP FRAP in a mammalian system in vivo (Serrels et al., 2009), where E-cadherin-GFP-expressing squamous cell carcinoma cells were transplanted and grown subcutaneously in mice. Using this approach, we demonstrated that locally invading cells had a significantly lower immobile fraction of E-cadherin-GFP compared with non-invading cells, illustrating that, in cancer cells that retain E-cadherin expression, mobilization rather than loss of E-cadherin can play a role in reducing cell-cell adhesions, leading to more malleable, motile, and invasive tumor behavior (Friedl et al., 2012, Lamouille et al., 2014, Shamir et al., 2014). Although these approaches provide insights into the mobilization of E-cadherin in subcutaneous xenograft tumors, they lack the fidelity to recapitulate the complex and distinct microenvironment found in specific organs of interest (Conway et al., 2014). It is therefore essential to develop new tools for the quantification of molecular dynamics in distinct organs in which the disease of interest originates, allowing us to understand cell behavior at a subcellular and tissue- and disease-specific level.

In this study, we generated a Cre-inducible E-cadherin-GFP mouse and exploited this model using intravital FRAP imaging to assess changes in E-cadherin-based cell-cell junction integrity during disease progression and in response to drug treatment. Here we mimic the disease etiology of pancreatic cancer in situ, from acquisition of an initiating Kras mutation that occurs in 95% of pancreatic cancers (Biankin et al., 2012, Hingorani et al., 2003) to subsequent loss- or gain-of-function mutations in the tumor suppressor p53, which occur in 50%–75% of pancreatic cancers (Biankin et al., 2012). Using FRAP analysis, we reveal that E-cadherin stability in normal pancreas or in non-invasive pancreatic tumors (Kras^G12D^ alone or Kras^G12D^; p53^−/−^) remains unaltered during disease progression. However, in mice with Kras^G12D^ and gain-of-function mutations in p53 (p53^R172H^), E-cadherin is mobilized, facilitating the weakening of cell-cell contacts, correlating with the enhanced metastasis seen in this model (Hingorani et al., 2005, Morton et al., 2010b). Moreover, in line with recent work assessing Src kinase as an anti-invasive drug target (Avizienyte et al., 2002, Morton et al., 2010a, Nobis et al., 2013, Nobis et al., 2014), we demonstrate that the phase II drug dasatinib reverts E-cadherin mobilization in invasive pancreatic tumors and that this stabilization of junctions could partially explain its current anti-invasive role in this disease (T.J. Evans et al., 2012, ASCO, abstract). We therefore present the application of FRAP in the E-cadherin-GFP mouse for live, tissue-specific assessment of vital cell-cell adhesion changes in conjunction with its utility as a pre-clinical imaging tool for evaluating the efficacy of new therapeutics in the pancreas and other organs of interest in real time.

## Results

### Generation of E-cadherin-GFP Mice for In Vivo FRAP Assessment

**Figure 1:**
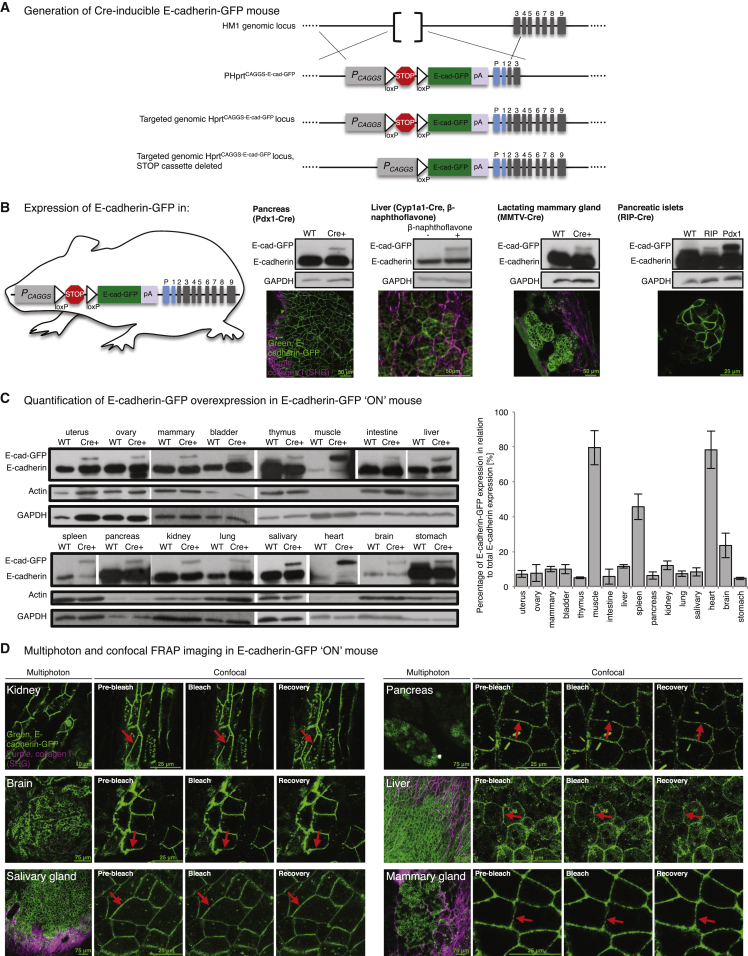
Generation of Conditional and Constitutive E-cadherin-GFP Mice (A) Schematic of the generation of a conditional E-cadherin-GFP OFF strain by targeting a lox-stop-lox transgene under the control of a CAG promoter to the *Hprt* locus. (B) Conditional E-cadherin-GFP expression in pancreas (Pdx1-Cre), liver (Cyp1a1-Cre), mammary gland (MMTV-Cre), and pancreatic islets (RIP-Cre). Shown are western Blot analyses in wild-type (WT) and transgenic organs (top band, E-cadherin-GFP; bottom band, endogenous E-cadherin) and corresponding multiphoton images. (C and D) Western Blot analyses in 16 organs (C) and multiphoton images of E-cadherin GFP expression (D, column 1) and confocal FRAP (D, columns 2–4) in six organs of the E-cadherin-GFP ON mouse. E-cadherin-GFP, green; SHG signal, purple; red arrow, bleach point. See also Figure S1.

Conditional E-cadherin-GFP-expressing mice were generated by targeting a lox-stop-lox transgene under the control of a CAG promoter into the deleted *Hprt* locus of HM1 embryonic stem (ES) cells (Figure 1A; Bronson et al., 1996). We first generated E-cadherin-GFP “OFF” mice, a strain in which expression of the extensively validated EGFP-linked E-cadherin fusion protein (Lock and Stow, 2005) was conditionally prevented by a transcriptional stop sequence (Figure 1A). To demonstrate the utility and scope of the E-cadherin-GFP mouse for examining the regulation of E-cadherin in different organs, we crossed E-cadherin-GFP OFF mice to tissue-specific Cre strains to induce organ- and cell-type-specific expression in the pancreas (the focus of this study), liver, mammary gland, and pancreatic islets (Figure 1B). Expression of E-cadherin-GFP in the pancreas via Pdx1-Cre could be imaged readily at depth (Movie S1, first panel; green, E-cadherin-GFP; purple, second harmonic generation [SHG] imaging of the extracellular matrix [ECM]). In the liver, E-cadherin-GFP OFF mice were crossed to Cyp1a1-Cre mice (Ireland et al., 2004), and deletion of the stop cassette was achieved after three doses of 2 mg β-naphthoflavone over 8 hr (Movie S1, second panel). Robust expression in mammary tissue via MMTV-Cre (Wagner et al., 1997) was imaged in the alveoli of lactating mice where junction integrity is required during milk production (Movie S1, third panel; Figures S1A and S1B), whereas sub-organ specificity within islet cells of the pancreas via RIP-Cre (Postic et al., 1999) could be demonstrated in isolated pancreatic islets (Movie S1, fourth panel).

Conditional E-cadherin-GFP mice were subsequently crossed to mice expressing CMV Cre recombinase (Schwenk et al., 1995) to enable constitutive, low-level expression of E-cadherin-GFP in every tissue (Figure 1C). Analysis of E-cadherin in multiple tissue types from the E-cadherin-GFP “ON” mouse illustrates the broader capacity for subcellular FRAP imaging in normal or disease contexts of interest (Figure 1D; Movie S2, left panels, kidney, brain and salivary gland; right panels, pancreas, liver, and mammary tissue). Importantly, mice of both E-cadherin-GFP strains were born at the expected Mendelian ratio, were fertile and healthy, and exhibited no untoward behaviors. In this study, we demonstrate the application of photobleaching in the E-cadherin-GFP mouse as a method for assessing the subcellular regulation of E-cadherin in the progression of invasive pancreatic cancer. However, this system could be used in other disease states in future investigations.

### FRAP Assessment of E-cadherin in Two versus Three Dimensions Reveals Mobilization Differences between Non-invasive and Invasive Pancreatic Cancer

**Figure 2:**
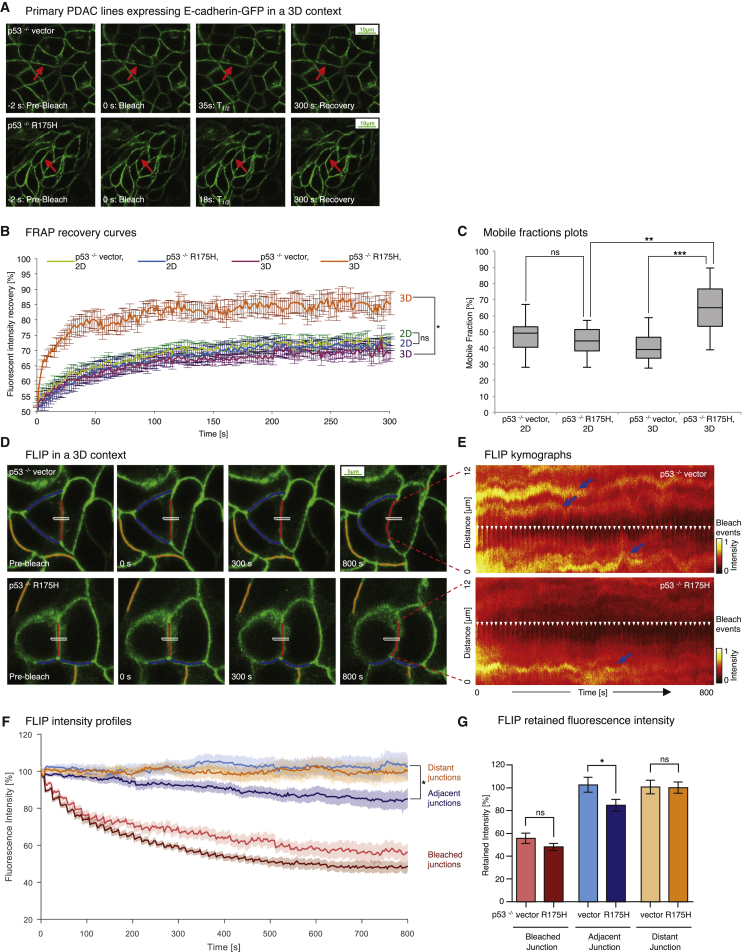
E-cadherin Junctions Are Mobilized in Three Dimensions in Invasive PDAC Cells (A) Representative images from confocal FRAP movies of non-invasive p53^−/−^ vector (top) versus invasive p53^−/−^ R175H cells (bottom) grown on CDM. Arrows, bleached regions. (B and C) FRAP recovery curves (B) and mobile fraction plots (C) comparing E-cadherin-GFP mobilization between p53^−/−^ vector and p53^−/−^ R175H cells grown in a 2D versus 3D environment. n = 3, >10 junctions/group. (D) Representative images from FLIP movies of p53^−/−^ vector (top) and p53^−/−^ R175H cells (bottom) grown on CDM. White rectangles, bleach regions; red lines, tracked bleached junctions; blue lines, tracked adjacent junctions; orange lines, distant control junctions. (E) Photobleach-corrected kymographs showing the intensity profile along the bleached junction over time. White arrows, bleach events. (F and G) Photobleach-corrected, normalized intensity profiles (F) and averaged fraction of fluorescence retained after 800 s (G) for bleached (red), adjacent (blue), and distant junctions (orange) of p53^−/−^ vector (light colors, n = 13) and p53^−/−^ R175H cells (dark colors, n = 14). Columns, mean; bars, ± SE. ^∗^p < 0.05; ^∗∗^p < 0.01; ^∗∗∗^p < 0.001. ns, not significant (unpaired Student’s t test). See also Figures S2 and S3.

Prior to generation of the E-cadherin-GFP mouse, we first established primary non-invasive pancreatic tumor cell lines from Pdx1-Cre; Kras^G12D/+^; p53^flox/+^ mice (Morton et al., 2010b) in which the wild-type p53 allele is lost (hereafter referred to as p53^−/−^). Using these cells, we generated stable pancreatic ductal adenocarcinoma (PDAC) p53^−/−^ cells expressing empty vector alone (p53^−/−^ vector) or the human equivalent of murine p53^R172H^ (p53^−/−^ R175H) with E-cadherin-GFP (Muller et al., 2013, Tan et al., 2014). The resulting invasive mutant p53^−/−^ R175H and non-invasive p53^−/−^ vector cells expressing E-cadherin-GFP (Figure 2A; Movie S3) serve as a fluorescent model system to gauge the initial spatiotemporal regulation of E-cadherin in pancreatic cancer (Morton et al., 2010b, Muller et al., 2013, Tan et al., 2014) without affecting fundamental processes such as cell proliferation or invasive capacity (Figures S1C and S1D).

FRAP recovery curves from time-lapse movies of p53^−/−^ vector or p53^−/−^ R175H primary PDAC cells were then analyzed (Canel et al., 2010a, Canel et al., 2010b, Serrels et al., 2009, Vlahov et al., 2015; Supplemental Experimental Procedures; Figure S2). Assessment in two dimensions revealed no difference in E-cadherin mobility of invasive p53^−/−^ R175H versus non-invasive p53^−/−^ vector cells (Figure 2B, blue recovery curve versus green), where the fraction of E-cadherin-GFP stabilized in cell-cell junctions was similar in both cell types (Figure 2C, mobile fraction 45.6% ± 2.7% for p53^−/−^ vector compared with 46% ± 3.1% for p53^−/−^ R175H cells).

Critically, assessment in a 3D environment using cells grown on cell-derived matrices (CDMs) to mimic in vivo conditions (Herrmann et al., 2014, Yamada and Cukierman, 2007) revealed enhanced mobilization of E-cadherin in invasive cells, where mutant p53^−/−^ R175H cells had a significantly higher mobile fraction of E-cadherin compared with p53^−/−^ vector cells (Figures 2B, orange recovery curve versus purple, and 2C, average mobile fraction 39.9% ± 2.8% versus 65.6% ± 4.4%; Figure S3A). This is in line with the early cell-cell dissociation preceding cell scattering and invasion we observed recently in mutant p53 cells compared with p53^−/−^ cells (Muller et al., 2013, Tan et al., 2014) and highlights the need to assess E-cadherin cell-cell adhesion events in a 3D context.

To further assess E-cadherin mobility and corroborate our findings in this 3D setting, complementary fluorescence loss in photobleaching (FLIP) analysis was performed on p53^−/−^ vector versus mutant p53^−/−^ R175H cells grown on CDMs (Figures 2D–2G; Sakurai-Yageta et al., 2015). Here, by continually bleaching an area within a cell-cell junction, we observed that, in p53^−/−^ vector cells, junctions adjacent to the bleached region did not significantly reduce fluorescence intensity over the time course of our FLIP analysis after correcting for imaging-induced photobleaching (Figures 2D and 2E; Movie S4 [the white rectangle indicates a repeated bleach region, and red, blue, and orange lines represent bleached, adjacent, and distant non-related control junctions, respectively]). Here, FLIP traces of adjacent junctions (Figures 2F and 2G, light blue line and bar graph) show no significant reduction in intensity over the 800-s time course. In contrast, mutant p53^−/−^ R175H cells exhibited a significant loss of fluorescence intensity in junctions adjacent to the bleach region over time (Figures 2F and 2G, dark blue line and bar graph), commensurate with the transfer of bleached E-cadherin molecules into this area and an enhanced mobilization of E-cadherin junctions in mutant p53 cells compared with p53^−/−^ vector cells.

To assess whether E-cadherin is distributed equally at distinct points of the membrane in PDAC cells, we created kymographs of unbleached junctions from p53^−/−^ vector and p53^−/−^ R175H cells (Figures S3B–S3D). We observed an elevated distribution of high-intensity zones of E-cadherin-GFP in p53^−/−^ vector cells compared with p53^−/−^ R175H cells (Figures S3C and S3D, blue arrows). These zones persisted over the time course of imaging and could represent stable E-cadherin zones, as identified previously by others (Adams et al., 1998, Cavey et al., 2008). To quantify the uniformity or texture of this E-cadherin intensity across the membrane, we used orientation-dependent grey level co-occurrence matrix (OD-GLCM) analysis of the kymographs (Figure S3B; Hu et al., 2012, Nobis et al., 2013). This analysis revealed that the inherent distribution of high-intensity E-cadherin-GFP zones was significantly different between p53^−/−^ vector and p53^−/−^ R175H junctions (quantified as contrast in Figure S3E), indicating a larger number of zones of high E-cadherin intensity in non-invasive p53^−/−^ vector cells. Collectively, these data support our initial FRAP results, revealing a critical mobilization of E-cadherin in mutant p53 cells in 3D in vitro settings.

**Figure 3:**
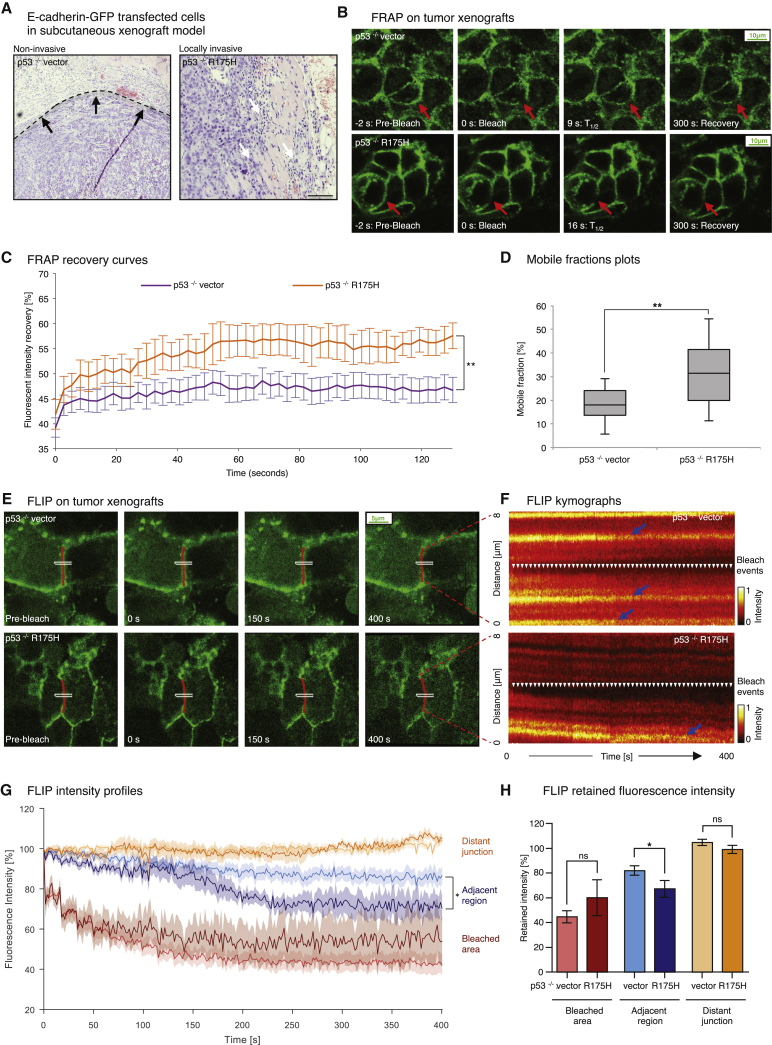
FRAP and FLIP in Live Tumors Reveal E-cadherin Mobilization during Invasion (A) Representative images of encapsulated p53^−/−^ vector tumors (left, dashed line, black arrows) versus locally invasive p53^−/−^ R175H tumors (right, white arrows). (B) Representative images from confocal FRAP movies of p53^−/−^ vector (top) versus p53^−/−^ R175H xenografts (bottom). Red arrows, bleached regions. (C and D) FRAP recovery curves (C) and mobile fraction plots (D) comparing E-cadherin-GFP mobilization between p53^−/−^ vector and p53^−/−^ R175H cells grown in a 2D versus 3D environment. n = 3, >10 junctions/group. (E) Representative images from FLIP movies of p53^−/−^ vector (top) and p53^−/−^ R175H xenografts (bottom). White rectangles, bleach regions; red lines, tracked bleached junctions. (F) Photobleach-corrected kymographs showing the intensity profile along the bleached junction over time. Arrows, bleach events. (G and H) Photobleach-corrected, normalized intensity profiles (G) and averaged fraction of fluorescence retained after 400 s (H) for the bleached region (red), areas 3 μm distant from the bleached region (blue), and distant control junctions (orange) of p53^−/−^ vector (light colors, n = 9) and p53^−/−^ R175H cells (dark colors, n = 5). Columns, mean; bars, ± SE; n = 3 mice; >5 cells/group. ^∗^p < 0.05; ^∗∗^p < 0.01 (unpaired Student’s t test). See also Figures S2 and S3.

PDAC cells were therefore injected into CD1 nude mice and allowed to form subcutaneous tumors. In line with previous work (Muller et al., 2013, Timpson et al., 2011), p53^−/−^ R175H tumor-bearing mice demonstrated collective local invasion compared with encapsulated non-invasive p53^−/−^ vector tumors (Figure 3A). FRAP recovery curves from time-lapse movies of xenograft tumors obtained using intravital FRAP imaging (Figure 3B) revealed an enhanced mobile fraction in p53^−/−^ R175H versus p53^−/−^ vector cells in this setting (Figures 3C and 3D, 32.1% ± 2.9% versus 18.8% ± 1.7%; Figure S3F; Movie S5). Similarly, FLIP analysis of the xenograft tumors (Figures 3E–3H; Movie S6) shows a significantly higher loss of fluorescence in areas proximal to the bleach region in p53^−/−^ R175H tumors compared with p53^−/−^ vector tumors, corroborating our FRAP analysis (Figures 3G and 3H, compare light blue and dark blue FLIP traces and bar graphs). Furthermore, OD-GLCM analysis of unbleached junctions again showed a higher contrast of high versus low E-cadherin-GFP intensity/texture in p53^−/−^ vector tumors compared with p53^−/−^ R175H tumors (Figures S3G–S3I), indicating an increased stabilization of E-cadherin in p53^−/−^ vector control cells in vivo.

**Figure 4:**
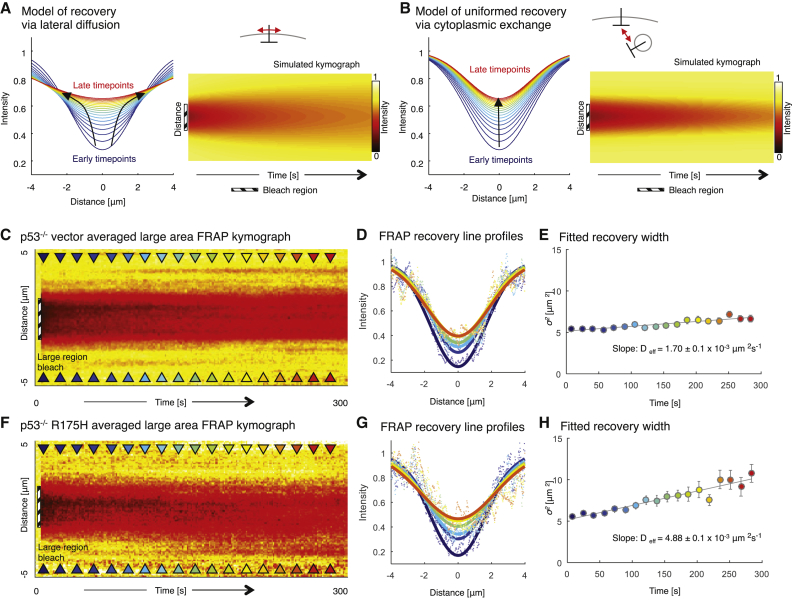
Large-Area E-cadherin-GFP FRAP in PDAC Xenografts (A, B, C, and F) Models of recovery via lateral membrane diffusion (A) and via cytoplasmic exchange (B) with line profiles along the bleached region during recovery (early time points, blue; late time points, red) and simulated intensity kymographs. Black arrows highlight changes in recovery (C and F). Also shown is a normalized average kymograph for large-region FRAP of p53^−/−^ vector (C, n = 9) and p53^−/−^ R175H (F, n = 9) xenografts. Colored arrowheads indicate time points where profiles were fitted and shown in later plots (early time points, blue; late time points, red). Dashed black areas, bleached regions. (D and G) Line profiles from recovery (early time points, blue; late time points, red) with fitted Gaussian curves (solid lines). (E and H) Fitted width (σ^2^) of Gaussian curves for selected time points, with linear fit (gray line) used to calculate the diffusion coefficient (slope). Error bars, confidence interval on fitted parameter.

To further investigate the differences in E-cadherin mobility between p53^−/−^ vector and p53^−/−^ R175H tumors, we bleached a large region of interest with a 3-μm diameter. The change in intensity profile along the membrane of a large FRAP bleach region over time can provide insights into the processes governing recovery (modeled in Figures 4A and 4B). We therefore generated kymographs of the bleached junction and measured the fluorescence intensity along the junction via line scan analysis at defined time points (Figures 4C and 4F, colored arrowheads; de Beco et al., 2012, Delva and Kowalczyk, 2009). We then fitted the results of our line scan analysis to a Gaussian curve model (Figures 4D and 4G) and plotted the decrease in width of the Gaussian curves over time as a measure of the speed of fluorescence recovery (Figures 4E and 4H). If the recovery was due to lateral movement in the membrane, then the width of the bleached region should increase linearly with time (Figure 4A). Conversely, if the recovery was due to cytoplasmic uniformed exchange, then the width of the bleached region should remain uniform with time (Figure 4B). Here we find that the width of the bleached region expands significantly faster in p53^−/−^ R175H xenografts compared with p53^−/−^ vector xenografts, indicating faster lateral motion of E-cadherin in the mutant xenografts (Figures 4C–4H; compare the slopes as a measure of the recovery speed between Figures 4E and 4H, with the effective diffusion coefficient *D*_*eff*_ = 4.88 ± 0.3 × 10^−3^ μm^2^ s^−1^ versus 1.70 ± 0.1 × 10^−3^ μm^2^ s^−1^, respectively). This indicates that the increase in E-cadherin mobility observed in locally invasive p53^−/−^ R175H xenografts may be primarily attributed to increased mobility within the membrane.

Although this subcutaneous model confirmed that enhanced mobilization of E-cadherin exists in live 3D tumor settings, it does not recapitulate the distinct microenvironment found in the pancreas. This prompted us to investigate whether subcellular mobilization of E-cadherin occurs in the organ of tumor origin and, if so, whether the stage of in situ disease progression in which these changes occur can be identified (Bardeesy and DePinho, 2002).

### FRAP in the E-cadherin-GFP Mouse Reveals Genetically Driven Progression of Cell-Cell Junction Disruption in Pancreatic Tissue during In Situ Disease Development

**Figure 5:**
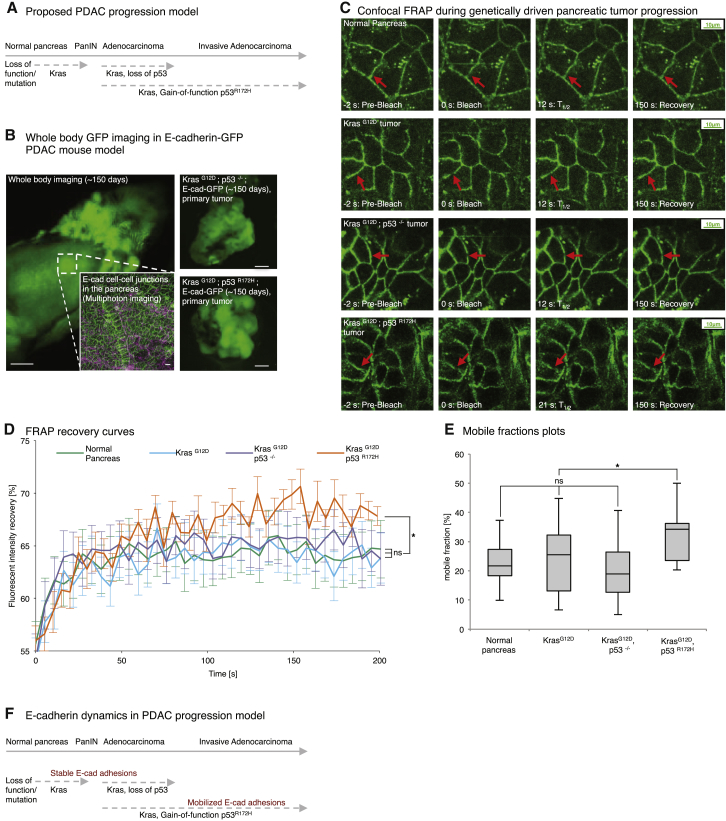
E-cadherin-GFP FRAP during Tissue Homeostasis, Initiation, and Progression of Pancreatic Cancer (A) Proposed pancreatic cancer progression model from initiating Kras mutations (PanIN) to subsequent loss of p53 or gain-of-function mutations in p53. (B) Representative whole-body image of an E-cadherin-GFP mouse with multiphoton-based cell-cell junction imaging (inset: E-cadherin-GFP, green; SHG signal, purple). (C) Representative images from confocal FRAP movies in the E-cadherin-GFP mouse from normal pancreas (first row) to acquiring Kras^G12D^ (second row) with subsequent loss of p53 (third row) or p53^R172H^ gain-of function mutations (bottom row), respectively. Red arrows, bleached regions. (D and E) Graphs comparing E-cadherin-GFP mobilization between in situ pancreatic tissue from normal (green), Kras^G12D^ alone (blue), Kras^G12D^; p53^−/−^ (purple), and Kras^G12D^; p53^R172H^ (orange) mice. Columns, mean; bars, ± SE, n = 3 mice/group, ≥21 junctions in total. ^∗^p < 0.05 (unpaired Student’s t test). (F) Proposed schematic of E-cadherin dynamics during PDAC progression. See also Figure S4.

Our ability to use FRAP in vivo led us to investigate whether we can assess E-cadherin mobility in live pancreatic tissue. The E-cadherin-GFP mouse was therefore crossed with either wild-type mice expressing Pdx1-Cre, mice bearing a Pdx1-Cre-driven initiating Kras^G12D^ mutation alone, Pdx1-Cre; Kras^G12D^; p53^−/−^ mice, or Pdx1-Cre; Kras^G12D^; mutant p53^R172H^ mice and monitored for ∼150 days to allow tumor formation to occur. This allowed us to recapitulate the genetic changes commonly occurring during the formation of invasive PDAC (Figures 5A–5C; Movie S7) in this extensively validated model of pancreatic cancer.

FRAP recovery curves from time-lapse movies of wild-type E-cadherin-GFP mice (Figure 5D, green line) were analyzed to gauge the inherent mobility of E-cadherin in normal pancreatic tissue (Figure 5E, average mobile fraction = 21.9% ± 1.6%; Figure S4A). Interestingly, the fraction of E-cadherin stabilized in cell-cell junctions in wild-type mice was similar to that of non-invasive tumors bearing Kras^G12D^ (Figures 5D and 5E, blue line, mobile fraction = 24.3% ± 2.3%). This indicates that initiating Kras^G12D^ mutations in the pancreas are not sufficient to drive E-cadherin mobilization in situ.

Importantly, loss of p53 on a Kras^G12D^ background from age-matched tumors also had no effect on E-cadherin mobility compared with normal pancreatic tissue or Kras^G12D^ tumors alone (Figures 5D and 5E, purple line, 21.6% ± 1.7% mobile fraction). This demonstrates that, although loss of the tumor suppressor p53 in combination with initiating Kras^G12D^ mutations enhances tumor progression, it does not act to mobilize E-cadherin, correlating with the lack of invasion or metastasis we observed previously in Kras^G12D^; p53^−/−^ mice (Morton et al., 2010b).

Critically, FRAP analysis of tumors from ∼150-day-old, Kras^G12D^; p53^R172H^; E-cadherin-GFP mice revealed that gain-of-function mutations in p53 on a Kras^G12D^ background significantly mobilizes E-cadherin junctions in the pancreas (Figures 5D and 5E, orange line, 31.8% ± 1.8% mobile fraction compared with the average of 22.4% ± 1.1% found in normal pancreas, Kras^G12D^, or Kras^G12D^; p53^−/−^ tumors). This indicates that acquisition of mutant p53^R172H^, which we have shown previously to drive an invasive and metastatic program over and above the loss of p53 in pancreatic cancer (Morton et al., 2010b), could partially achieve this by allowing early tumor dissolution via mobilization of E-cadherin-based cell-cell contacts (Figure 5F). Therefore, we sought to assess whether this potentially pro-invasive molecular event could be impaired pharmacologically and monitored in a pre-clinical setting using the E-cadherin-GFP mouse.

### The E-cadherin-GFP Mouse as a Tool to Monitor Early Anti-invasive Drug Response in Real Time

Recently we demonstrated that Src kinase, which is known to induce the disruption of E-cadherin-based cell-cell junctions either directly (Avizienyte et al., 2002, Calautti et al., 1998, Frame et al., 2002) or indirectly via the stroma (Yang et al., 2010), plays a prominent role in invasive pancreatic cancer (Morton et al., 2010a). We therefore examined whether the mobilization and potential weakening of E-cadherin junctions observed in invasive pancreatic tumors of Pdx1-Cre; Kras^G12D^; p53^R172H^ E-cadherin-GFP mice could be reverted using dasatinib.

**Figure 6:**
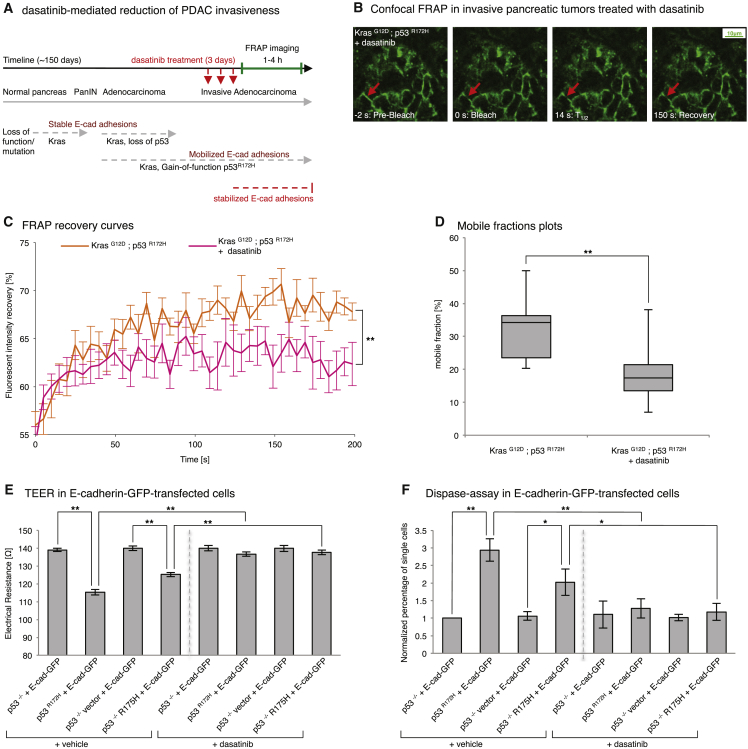
The E-cadherin-GFP Mouse as a Tool to Monitor the Early Anti-invasive Drug Response (A) Schematic of anti-invasive dasatinib treatment using Kras^G12D^; p53^R172H^; E-cadherin-GFP mice. (B) Representative images from confocal FRAP movies of dasatinib-treated Kras^G12D^; p53^R172H^; E-cadherin-GFP mouse (oral gavage, 3× daily, 10 mg/kg). Red arrows, bleached regions. (C and D) Graphs comparing E-cadherin-GFP mobilization in in situ pancreatic tissue of control (orange) and dasatinib-treated (pink) Kras^G12D^; p53^R172H^; E-cadherin-GFP mice. Columns, mean; bars, ± SE; n = 3 mice/group, ≥21 junctions in total. ^∗∗^p < 0.01 (unpaired Student’s t test). (E and F) TEER (E) and Dispase assays (F) in E-cadherin-GFP-transfected p53^−/−^ PDAC cells (columns 1 and 3, pre-dasatinib; columns 5 and 7, post-dasatinib) versus mutant p53 PDAC cells (columns 2 and 4, pre-dasatinib; columns 6 and 8, post-dasatinib). Columns, mean; bars, ± SE; n = 3 mice/group. ^∗^p < 0.05; ^∗∗^p < 0.01 (unpaired Student’s t test). See also Figure S4.

As before, Pdx1-Cre; Kras^G12D^; p53^R172H^; E-cadherin-GFP mice were allowed to form tumors for ∼150 days and, upon signs of tumor burden, were placed on daily dasatinib treatment (10 mg/kg) for 3 consecutive days (Figure 6A), followed by FRAP analysis (Figure 6B; Movie S7). This revealed that reversion of E-cadherin mobilization back to the levels found in wild-type pancreas or non-invasive Kras^G12D^; p53^−/−^ tumors could be achieved using dasatinib treatment at this stage of the disease (Figures 6C and 6D, compare mobile fraction of Kras^G12D^; p53^R172H^ mice ± dasatinib, 18.1% ± 1.3% versus 31.8% ± 1.8%, and 5E, Kras^G12D^ alone or Kras^G12D^; p53^−/−^, mobile fraction of 24.3% ± 2.3% and 21.6% ± 1.7%; Figure S4B). These results indicate that the recently demonstrated efficacy of dasatinib in this model could partly be due to its capacity to stabilize E-cadherin junctions and prevent dissociation from the primary tumor. We therefore sought to determine whether the E-cadherin-GFP FRAP readouts we observed in this study could be correlated with changes in junction integrity and strength.

Using complementary assays, we interrogated the integrity and strength of cell-cell junctions using trans-endothelial electrical resistance (TEER) and Dispase II assays, respectively (Calautti et al., 1998, Canel et al., 2010b, von Bonsdorff et al., 1985). TEER allows a rapid evaluation of junction integrity as a function of electrical resistance between cells where high resistance correlates with high junction integrity (von Bonsdorff et al., 1985). Examination of the electrical resistance between non-invasive p53^−/−^ and invasive p53^R172H^ cells transfected with E-cadherin-GFP demonstrated that junction integrity was significantly lower in a mutant p53^R172H^ background than in a p53^−/−^ setting (Figure 6E, columns 1 and 2). Similar results were obtained in E-cadherin-GFP-transfected p53^−/−^ R175H and p53^−/−^ vector cells (Figure 6E, columns 3 and 4), confirming that mutant p53 reduces junction integrity in PDAC cells.

In line with our results demonstrating that dasatinib impairs invasion via potentially enhancing cell-cell adhesions, TEER analysis of dasatinib-treated mutant p53-expressing PDAC cells (E-cadherin-GFP-transfected p53^R172H^ or p53^−/−^ R175H cells) revealed that Src inhibition (Figures S4D and S4E) stabilized and strengthened junctions to a similar level as that found in a non-invasive p53^−/−^ setting (Figure 6E, columns 5–8). This was confirmed using the Dispase II assay, where mutant p53 cells (E-cadherin-GFP-transfected p53^R172H^ or p53^−/−^ R175H cells) show an increased number of single cells upon mechanical disruption compared with p53^−/−^ E-cadherin-GFP-transfected counterparts (Figure 6F, columns 1–4). Importantly, cell dissociation was reverted to similar levels as those found in non-invasive p53^−/−^ or vector-alone counterparts when mutant p53 cells were treated with dasatinib (Figure 6F, compare columns 1–4 with columns 5–8). These results illustrate that mutant p53 on an initiating Kras^G12D^ background weakens cell-cell junction strength, which could lead to tumor dissociation, and that Src inhibition can partially impair this breakdown. Importantly, we corroborated these finding with another Src inhibitor, saracatenib (Figures S4D and S4E; Nam et al., 2013), and observed a similar strengthening and enhancement of junction integrity via TEER and Dispase II analysis (Figures S4F and S4G), with a corresponding stabilization of E-cadherin mobility, as assessed by FRAP on CDMs (Figure S4C). These data, therefore, demonstrate that imaging subtle changes in the mobility of proteins involved in maintaining normal epithelial architecture, such as E-cadherin, can provide a surrogate marker of dissociation events that may precede the collapse of cell-cell adhesions, correlating with tumor cell invasion.

### Monitoring E-cadherin Dynamics in Secondary Metastases

**Figure 7:**
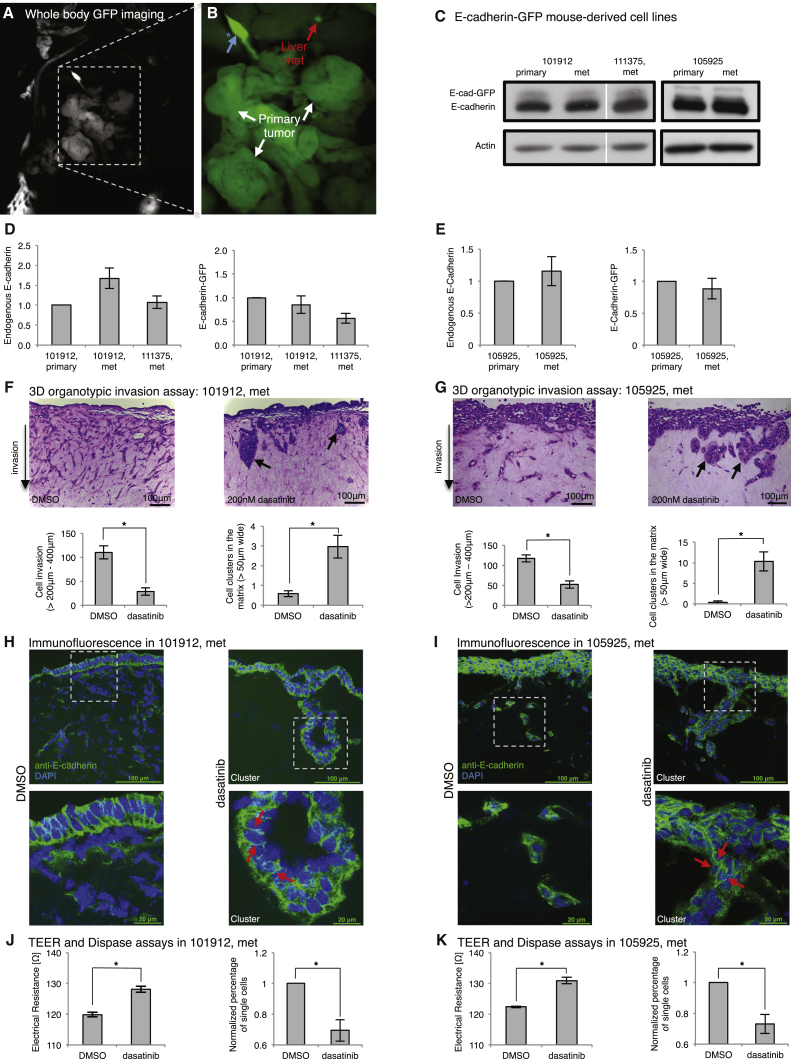
Monitoring E-cadherin Dynamics in Secondary Metastases from the E-cadherin-GFP Mouse (A and B) Whole-body image of primary tumor (A) (B, white arrows) and isolated liver micro-metastases of a Kras^G12D/+^; p53^R172H^; E-cadherin-GFP mouse (B, red arrow). Blue arrow, auto-fluorescence in the bile duct. (C–E) Western blot (C, top band, E-cadherin-GFP; bottom band, endogenous E-cadherin) and quantification (D and E) in isolated the primary and metastatic cell lines 101912 and 111375 (D) and 105925 (E). (F and G) The isolated metastatic cell lines 101912 met (F) and 105925 met (G) from the liver of the E-cadherin-GFP mouse invading in organotypic matrices ± dasatinib (13 days, 200 nM). Black arrows, cluster formation upon dasatinib treatment. Shown is the quantification of cell invasion between 200–400 μm and cell clustering within matrices ± dasatinib. (H and I) Immunofluorescence staining of E-cadherin within matrices ± dasatinib for the metastatic lines 101912 met (H) and 105925 met (I). Red arrows, junctions within cell clusters upon dasatinib treatment. E-cadherin, green; DAPI, blue. (J and K) TEER (left) and Dispase assays (right) in metastatic lines derived from transgenic mouse, 101912 met (J), and 105925 met (K) ± dasatinib. Columns, mean; bars, ± SE; n = 4 mice/group. ^∗^p < 0.05 (unpaired Student’s t test). See also Figures S5 and S6.

In line with the concept of stabilizing E-cadherin junctions via Src inhibition, we isolated metastatic tumor cells that had colonized the liver of invasive Pdx1-Cre; Kras^G12D^; p53^R172H^; E-cadherin-GFP mice using whole-body fluorescence imaging (Figures 7A and 7B, whole-body imaging and inset, respectively, showing primary tumor and liver micro-metastases isolated from the same mouse). We confirmed that metastatic cells from the E-cadherin-GFP mouse retained similar E-cadherin expression levels as cell lines obtained from primary tumors, and similar levels of proliferation (Figures 7C–7E, Figures S5A and S5B), while maintaining the capacity to invade in 3D organotypic matrices (Figures 7F, 7G, and S5C, left). Importantly, upon treatment of these invasive cells with dasatinib, invasion was reduced significantly compared with control cells (Figures 7F, 7G, and S5C, right, for three independent metastatic lines from three independent mice [101912 met, 101025 met, and 111375 met, respectively]). Moreover, the integrity of cell-cell contacts in all lines was partially restored under dasatinib-treated conditions, leading to enhanced cell-cell clustering (Figures 7F, 7G, and S5C). Subcellular immunofluorescent staining of E-cadherin showed improved cell clustering in cells treated with dasatinib (Figures 7H, 7I, and S5D, red arrows). Importantly, we observed a similar reduction in invasion and a restoration in junction integrity for all three metastatic cell lines treated with saracatenib in organotypic assays (Figure S6A–S6E). Collectively, these data indicate that Src inhibition may partially stabilize E-cadherin-based cell-cell junctions and could retard early pancreatic tumor cell dissociation and, subsequently, decrease invasive efficiency by inducing tumor cell clustering.

This led us to examine whether the enhanced mobilization of E-cadherin and weakening of junction strength observed in invasive mutant p53 pancreatic tumors (Figures 3 and 5) could also be partly reverted in these more aggressive metastatic lines from the E-cadherin-GFP mouse. As before, TEER and Dispase II assays were carried out, and, in all three metastatic lines, a significant enhancement in junction integrity and strength was observed following dasatinib treatment (Figures 7J, 7K, and S5E). Again, similar effects on TEER and Dispase II analysis were observed upon saracatenib treatment in all three metastatic cell lines (Figures S6F–S6H). In contrast, dasatinib and saracatenib treatment had no effect on junction integrity and strength in primary tumor cells isolated from Pdx1-Cre; Kras^G12D^; p53^−/−^; E-cadherin-GFP mice (Figures S5F and S6I), which inherently possess high junction strength and integrity. Lastly, in line with our CDM and xenograft data (Figures 2 and 3), OD-GLCM analysis of mutant metastatic lines also revealed a reduction in contrast and a more uniform, malleable organization of E-cadherin-GFP distribution compared with primary tumor cells isolated from Pdx1-Cre; Kras^G12D^; p53^−/−^; E-cadherin-GFP mice (Figures S6J and S6K), suggesting that E-cadherin destabilization is maintained in mutant p53 metastatic cells derived from the E-cadherin-GFP mouse. Collectively, these results highlight the utility of the E-cadherin-GFP mouse as a pre-clinical imaging tool for monitoring early subcellular molecular changes that correlate with more aggressive tumor behavior.

## Discussion

Downregulation or loss of E-cadherin expression occurs in a number of cancer types and is thought to play a key role in epithelial-to-mesenchymal transition (EMT) associated with invasive cancer phenotypes (Gregory et al., 2008, Lamouille et al., 2014). However, in many invasive cancers, including pancreatic cancer, E-cadherin expression is often retained (David and Rajasekaran, 2012, Shamir et al., 2014) and utilized positively during cancer progression (Campbell and Casanova, 2015, Wicki et al., 2006). This indicates that, in many cases, deregulation of E-cadherin mobility rather than expression level may play an additional role in promoting invasion and suggests that monitoring E-cadherin behavior in this context could lead to a greater understanding of cancer invasion and response to new therapeutics (Wicki et al., 2006). Here, we present the generation of an E-cadherin-GFP mouse in which E-cadherin behavior can be monitored within intact tissues, such as the pancreas, liver, brain, kidney, and mammary tissue, via FRAP. Using this mouse in the context of pancreatic cancer, we reveal significant biological insights into the in situ regulation of E-cadherin during normal tissue homeostasis, disease initiation, and progression of pancreatic cancer while highlighting its potential utility for monitoring drug target activity in vivo using FRAP.

We chose the *Hprt* locus to drive E-Cadherin-GFP expression to provide the best signal-to-noise ratio for intravital imaging while aiming to minimize disturbance to early developmental events, such as tissue sorting (Halbleib and Nelson, 2006), and normal tissue function. Our assessment of the E-cadherin-GFP mouse, in the context of the comprehensively characterized *Kras*^G12D/+^*; Trp53*^R172H/+^*; Pdx1-Cre* (KPC) mouse model of pancreatic cancer, suggests that we gauged expression levels well, providing enough fluorescence for detection of pre- and post-photobleaching events without affecting primary tumor growth and metastasis. This is in line with our recent development of the Rac-Förster resonance energy transfer (FRET) biosensor mouse in which low-level expression driven via the *ROSA26* locus provided a sufficient signal for in vivo FRET imaging during disease progression without affecting the biology of the cells (Johnsson et al., 2014).

Crossing the E-cadherin-GFP mouse with distinct genetic models of pancreatic cancer has demonstrated its utility for examining the effects of specific mutations on E-cadherin in a time- and tissue-specific and subcellular manner. In pancreatic cancer, initiating mutations in the *KRAS* gene occur in approximately 95% of patients and are often followed by sequential accumulation of genetic alterations, such as loss of expression, function, or mutations in tumor suppressors and regulators, such as *TP53*, *CDKN2A*, *INK4A/ARF*, and/or *PTEN* (Bardeesy and DePinho, 2002, Kennedy et al., 2011, Ling et al., 2012, Morran et al., 2014). Although we know the frequency of such mutations in PDAC (Biankin et al., 2012), their specific function during development of invasive pancreatic cancer remains unclear. Here, we show that acquiring Kras^G12D^ mutations or subsequent loss of p53 on a Kras^G12D^ background in the pancreas is not sufficient to drive alterations to E-cadherin mobility, whereas acquisition of a gain-of-function mutation in p53 on the same initiating Kras^G12D^ background mobilizes E-cadherin, consistent with the tumor dissolution and metastasis found in these mice.

Importantly, reduction or reversion of cell-cell junction strength was achieved by molecular manipulation in the form of overexpressing the human equivalent of murine p53^R172H^ (R175H) or inhibiting E-cadherin mobilization via Src inactivation, respectively. This demonstrates that mutant p53 can partially drive the breakdown of tumors by disrupting E-cadherin cell-cell engagement via Src. It should be noted that both dasatinib and saracatenib, while inhibiting Src kinase, also target Abl and other kinases (Haubeiss et al., 2010) and were used here in a proof-of-principle context to demonstrate the utility of the mouse in pre-clinical drug discovery applications. To this end, the E-cadherin-GFP mouse could also be used to decipher other molecular pathways downstream of mutant p53 to be targeted in future studies or be used with other Kras or p53 mutations to determine whether E-cadherin mobilization is a general phenomenon for all mutant p53 variants in this disease (Muller et al., 2009, Muller et al., 2013, Muller and Vousden, 2014).

It is important to note that, in this study, we examined E-cadherin-GFP mobility throughout the entire pancreatic tumor tissue. However, heterogeneity is a hallmark of in vivo cancer, and it will be possible, in the future, to map areas of weakened cell-cell adhesions or drug response in the KPC model by examining E-cadherin mobilization in the center versus the invasive front of the tumor (Serrels et al., 2009). Similarly, the drug response in cancer cells in relation to local blood or drug supply could be assessed, as we and others have described previously (Manning et al., 2013, Nobis et al., 2013). In this way, we could determine, in the future, whether spatially regulated environmental cues in the native pancreas drive this heterogeneity or reflect a gradient in anti-invasive drug response in vivo.

Finally, the intricate control of in vivo distribution, function, as well as potential redundancy of EMT regulators such as Snail, Slug, Zeb, or Twist (Brabletz et al., 2013) could be investigated in pancreatic or other cancer types using the E-cadherin-GFP mouse. This could help dissect, in real time, the complex changes in E-cadherin mobility that occur during EMT, which, in many cases, does not involve loss of E-cadherin expression but rather a more complex dynamic regulation and utilization of E-cadherin function (David and Rajasekaran, 2012, Shamir et al., 2014, Wu et al., 2015).

The location, signaling, and dynamics of E-cadherin in the context of the surrounding environment can play a vital role in disease progression. Our capacity to isolate and image micro-metastases in the liver illustrates the power of this model for assessing subcellular changes in E-cadherin dynamics at secondary tumor sites that retain E-cadherin expression and may take advantage of this existing pool of E-cadherin during the early stages of mesenchymal-to-epithelial transition (MET) and tumor colonization (Lamouille et al., 2014, Ocaña et al., 2012). EMT-to-MET switching during extravasation and colonization is thought to be controlled precisely and has significant implications on whether anti-invasive drug targeting will be effective in impairing subsequent tumor colonization of the primary tumor in secondary sites (Brabletz et al., 2013). Preventing niche localization at the early stage of colonization by using anti-migratory drug targeting has been shown recently to be an effective strategy in impairing metastasis formation in the liver (Ritsma et al., 2012). However, understanding when and where EMT versus MET are detrimental or conducive to tumor extravasation will be of great interest in the future (Brabletz et al., 2013). Therefore, FRAP in the E-cadherin-GFP mouse could help us to understand this largely unexplored area of the metastatic cascade in a spatiotemporal and pre-clinical manner by allowing us to follow E-cadherin dynamics in both primary and secondary sites in the same mouse.

We focus on the application of FRAP in this study. However, other imaging techniques such as FLIP or anisotropy imaging of homo-FRET could also be employed in the future using the E-cadherin-GFP mouse to address more fundamental questions regarding E-cadherin regulation at distinct points in the membrane in situ (Levitt et al., 2015, Roberti et al., 2011). Furthermore, for fine-tuned assessment of E-cadherin via the endogenous E-cadherin promoter, engineering of a knockin E-cadherin-GFP mouse is currently underway as a next-generation mouse. Therefore, subtle changes to both E-cadherin levels and mobility could be monitored simultaneously during disease progression in cancers that also modulate E-cadherin expression. This could avoid any potential caveats or off-target effects caused by wrong lineage expression and could circumvent any minor changes to physiology that may have gone undetected in our model. Endogenous knockin strategies have been employed recently to examine the intricate dynamics of other cell-cell junction types in situ via the recent generation of ZO-1-GFP mice (Foote et al., 2013). Here, in line with our current work on adherens junctions, the authors report fundamental differences in tight junction dynamics in epidermal tissue in vivo, which cannot be recapitulated using 2D culture as a surrogate for living tissue. However, others have shown that E-cadherin can be differentially regulated within distinct points on the membrane, which could potentially be associated with zona adherens crosstalk (Priya et al., 2013).

In conclusion, FRAP in the E-cadherin-GFP mouse provides a flexible approach to monitor the intricate control of E-cadherin junctions in distinct biological processes in a time-, location-, and signaling-specific context. Future applications of this mouse as a tool alone or in combination with deficiencies of E-cadherin regulators and other tight junction components will profoundly expand our insights into the networks that govern E-cadherin responses in developmental, normal, or disease states while guiding therapeutic intervention strategies aimed at targeting cell-cell junctions during disease progression.

## Experimental Procedures

### Generation of E-cadherin-GFP Mice

A cDNA encoding EGFP-linked E-cadherin fusion protein (Lock and Stow, 2005) was cloned into pHPRT.CAAG.STOP downstream of a lox-stop-lox cassette, and the transgene was recombineered into pSKB1 (Bronson et al., 1996) to generate the final targeting vector. Upon germline transmission, the lox-stop-lox-E-cadherin-GFP mice (OFF mice) were bred with tissue-specific Cre driver lines or with CMV-Cre “deleter” mice to generate E-cadherin-GFP ON mice with ubiquitous E-cadherin-GFP expression.

All animal work was carried out under the control of the British Home Office and the local Animal Welfare and Experimental Ethics Committee (Cancer Research UK Beatson Institute and the Garvan Institute of Medical Research/St. Vincent’s Hospital Animal Ethics Committee).

### Confocal Photobleaching Experiments in Cell Culture, Cell-Derived Matrix and In Vivo

Confluent cells were imaged on 35-mm glass bottom dishes at 37°C and 5% CO_2_ using a Leica DMI 6000 SP8 confocal microscope or an Olympus FV1000 confocal microscope with a SIM scanner (Serrels et al., 2009). For photobleaching (FRAP, FLIP) experiments in live xenograft tumors, mice were anesthetized using a combination of 1:1 hypnorm - H_2_O + 1:1 hypnovel - H_2_O, and the subcutaneous tumor was exposed surgically via a skin flap procedure (Serrels et al., 2009) on a 37°C heated stage. Mice were sacrificed within 4 hr of imaging.

Detailed protocols can be found in the Supplemental Experimental Procedures.

## Author Contributions

Methodology, Z.E., D.H., S.C.W., E.J.M., J.P.M., K.I.A., and P.T.; Software, S.C.W. and E.J.M.; Formal Analysis, Z.E., D.H., and S.C.W.; Investigation, Z.E., D.H., S.C.W., M.N., M.C.L., W.L., N.R., A.M., J.P.S., S.K., J.R.W.C., C.V., S.A.K., A.D.C., D.G., A.M., K.J.M., R.A.R., A.M.L., S.N.W., D.R.C., and J.P.M.; Resources, D.G., A.M., S.N.W., S.T.G., D.R.C., L.Z., H.H., E.C.H., P.W.G., C.J.O., T.R.J.E., D.S., O.J.S., and J.P.M.; Conceptualization, K.I.A. and P.T.

## Supplemental Information

Document S1. Supplemental Experimental Procedures and Figures S1–S6

Movie S1. Tissue-Specific and Induced Expression of E-cadherin-GFP, Related to Figure 1

Movie S2. Constitutive Expression of E-cadherin-GFP, Related to Figure 1

Movie S3. FRAP on CDMs, Related to Figure 2

Movie S4. FLIP on CDMs, Related to Figure 2

Movie S5. FRAP in Xenografts, Related to Figure 3

Movie S6. FLIP on Xenografts, Related to Figure 3

Movie S7. FRAP in Genetically Engineered Mouse Models of PDAC Formation and following Drug Treatment, Related to Figures 5 and 6

Document S2. Article plus Supplemental Information
