# Supplementary material for: Intravital FRAP Imaging using an E-cadherin-GFP Mouse Reveals Disease- and Drug-Dependent Dynamic Regulation of Cell-Cell Junctions in Live Tissue
Source: Cell Rep. 2015 Dec 24;14(1):152–67. doi: 10.1016/j.celrep.2015.12.020 (PMC4709331; doi:10.1016/j.celrep.2015.12.020)
Supplement: Document S1. Supplemental Experimental Procedures and Figures S1–S6 [file mmc1.pdf]

Cell Reports

Supplemental Information

Intravital FRAP Imaging using an E-cadherin-GFP Mouse Reveals Disease- and Drug-Dependent Dynamic Regulation of Cell-Cell Junctions in Live Tissue

Zahra Erami, David Herrmann, Sean C. Warren, Max Nobis, Ewan J. McGhee, Morghan C. Lucas, Wilfred Leung, Nadine Reischmann, Agata Mrowinska, Juliane P. Schwarz, Shereen Kadir, James R.W. Conway, Claire Vennin, Saadia A. Karim, Andrew D. Campbell, David Gallego-Ortega, Astrid Magenau, Kendelle J. Murphy, Rachel A. Ridgway, Andrew M. Law, Stacey N. Walters, Shane T. Grey, David R. Croucher, Lei Zhang,, Herbert Herzog, Edna C. Hardeman, Peter W. Gunning, Christopher J. Ormandy, T.R. Jeffry Evans, Douglas Strathdee, Owen J. Sansom, Jennifer P. Morton, Kurt I. Anderson, and Paul Timpson

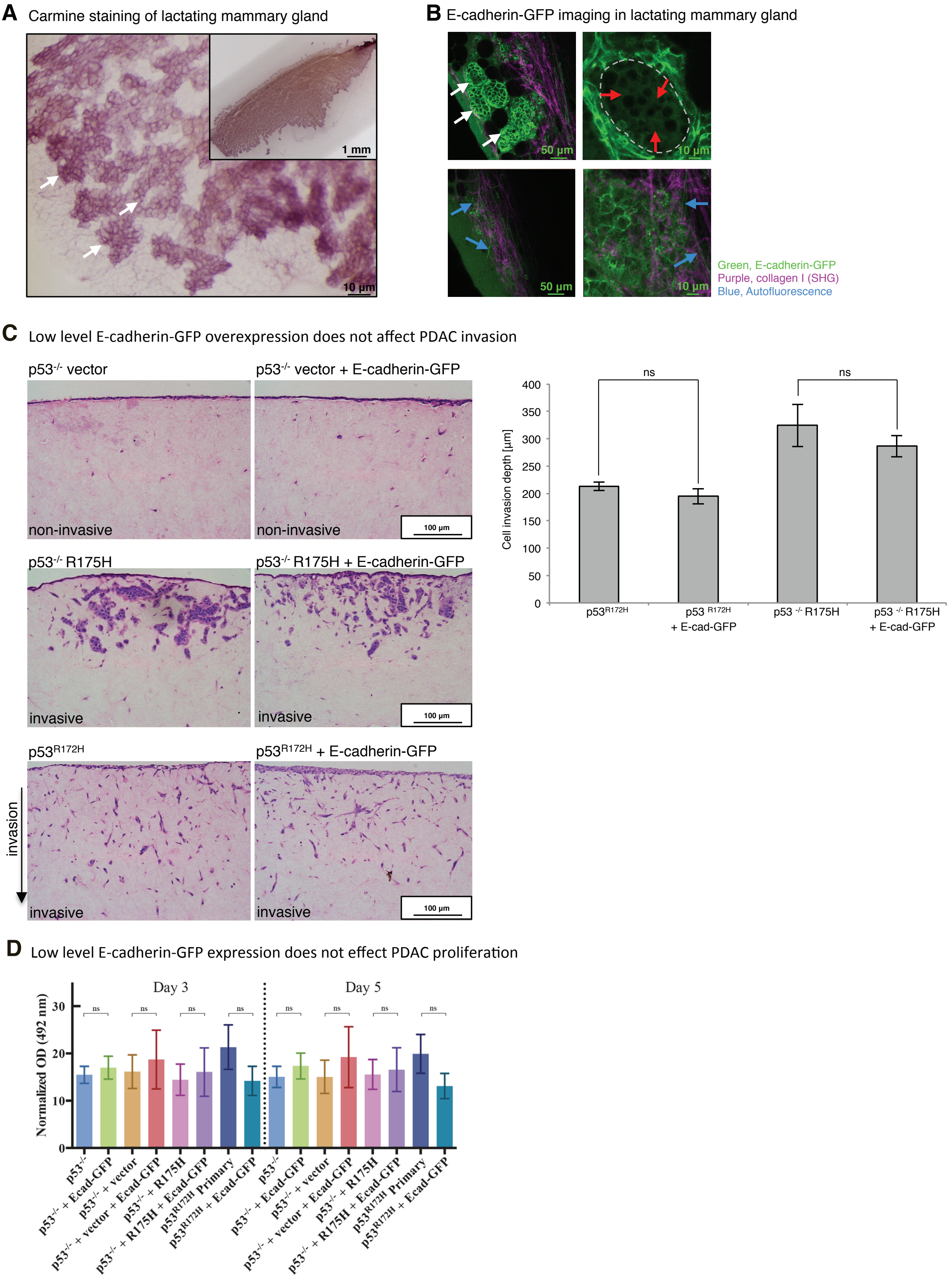
A
Carmine staining of lactating mammary gland
1 mm
10 µm
B
E-cadherin-GFP imaging in lactating mammary gland
50 µm
10 µm
50 µm
10 µm
Green, E-cadherin-GFP
Purple, collagen I (SHG)
Blue, Autofluorescence
C
Low level E-cadherin-GFP overexpression does not affect PDAC invasion
p53-/- vector
p53-/- vector + E-cadherin-GFP
non-invasive
non-invasive
100 µm
p53-/- R175H
p53-/- R175H + E-cadherin-GFP
invasive
invasive
100 µm
p53R172H
p53R172H + E-cadherin-GFP
invasion
invasive
invasive
100 µm
Cell invasion depth [µm]
400
350
300
250
200
150
100
50
0
ns
ns
p53R172H
p53 R172H + E-cad-GFP
p53 -/- R175H
p53 -/- R175H + E-cad-GFP
D
Low level E-cadherin-GFP expression does not effect PDAC proliferation
Normalized OD (492 nm)
30
20
10
0
Day 3
Day 5
ns
p53-/-
p53-/- + Ecad-GFP
p53-/- + vector
p53-/- + vector + Ecad-GFP
p53-/- + R175H
p53-/- + R175H + Ecad-GFP
p53R172H Primary
p53R172H + Ecad-GFP

Figure S1, related to Figure 1. Expressing E-cadherin-GFP in PDAC cells does not impair cell invasion and proliferation.

(A) Carmine staining of the whole-mounted lactating mammary gland (inset) showing fully developed alveoli (white arrows). (B) Representative still images of E-cadherin-GFP cell-cell junctions in the mammary gland at low (left panel) and high magnification (right panel). Expression is driven via MMTV-Cre. E-cadherin-GFP, green; SHG signal, purple. Note elastin autofluorescence (blue arrows). (C) Representative H&E images of primary PDAC cells on 3D-organotypic matrices with or without E-cadherin-GFP expression and quantification of cell invasion (bar, 100 μm). (D) Growth of primary cell lines from $p53^{-/-}$ and $p53^{R172H}$ tumors demonstrating that low-level expression of E-cadherin-GFP has no significant effect on tumor cell proliferation. Cells were stably transfected in combinations of vector alone, $p53^{R175H}$ and/or E-cadherin-GFP. Columns, mean; bars, ± SE. *, $P < 0.05$; **, $P < 0.01$ by unpaired Student's t-test.

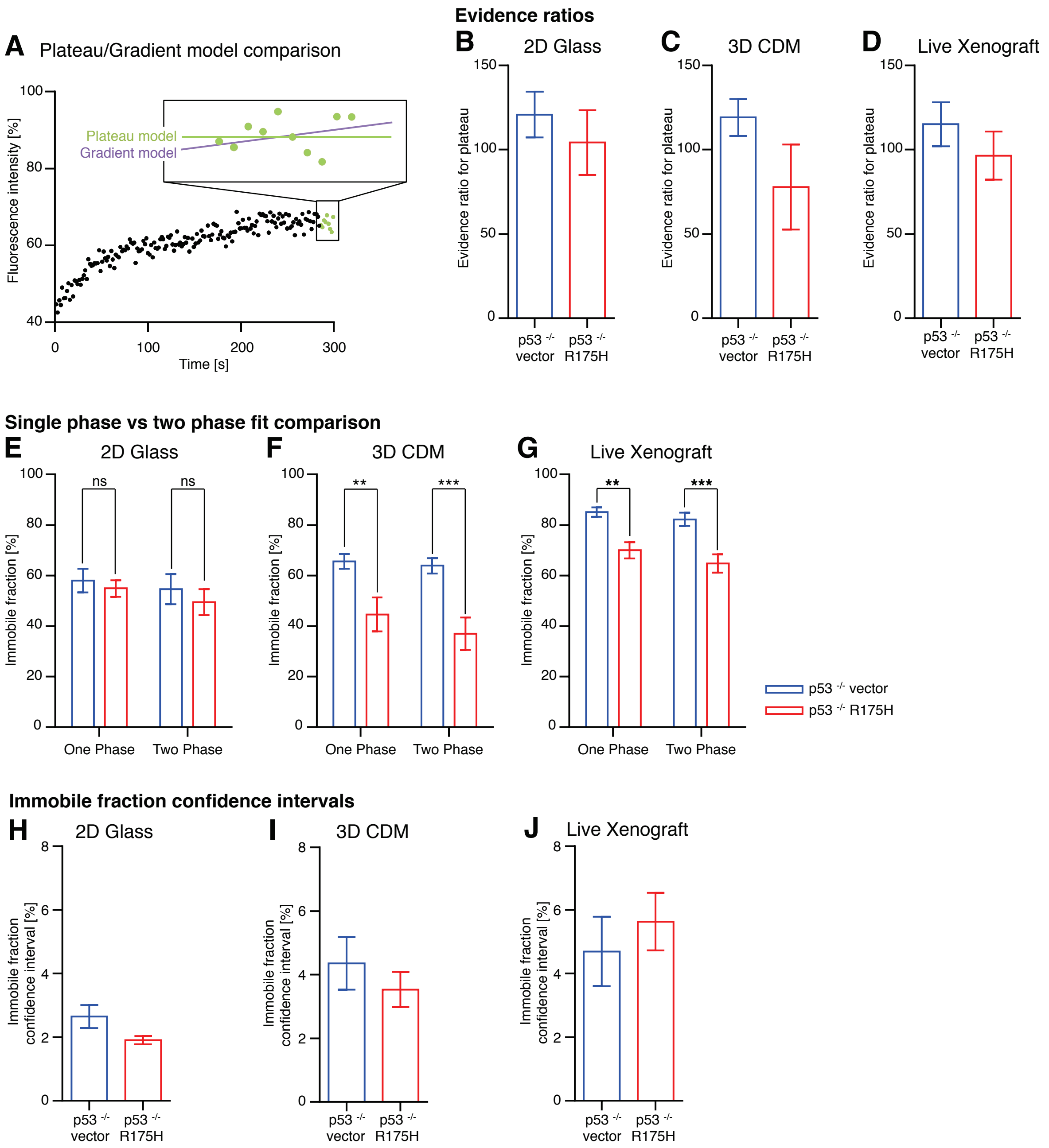
A
Plateau/Gradient model comparison
Plateau model
Gradient model
Fluorescence intensity [%]
Time [s]
40
60
80
100
0
100
200
300
Evidence ratios
B
2D Glass
C
3D CDM
D
Live Xenograft
Evidence ratio for plateau
0
50
100
150
p53 -/- vector
p53 -/- R175H
Single phase vs two phase fit comparison
E
2D Glass
F
3D CDM
G
Live Xenograft
Immobile fraction [%]
0
20
40
60
80
100
ns
ns
**
***
**
***
One Phase
Two Phase
p53 -/- vector
p53 -/- R175H
Immobile fraction confidence intervals
H
2D Glass
I
3D CDM
J
Live Xenograft
Immobile fraction confidence interval [%]
0
2
4
6
8
p53 -/- vector
p53 -/- R175H

Figure S2, related to Figures 2,3. Validation of FRAP quantification.

(A) Cartoon of approach used to determine the relative likelihood that a curve has reached a plateau (green line) or is continuing to rise (purple line) using model comparison. (B-D) Graph of evidence ratio for a plateau, compared to a gradient, for the final ten points in the FRAP recovery curve for $p53^{-/-}$ vector (blue) and $p53^{-/-}$ R175H cells (red) grown on glass (B), CDM (C) or in a xenograft (D). Evidence ratios larger than 1 indicate that it is more likely that the curve has plateaued. (E-G) Immobile fractions calculated when using one (left) and two phase FRAP recovery models (right) for $p53^{-/-}$ vector (blue) and $p53^{-/-}$ R175H cells (red) grown on glass (E), CDM (F) and in a xenograft (G). (H-J) Confidence interval on value of fitted immobile fraction calculated using the Delta method for $p53^{-/-}$ vector (blue) and $p53^{-/-}$ R175H cells plated on glass (H), CDM (I) and in a xenograft (J) Columns, mean; bars, ± SE. *, $P < 0.05$; **, $P < 0.01$; ***, $P < 0.001$ by unpaired Student's t-test.

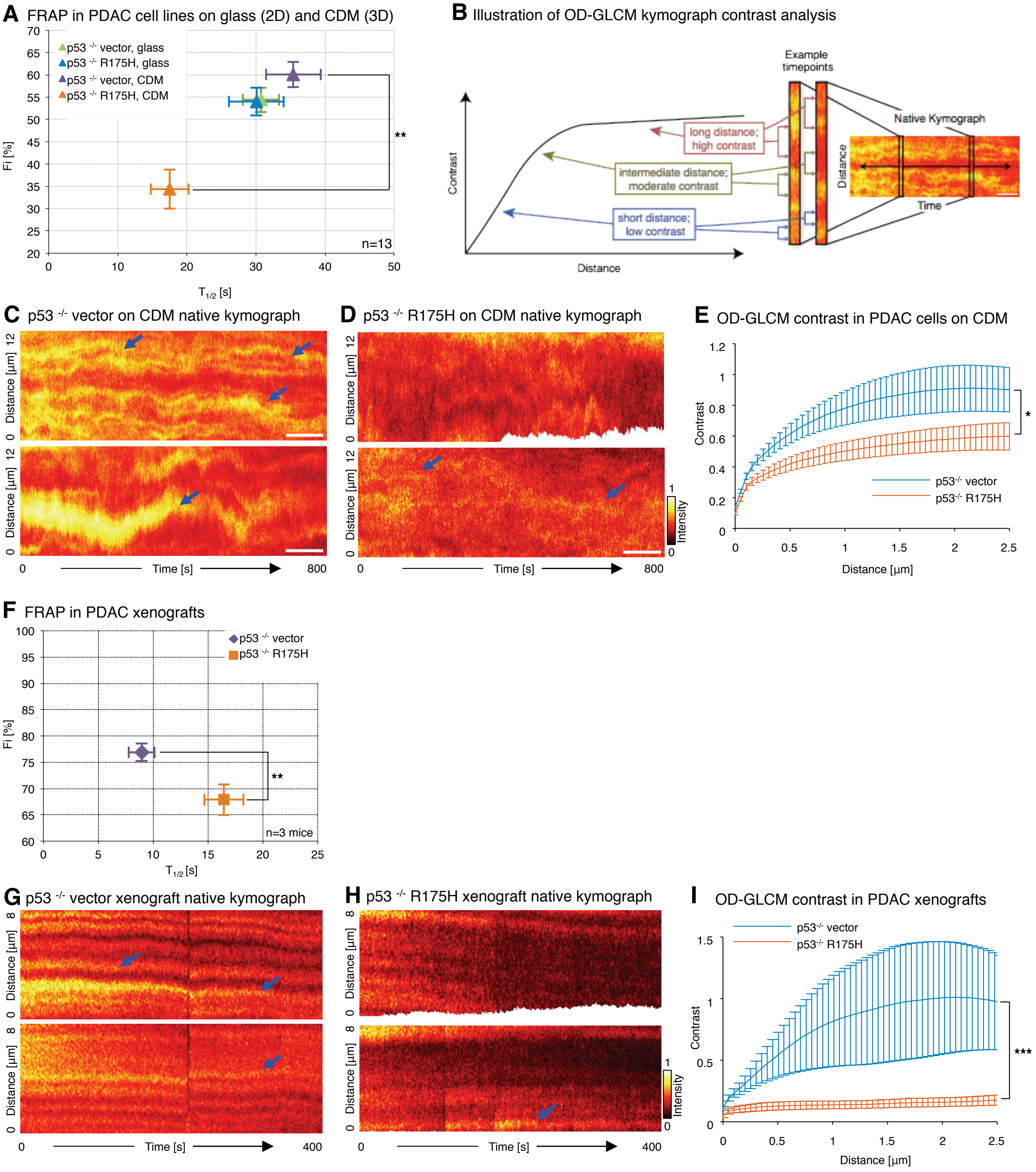
A FRAP in PDAC cell lines on glass (2D) and CDM (3D)
p53 -/- vector, glass
p53 -/- R175H, glass
p53 -/- vector, CDM
p53 -/- R175H, CDM
Fi [%]
$T_{1/2}$ [s]
n=13
**
B Illustration of OD-GLCM kymograph contrast analysis
Example timepoints
Native Kymograph
long distance; high contrast
intermediate distance; moderate contrast
short distance; low contrast
Contrast
Distance
Time
C p53 -/- vector on CDM native kymograph
D p53 -/- R175H on CDM native kymograph
Distance [µm]
Time [s]
Intensity
E OD-GLCM contrast in PDAC cells on CDM
p53-/- vector
p53-/- R175H
Contrast
Distance [µm]
*
F FRAP in PDAC xenografts
p53 -/- vector
p53 -/- R175H
Fi [%]
$T_{1/2}$ [s]
n=3 mice
**
G p53 -/- vector xenograft native kymograph
H p53 -/- R175H xenograft native kymograph
Distance [µm]
Time [s]
Intensity
I OD-GLCM contrast in PDAC xenografts
p53-/- vector
p53-/- R175H
Contrast
Distance [µm]
***

Figure S3, related to Figures 2,3. E-cadherin-GFP FRAP, FLIP and OD-GLCM analysis in non-invasive versus invasive PDAC cells on CDMs and in xenografts.

(A) Graphs comparing the mobilization of E-cadherin-GFP in cell-cell junctions between $p53^{-/-}$ vector and $p53^{-/-}$ R175H cells on CDMs. (B) Cartoon schematically illustrating the OD-GLCM kymograph contrast parameter. Each point on the graph gives a measure of the contrast between points along the junction at a particular distances from one another, averaged over the kymograph. This example shows two spatial profiles with specific examples illustrating how pairs of points contribute to the OD-GLCM contrast at short (blue), intermediate (yellow) and long (red) distances. (C,D) Representative kymographs of the native, unbleached junctions for $p53^{-/-}$ vector (C) and $p53^{-/-}$ R175H cells (D). Blue arrows indicate persistent regions of high intensity. Changes in the size of the kymograph represent slight changes in length of the junction over the imaging period. (E) OD-GLCM contrast as a function of distance for the $p53^{-/-}$ vector cells (blue, n=30) and $p53^{-/-}$ R175H cells (red, n=28). Columns, mean; bars, ± SE; *, $P < 0.05$; **, $P < 0.01$ by unpaired Student's t-test. (F) Graphs comparing the mobilization of E-cadherin-GFP in cell-cell junctions between $p53^{-/-}$ vector and $p53^{-/-}$ R175H cells on CDMs. (G,H) Representative kymographs of the native, unbleached junctions for $p53^{-/-}$ vector (G) and $p53^{-/-}$ R175H (H) xenografts. Blue arrows indicate persistent regions of high intensity. Slight discontinuities in the kymographs are due to movement of the imaging region to correct for drift. Changes in the size of the kymograph represent slight changes in length of the junction over the imaging period. (I) GLCM contrast as a function of distance for the $p53^{-/-}$ vector (blue, n=18) and $p53^{-/-}$ R175H xenografts (red, n=10). Statistical significance for OD-GLCM was calculated based on the average contrast. Columns, mean; bars, ± SE; *, $P < 0.05$; **, $P < 0.01$ by unpaired Student's t-test.

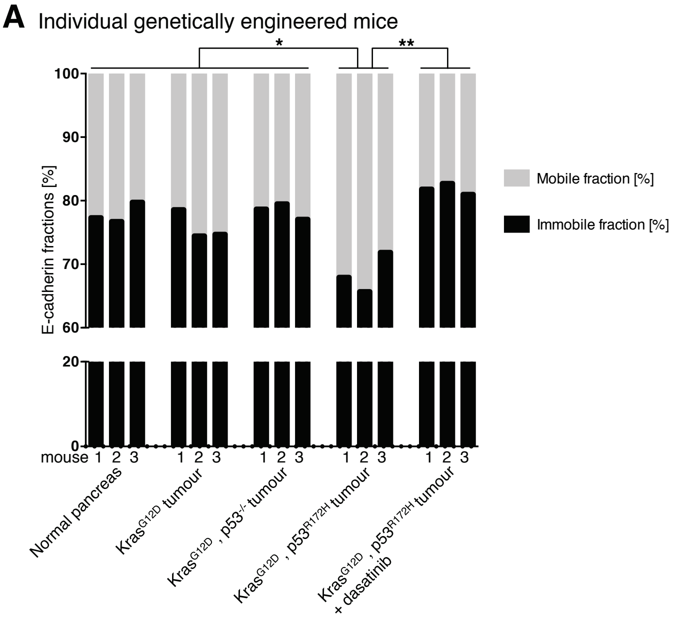

		Immobile fraction [%]	Mobile fraction [%]
**Wildtype pancreas**	mouse 1	77.3	22.7
	mouse 2	76.7	23.3
	mouse 3	79.7	20.3
**$Kras^{G12D}$ tumour**	mouse 1	78.6	21.4
	mouse 2	74.5	25.5
	mouse 3	74.7	25.3
**$Kras^{G12D}$; $p53^{-/-}$ tumour**	mouse 1	78.7	21.3
	mouse 2	79.5	20.5
	mouse 3	77.1	22.9
**$Kras^{G12D}$; $p53^{R172H}$ tumour**	mouse 1	67.9	32.1
	mouse 2	65.7	34.3
	mouse 3	71.9	28.1
**$Kras^{G12D}$; $p53^{R172H}$ tumour + dasatinib**	mouse 1	81.8	18.2
	mouse 2	82.7	17.3
	mouse 3	81.0	19.0

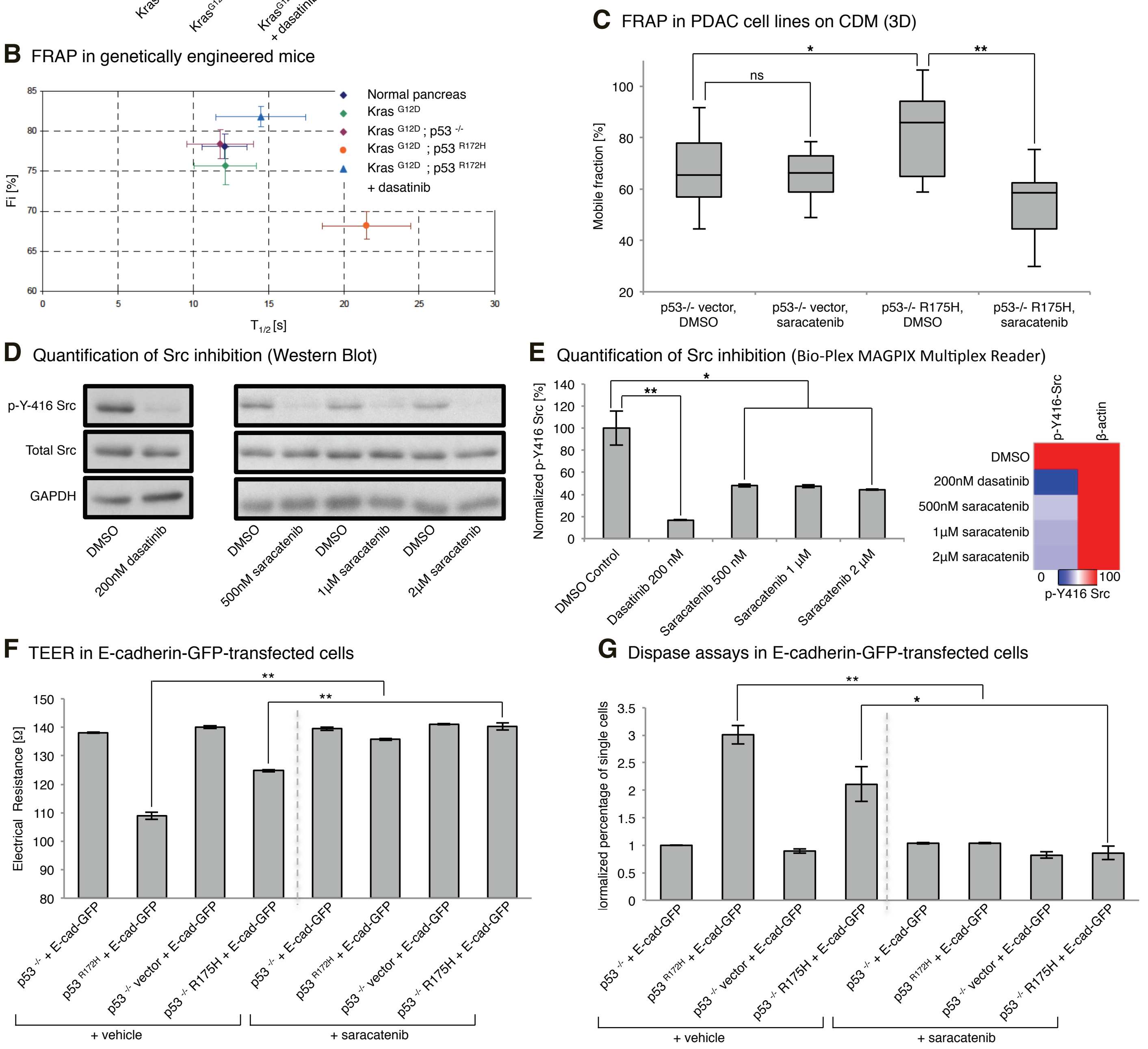

Figure S4, related to Figures 5,6. E-cadherin-GFP FRAP in *in situ* pancreatic tissue.
(A) Graph and table showing an increase in E-cadherin mobility in $Kras^{G12D}$; $p53^{R172H}$ tumors compared to normal pancreas, tumors of $Kras^{G12D}$ alone, $Kras^{G12D}$; $p53^{-/-}$ $Kras^{G12D}$; $p53^{R172H}$ and tumors of dasatinib-treated $Kras^{G12D}$; $p53^{R172H}$ mice for each mouse analyzed. (B) Graphs comparing the mobilization of E-cadherin-GFP in cell-cell junctions between normal pancreas, tumors of $Kras^{G12D}$ alone, $Kras^{G12D}$; $p53^{-/-}$ $Kras^{G12D}$; $p53^{R172H}$ and tumors of dasatinib-treated $Kras^{G12D}$; $p53^{R172H}$ mice (C) Graphs comparing the mobilization of E-cadherin-GFP in cell-cell junctions between $p53^{-/-}$ vector and $p53^{-/-}$ R175H cells grown on CDMs after treatment with 2μM saracatenib or DMSO for 4h; > 10 junctions per group. (D,E) Assessment of phospho-Y416 Src following treatment of $p53^{-/-}$ R175H cells with dasatinib or saracatenib for 4 hours with Western Blot analysis (D) and using Bio-Plex MAGPIX Multiplex Reader (E). (F,G) Trans-Endothelial Electrical Resistance (TEER) measurements of cell-cell junction integrity (F) and Dispase-based mechanical disruption (G) in non-invasive, E-cadherin-GFP-transfected $p53^{-/-}$ PDAC cells derived from Pdx1-Cre; $Kras^{G12D/+}$; $p53^{-/-}$ mice (columns, 1 and 3, pre-saracatenib and columns, 5 and 7, post- saracatenib) versus invasive, E-cadherin-GFP-transfected mutant p53 bearing PDAC cells (columns, 2 and 4, pre- saracatenib and columns, 6 and 8, post-saracatenib). Columns, mean; bars, ± SE, n = 3 per group, *, $P < 0.05$; **, $P < 0.01$ by unpaired Student's t-test.

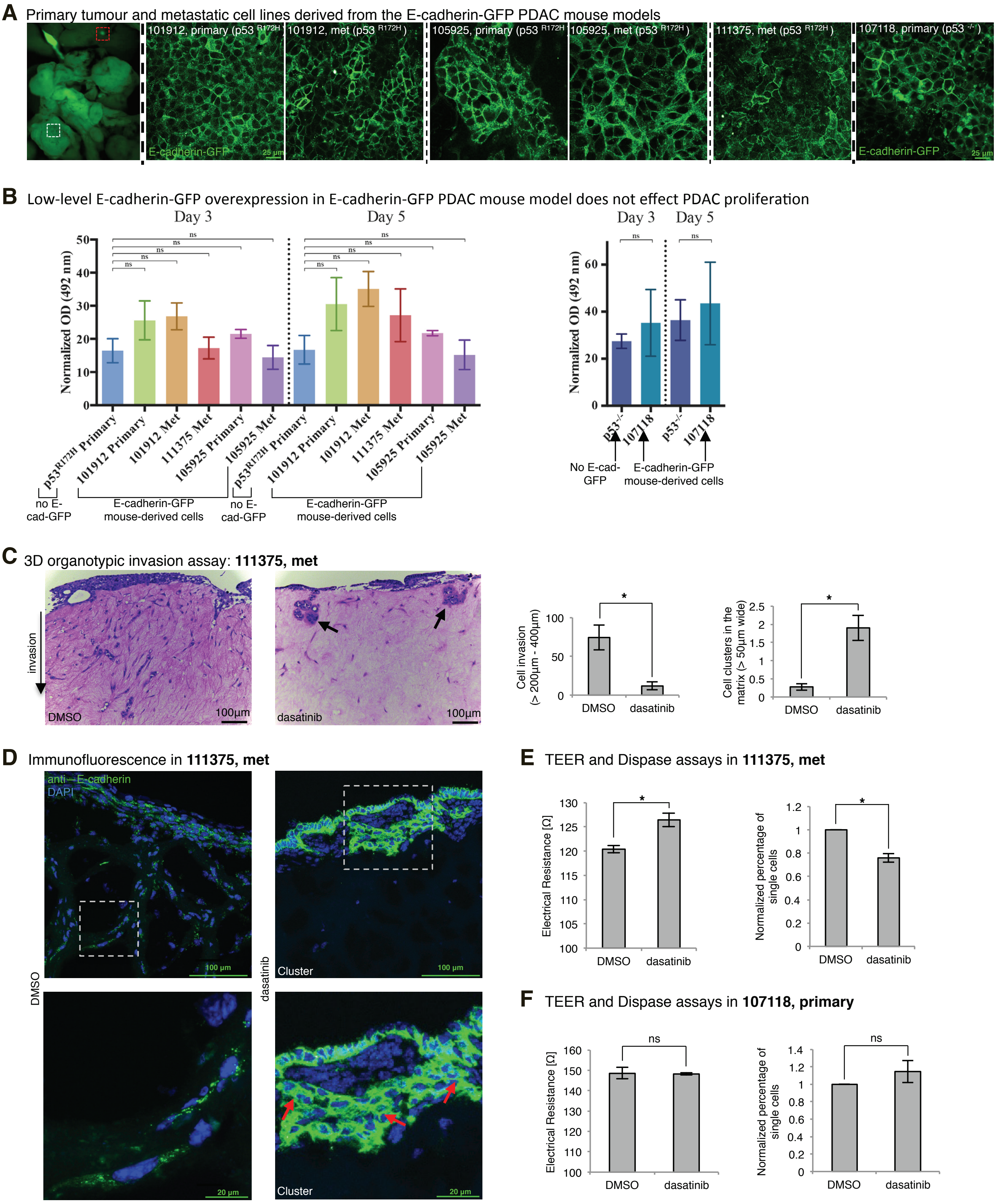
A Primary tumour and metastatic cell lines derived from the E-cadherin-GFP PDAC mouse models
101912, primary (p53 R172H)
101912, met (p53 R172H)
105925, primary (p53 R172H)
105925, met (p53 R172H)
111375, met (p53 R172H)
107118, primary (p53 -/-)
E-cadherin-GFP
25 µm
B Low-level E-cadherin-GFP overexpression in E-cadherin-GFP PDAC mouse model does not effect PDAC proliferation
Day 3
Day 5
Normalized OD (492 nm)
ns
p53R172H Primary
101912 Primary
101912 Met
111375 Met
105925 Primary
105925 Met
no E-cad-GFP
E-cadherin-GFP mouse-derived cells
p53-/-
107118
No E-cad-GFP
E-cadherin-GFP mouse-derived cells
C 3D organotypic invasion assay: 111375, met
invasion
DMSO
dasatinib
100µm
Cell invasion (> 200µm - 400µm)
Cell clusters in the matrix (> 50µm wide)
*
D Immunofluorescence in 111375, met
anti – E-cadherin
DAPI
DMSO
dasatinib
Cluster
100 µm
20 µm
E TEER and Dispase assays in 111375, met
Electrical Resistance [Ω]
Normalized percentage of single cells
F TEER and Dispase assays in 107118, primary
ns

Figure S5, related to Figure 7. E-cadherin-GFP expression in primary cells derived from Pdx1-Cre; Kras^{G12D}; p53R^{172H}; E-cadherin-GFP mice with dasatinib-induced cluster formation in 3D organotypic matrices.

(A) Whole body fluorescent image of primary tumor and isolated liver micro-metastases of a Pdx1-Cre; Kras^{G12D}; p53^{R172H}; E-cadherin-GFP mouse. Cell lines derived from the primary tumor and liver metastases of 3 independent mice (101912, 105925, 111375) as well as cells derived from the primary tunor of a Pdx1-Cre; Kras^{G12D}; p53$^{-/-}$; E-cadherin-GFP mouse (107118, primary) were chosen for further analysis. (B) Growth of primary cell lines from the Pdx1-Cre; Kras^{G12D}; p53^{R172H}; E-cadherin-GFP mouse was comparable to p53^{R172H} cells without E-cadherin-GFP expression (left panel). Growth of primary cell lines from the Pdx1-Cre; Kras^{G12D}; p53$^{-/-}$; E-cadherin-GFP mouse was comparable to p53$^{-/-}$ cells without E-cadherin-GFP expression (right panel). (C) Isolated secondary metastatic cell line (111375, met) from the liver of a Pdx1-Cre; Kras^{G12D}; p53^{R172H}; E-cadherin-GFP mouse invading into 3D-organotypic matrices ± dasatinib (13 days, 200 nM). Black arrows indicate cluster formation upon dasatinib treatment. Quantification of cell invasion as number of cells invaded beyond > 200 μm and quantification of cell clustering within matrices ± dasatinib (13 days, 200 nM). (D) Immunofluorescence staining of E-cadherin within matrices ± dasatinib (13 days, 200 nM) for metastatic line 111375 met. Red arrows indicate stabilized E-cadherin-GFP expression within cell clusters within the matrix. Anti-E-cadherin in green, DAPI in blue. (E,F) TEER measurements (left graph) and Dispase-based mechanical disruption (right graph) in 111375 met ± dasatinib (E) and 107118 ± dasatinib (F). Columns, mean; bars, ± SE; n = 3 per group; *, P <0.05 by unpaired Student's t-test.

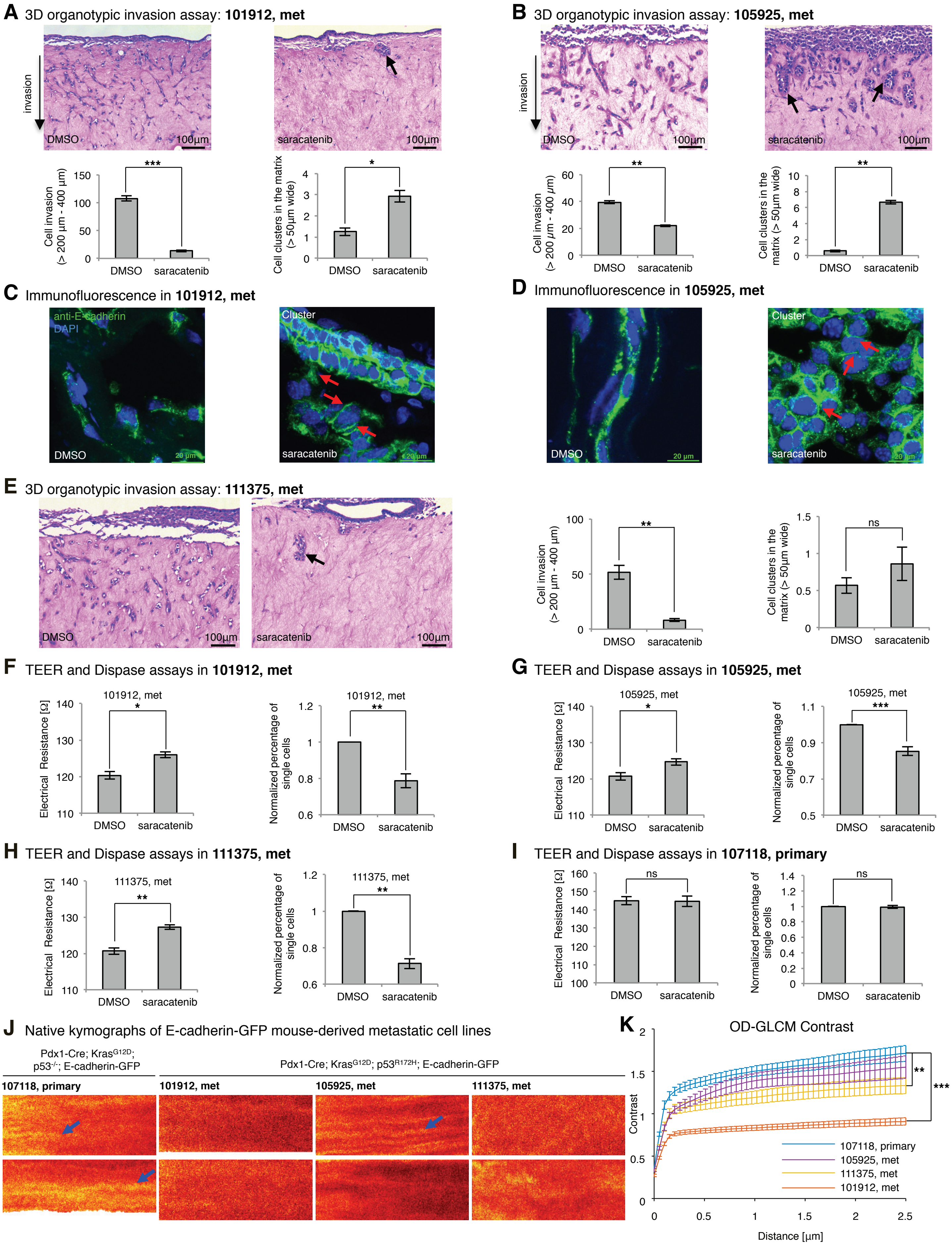
A 3D organotypic invasion assay: 101912, met
invasion
DMSO
saracatenib
100µm
Cell invasion (> 200 µm - 400 µm)
Cell clusters in the matrix (> 50µm wide)
B 3D organotypic invasion assay: 105925, met
C Immunofluorescence in 101912, met
anti-E-cadherin
DAPI
Cluster
20 µm
D Immunofluorescence in 105925, met
E 3D organotypic invasion assay: 111375, met
ns
F TEER and Dispase assays in 101912, met
Electrical Resistance [Ω]
Normalized percentage of single cells
101912, met
G TEER and Dispase assays in 105925, met
105925, met
H TEER and Dispase assays in 111375, met
111375, met
I TEER and Dispase assays in 107118, primary
J Native kymographs of E-cadherin-GFP mouse-derived metastatic cell lines
Pdx1-Cre; Kras^{G12D}; p53$^{-/-}$; E-cadherin-GFP
Pdx1-Cre; Kras^{G12D}; p53^{R172H}; E-cadherin-GFP
107118, primary
101912, met
105925, met
111375, met
K OD-GLCM Contrast
Contrast
Distance [µm]
107118, primary
105925, met
111375, met
101912, met

Figure S6, related to Figure 7. Saracatenib-induced cluster formation of metastatic cells derived from the E-cadherin-GFP mouse in 3D organotypic matrices and OD-GLCM junction analysis of cells derived from the E-cadherin-GFP mouse.

(A,B) Isolated metastatic cell lines 101912 met (A) and 105925 met (B) from the liver of the transgenic mouse invading in 3D-organotypic matrices ± saracatenib (13 days, 1μM). Black arrows indicate cluster formation upon saracatenib treatment. Quantification of cell invasion between 200 μm - 400 μm and cell clustering within matrices ± saracatenib (13 days, 1μM). (C,D) Immunofluorescence staining of E-cadherin within matrices ± saracatenib (13 days, 1μM) for metastatic lines 101912 met (C) and 105925 met (D). Red arrows label cell-cell junctions within cell clusters upon saracatenib treatment. E-cadherin, green; DAPI, blue. (E) Isolated secondary metastatic cell line 111375 met invading into 3D-organotypic matrices ± saracatenib (13 days, 1μM). Black arrows indicate cluster formation upon saracatenib treatment. Quantification of cell invasion as number of cells invaded beyond > 200 μm and quantification of cell clustering within matrices ± saracatenib (13 days, 1μM). (F-I) Trans-Endothelial Electrical Resistance (TEER) measurements (left) and Dispase-based mechanical disruption (right) in cell lines derived from the E-cadherin-GFP mouse, 101912 met ± saracatenib (F) and 105925 met (G), 111375 ± saracatenib (H) and 107118 ± saracatenib (I). (J) Representative kymographs of native, unbleached junctions for 107118 primary, 101912 met, 105925 met and 111375 met. Blue arrows indicate persistent regions of high intensity. Changes in the size of the kymograph represent slight changes in length of the junction over the imaging period. (K) OD-GLCM contrast as a function of distance for 107118 primary (blue, n=36), 101912 met (red, n=30), 105925 met (yellow, n=20) and 111375 met (purple, n=22). Statistical significance for OD-GLCM was calculated based on the average contrast. Columns, mean; bars, ± SE; n = 3 per group; *, $P < 0.05$; **, $P < 0.01$; ***, $P < 0.001$ by unpaired Student's t-test.

Supplemental Movie Legends

Movie S1, related to Figure 1. Tissue-specific and induced expression of E-cadherin-GFP.

First panel, Z-stack (left) and maximum-intensity 3D movie (right) of E-cadherin-GFP cell-cell junctions in the pancreas (Pdx1-Cre; E-Cadherin-GFP), E-cadherin-GFP in green, collagen-derived SHG-signal in purple.

Second panel, Z-stack through the liver of an untreated (left) and treated (right, 3 doses of 2mg ß-naphthoflavone over 8 hours) Cyp1a1-Cre; E-Cadherin-GFP control mouse (Cyp1a1-Cre; E-Cadherin-GFP), E-cadherin-GFP in green, collagen-derived SHG-signal in purple.

Third panel, Z-stack of mammary gland alveoli (left) and mammary gland lumen (right; MMTV-Cre; E-Cadherin-GFP), E-cadherin-GFP in green, collagen I-derived SHG-signal in purple. Note elastin autofluorescence in striated green lines.

Fourth panel, Z-stack movie (left) and FRAP time lapse movie (right) of E-cadherin-GFP in isolated pancreatic islets (RIP-Cre; E-Cadherin-GFP), E-cadherin-GFP in green, collagen I-derived SHG-signal in purple. Red arrow indicates bleached region.

Movie S2, related to Figure 1. Constitutive expression of E-cadherin-GFP.

First panel, Z-stack movie (left) and FRAP time-lapse movie (right) of E-cadherin-GFP in the kidney (E-cadherin-GFP 'ON' mouse), E-cadherin-GFP in green, collagen I-derived SHG-signal in purple. Red arrow indicates bleached region.

Second panel, Z-stack movie (left) and FRAP time-lapse movie (right) of E-cadherin-GFP in the choroid epithelium of the brain (E-cadherin-GFP 'ON' mouse), E-cadherin-GFP in green, collagen I-derived SHG-signal in purple. Red arrow indicates bleached region.

Third panel, Z-stack movie (left) and FRAP time-lapse movie (right) of E-cadherin-GFP in the salivary gland (E-cadherin-GFP 'ON' mouse), E-cadherin-GFP in green, collagen I-derived SHG-signal in purple. Red arrow indicates bleached region.

Fourth panel, Z-stack movie (left) and FRAP time-lapse movie (right) of E-cadherin-GFP in the pancreas (E-cadherin-GFP 'ON' mouse), E-cadherin-GFP in green, collagen I-derived SHG-signal in purple. Red arrow indicates bleached region.

Fifth panel, Z-stack movie (left) and FRAP time-lapse movie (right) of E-cadherin-GFP in the liver (E-cadherin-GFP 'ON' mouse), E-cadherin-GFP in green, collagen I-derived SHG-signal in purple. Red arrow indicates bleached region.

Sixth panel, Z-stack movie (left) and FRAP time-lapse movie (right) of E-cadherin-GFP in the lactating mammary gland (E-cadherin-GFP 'ON' mouse), E-cadherin-GFP in green, collagen I-derived SHG-signal in purple. Red arrow indicates bleached region.

Movie S3, related to Figure 2. FRAP on CDMs

FRAP time-lapse in E-cadherin-GFP expressing $p53^{-/-}$ vector cells (left) and E-cadherin-GFP expressing $p53^{-/-}$ R175H cells (right) on 3D-CDM, E-cadherin-GFP in green. Red arrow indicates bleached region.

Movie S4, related to Figure 2. FLIP on CDMs

FLIP time-lapse imaging (top panel) and tracking of cell-cell junctions (bottom panel) in E-cadherin-GFP expressing $p53^{-/-}$ vector cells (left panel) and E-cadherin-GFP expressing $p53^{-/-}$ R175H cells (right panel) on 3D-CDM, E-cadherin-GFP in green. White rectangle indicates repeatedly bleached region.

Movie S5, related to Figure 3. FRAP in xenografts

FRAP time-lapse in E-cadherin-GFP expressing $p53^{-/-}$ vector cells (left) and E-cadherin-GFP expressing $p53^{-/-}$ R175H cells (right) in live xenograft tumors, E-cadherin-GFP in green. Red arrow indicates bleached region.

Movie S6, related to Figure 3. FLIP on xenografts.

FLIP time-lapse imaging (top panel) and tracking of cell-cell junctions (bottom panel) in E-cadherin-GFP expressing $p53^{-/-}$ vector cells (left panel) and E-cadherin-GFP expressing $p53^{-/-}$ R175H cells (right panel) in live xenograft tumors, E-cadherin-GFP in green. White rectangle indicates repeatedly bleached region.

Movie S7, related to Figures 5,6. FRAP in genetically engineered mouse models of PDAC formation and following drug treatment.

First panel, FRAP time-lapse in Pdx1-Cre; E-Cadherin-GFP pancreas (left), Pdx1-Cre; $Kras^{G12D}$; E-Cadherin-GFP tumors (middle) and Pdx1-Cre; $Kras^{G12D}$; $p53^{-/-}$; E-Cadherin-GFP tumors (right) of age-

matched mice (~ 150 days old), E-cadherin-GFP in green. Red arrow indicates bleached region.
Second panel, FRAP time-lapse in Pdx1-Cre; $Kras^{G12D}$; $p53^{R172H}$; E-Cadherin-GFP tumors (left) and Pdx1-Cre; $Kras^{G12D}$; $p53^{R172H}$; E-Cadherin-GFP tumors treated with dasatinib for 3 days (right) of age-matched mice (~ 150 days old), E-cadherin-GFP in green. Red arrow indicates bleached region.

Supplemental Experimental Procedures

Generation of E-cadherin-GFP mice Conditional E-cadherin-GFP expressing mice were generated by targeting a lox-stop-lox transgene under the control of a CAG promoter to the Hprt locus (Figure 1A), as described previously (Bronson et al., 1996; Samuel et al., 2009; Schachtner et al., 2012). The CAG lox-stop-lox transgene was constructed by firstly replacing the SA of plasmid pBigT (Srinivas et al., 2001) with the CAG promoter from pTurbo-Cre. The PGK promoter and the EM7 promoter sequence from pL452 were subsequently inserted into pBigT.CAAG by recombineering in E. coli (Liu et al., 2003). Short 5' and 3' arms homologous to sequences within the *Hprt* targeting plasmid pSKB1 (Bronson et al., 1996) were then inserted upstream and downstream of the expression cassette to generate pHprt.CAAG.STOP. A cDNA encoding the extensively validated EGFP-linked E-cadherin fusion protein ((Lock et al., 2005; Lock and Stow, 2005), a kind gift from Jennifer Stow, Institute for Molecular Bioscience, University of Queensland, St. Lucia, Queensland, Australia) was then cloned downstream of the lox-stop-lox cassette. The transgene was then recombineered into pSKB1 (Bronson et al., 1996) to generate the final targeting vector.

The vector was linearised and electroporated into *Hprt*-deficient HM1 ES cells, cultured on a DR4 mouse embryonic fibroblast feeder layer (Magin et al., 1992; Tucker et al., 1997). Homologous recombinants were selected in medium containing HAT supplement (Sigma). Correct targeting of the vector to the *Hprt* locus on both the 5' and 3' sides was confirmed using PCR on genomic DNA prepared from HAT-resistant colonies. Following identification of correctly targeted clones, mouse lines were derived from C57BL/6J blastocysts according to standard protocols. Germline transmission was identified by coat color and correct transmission of the transgene was confimed by PCR to the GFP sequences using the primers AAGTTCATCTGCACCACCG and TCCTTGAAGAAGATGGTGCG.

Animals All animals were kept in conventional animal facilities and monitored daily. Animal experiments were performed in accordance with U.K. Home Office guidelines and the Australian code of practice for the care and use of animals for scientific purposes. All mice were genotyped by Transnetyx (Cordova, TN).

Multiphoton Imaging Imaging was conducted on an inverted Leica SP8 microscope and the excitation source used was a Ti:Sapphire femtosecond pulsed laser (Coherent Chameleon Ultra II), operating at 80 MHz and tuned to a wavelength of 890 nm. Images were acquired with a 25x NA0.95 water objective. A dichroic filter (495nm) was used to separate the SHG signal from GFP emission, which were further selected with band pass filters (460/50 and 525/50, respectively). Intensity was recorded with external RLD HyD detectors. For z-stacks, images were acquired at a resolution of 1080x1080 pixels and a z-step size of 2.52 μm.

Confocal photobleaching experiments in cell culture, cell-derived matrix and *in vivo* $2.5x10^6$ confluent PDAC cells (E-cadherin-GFP-transfected cell lines) or $5x10^6$ confluent metastatic cells (derived from the E-cadherin-GFP mouse) were plated onto 35 mm glass bottom dishes (MatTek) and allowed to adhere and form cell-cell junctions for 36h prior to photobleaching analysis. Dishes coated with cell-derived matrix (Serebriiskii et al., 2008) were used for photobleaching on 3D substrates. Photobleaching experiments were performed using a Leica DMI 6000 SP8 Confocal microscope or an Olympus FV1000 Confocal microscope with SIM scanner (Serrels et al., 2009). Cells were maintained at 37 °C and imaging performed at 488 nm excitation using the following settings: pixel dwell time 4 μs/px, pixel resolution 512x512. For FRAP imaging, a circular area with 1.5μm diameter (FRAP imaging) or 3μm diameter (large region FRAP) bleached to approximately 50% of the original pre-bleach intensity was used in all experimental setups (Sprague and McNally, 2005). Photobleaching was achieved using a 405 nm laser, 20 μs/pixel dwell time for one frame (at > 70 μm depth *in vivo*). Images were captured every 1.6 s for ~ 5 minutes.

For FLIP imaging, a rectangular area (0.5μm wide parallel to the junction and 4μm long perpendicular to the junction) was bleached using a 405 nm laser (20 μs/pixel dwell time for one frame) followed by 10 post-bleach images acquired with 488 nm as described above. The bleach-postbleach protocol was repeated for 50 cycles to achieve robust FLIP. Images were captured every 1.6 s for ~ 20 minutes.

For photobleaching experiments in live xenograft tumors, $1x10^6$ PDAC cells suspended in 100 μL HBSS (Invitrogen) were subcutaneously injected into the rear flank of CD-1 nude mouse and allowed to grow tumors ~ 1-1.4 mm, in line with animal ethics guidelines. To permit imaging mice were anaesthetized using a combination of 1:1 hypnorm - H_2O + 1:1 hypnovel - H_2O. Following induction of anaesthesia the subcutaneous tumor was surgically exposed via skin flap procedure, as

previously described (Canel et al., 2010b; Serrels et al., 2009) and the mouse restrained on a 37 °C heated stage. Intravital FRAP and FLIP imaging was performed as described above and the mice were sacrificed after the experiment within 4 h of imaging. FRAP on pancreatic tissues and tumors was performed using similar parameters as described above upon ~ 150 days of tumor development with the appearance of swollen abdomen, cachexia or reduced mobility due to tumor burden (n = 3 independent mice, per condition, ≥ 21 cell-cell junctions in total).

FRAP data analysis Recovery curves obtained from fluorescent intensity measurements were exported for exponential curve fitting, as described previously (Canel et al., 2010a; Canel et al., 2010b; Serrels et al., 2009). Data were fit using the following exponential function: $y(t) = y_0 + a * (1 - \exp(-b * x))$. The immobile fraction (Fi) was calculated as follows using values derived from the curve fit: $Fi = 100 * \left(1 - \frac{a}{1-y_0}\right)$. The half-time of recovery was calculated using the formula $t_{1/2} = (\ln 2)/b$, where b was obtained from the exponential curve fit. We calculated the 95% confidence interval on the fitted value of the immobile fraction using the Delta method (Figure S2H-J control, blue bar; mutant, red bar (Oehlert, 1992).

Quality control and validation of FRAP quantification The following quality control criteria were applied to reject data corrupted by unacceptable experimental artefacts. To control for drift in the z-axis, the intensity in unbleached areas of the image were assessed. If the intensity in these areas fluctuated or drifted over the time course, the image was excluded from the analysis. Images that exhibited minor lateral drift were realigned using the ImageJ plugin StackReg (Thevenaz et al., 1998). Images where realignment failed, were excluded from the analysis. For each fitted curve, the quality of the fit was assessed using the sum-of-squares residual R^2. Curves exhibiting an unacceptably low signal-to-noise level, as defined by a value of R^2 below 0.9 *in vitro* and 0.7 *in vivo*, were rejected from the analysis.

The FRAP data were fitted using a one phase FRAP recovery model to estimate the E-cadherin immobile fraction, as discussed above and described previously (Canel et al., 2010a; Canel et al., 2010b; Serrels et al., 2009). If multiple processes contribute to the recovery then a multiple phase recovery model, $y(t) = y_0 + a * [1 - \sum_{i=1}^{n} c_i \exp(-b_i \cdot t)]$, may be used to quantify the recovery times of the different processes. The immobile fraction, Fi= $100 * \left(1 - \frac{a}{1-y_0}\right)$, in principle depends only on the depth of the initial bleach event, y_0, and the height of the recovery, a. If a measured FRAP curve has plateaued before the end of the measurement then the estimated immobile fraction does not depend on the number and rate of the processes in the model used to fit the decay. To validate our approach to estimating the E-cadherin immobile fraction, we a) verified that our FRAP curves had reached a plateau in the measurement period and b) compared the immobile fraction calculated using a one phase and two phase model.

a) To determine whether the FRAP curves had truly reached a plateau by the end of the recorded trace we compared the fit of two models, (i) a straight line fit with a slope equal to zero and (ii) a straight line fit with a positive gradient, to the final ten points of the FRAP curve using Akaike's information criteria (AIC) (Akaike, 1981; Motulsky and Christopoulos, 2004). These models correspond to a plateau and a continued gradient, respectively. The AIC for a model is given by

$$\mathrm{AIC} = \mathrm{N} \cdot \ln\left(\frac{\mathrm{SS}}{\mathrm{N}}\right) + 2\mathrm{K}$$

where SS is the sum-of-squares residuals of the fit, N is the number of data points and K the number of fitted parameters. The difference between the AIC of the two models, $\mathrm{AIC}_{\mathrm{gradient}}$-$\mathrm{AIC}_{\mathrm{plateau}}$ gives an insight into the relative probabilities of the two models given the data. In particular, the evidence ratio, gives an estimate of the ratio of probabilities of the two models (Figure S2A):

$$\text{Evidence Ratio} = \frac{\mathrm{P(plateau)}}{\mathrm{P(gradient)}} = \frac{1}{e^{-0.5(\mathrm{AIC}_{\mathrm{plateau}} - \mathrm{AIC}_{\mathrm{gradient}})}}$$

This analysis was applied to the *in vitro* and *in vivo* FRAP data. For each dataset, the average evidence ratio for a plateau was greater than 75, indicating that the plateau model was at least 75 times more likely than the gradient model and so providing strong evidence the data had become asymptotic and plateaued (Figure S2B-D).

b) We also compared the immobile fraction calculated by fitting the *in vitro* and *in vivo* FRAP data shown to a one phase and two phase model (Figure S2E-G). Where fitting with a two-phase model failed to converge, the datasets were excluded from the analysis. We found that fitting either a single or two-phase model to our data generated from 2D (glass), 3D (CDMs) or xenografts models made no significant difference to our analysis, indicating that use of a single exponential fit

does not bias our readout of the immobile fraction (Figure S2E-G control, blue bar; mutant, red bar) and is in line with previous publications (Canel et al., 2010a; Canel et al., 2010b; Serrels et al., 2009). We note that a two-phase analysis requires a significantly higher signal to noise ratio to reliably constrain the fit parameters (Istratov and Vyvenko, 1999) and therefore chose to use a single-phase model throughout our manuscript to determine the immobile fraction. The AIC analysis providing strong evidence that the curves have plateaued in combination with the comparison of the two models suggests that fitting a single exponential model provides an appropriate and robust estimate of the immobile fraction.

Tracking junctions during FLIP acquisition Junctions were tracked over time using an optical flow based procedure. Junctions were identified using a series of manually selected points. From these points, ten evenly spaced key points along the junction were obtained using spline interpolation (Burden and Faires, 1997). The optical flow between consecutive images in the sequence was calculated using the Horn-Schunck method (Barron et al., 1994) with the Matlab Vision toolbox. The optical flow provides an estimate of the velocity of objects in the image. The optical flow map was smoothed with a circular kernel of radius eight pixels and used to calculate the displacement of the key points along the junctions between the two frames to estimate the position of the junction in the latter frame. Ten new evenly spaced points were generated from the spline connecting the displaced points and the process was repeated for all consecutive pairs of images in the sequence. This approach allowed us to follow the intensity profile along a deforming junction for up to 800s.

FLIP data analysis Where sample drift was detected, the FLIP image sequences were aligned using rigid-body registration with the ImageJ plugin "StackReg" (Thevenaz et al., 1998). In each image five junctions were tracked; the bleached junction, two junction adjacent to the bleached region and two distant junctions in cells which were not bleached. The average intensity of the junctions was tracked with time to yield intensity time profiles for each junction. In addition, the average intensity in a 1μm region around the bleach point on the membrane and two points 3μm from the bleach point was calculated for each time point. Photobleaching due to imaging was corrected by fitting an exponential decay curve to the average of the distant junctions' time profiles and dividing each time profile by the fitted decay curve. The fraction of retained fluorescence was calculated using the ratio of the average of the final five points in the time profile to the initial intensity.

Generation of kymographs Kymographs were generated for tracked junctions. At each time point, 200 evenly spaced points along the junction were generated using spline interpolation (Burden and Faires, 1997). At each point, a ten pixel line perpendicular to and centered on the junction was integrated to estimate the intensity of the junction at that point and generate a line profile of the intensity along the junction. Where the length of the tracked junction changed, the line profiles were resampled to match the spacing in the first frame. Using this analysis we were able to visualise the loss of fluorescence across a junction with time as a single point is bleached.

Kymograph GLCM analysis To assess the level of intensity variation across a junction and quantify the uniformity or texture of the E-cadherin signal, Orientation Dependent Gray-Level Co-occurrence Matrix (OD-GLCM) analysis (Haralick et al., 1973) was applied to kymographs of native (unbleached) junctions. A GLCM quantifies the number of occurrences of different intensity values at a particular spatial offset over an image and can be used to quantify the heterogeneity of an image, as previously demonstrated (Hu et al., 2012; Huo et al., 2015; Miller et al., 2015; Nobis et al., 2013; Samuel et al., 2011). The contrast parameter, $\sum_{i,j}|i-j|^2\, p(i,j)$, provides a measure of contrast between a pixel and its neighbour over the whole kymograph and serves as an indication of the differences in intensity values along the junction; a low contrast value indicates a homogeneous intensity distribution, while a high contrast value indicates areas of high and low intensity are present (Figure S3B). The contrast parameter was calculated over a range of offsets from zero to 80 pixels along the junction.

Source code for the Kymograph generation and FLIP analysis used in the manuscript is available to download from https://github.com/timpsonlab/

Large area FRAP analysis Kymographs of large region FRAP movies were generated using the approach described above. To generate an average kymograph, the kymographs were aligned based on the location of the bleach region and resampled on a uniform grid of 200 points spaced over 10μm around the bleach region. The initial intensity of the junction was corrected for by normalising each

spatial point on the kymograph to its corresponding pre-bleach intensity. Photobleaching due to imaging was corrected by fitting an exponential decay curve to the average intensity of the movie outside of the bleach region and normalising the kymographs to the fitted curve. Selected spatial profiles from the average, normalised kymograph were fitted to a Gaussian profile $I(r) = 1 - A \cdot \exp\left(-\frac{(r-r_0)}{\sigma^2}\right)$ using the Matlab curve fitting toolbox to find the width σ and depth A of the bleached region at that point.

Analysis of large area FRAP kymographs The change in the intensity profile along the membrane of a FRAP bleach region over time can provide an insight into the processes governing the recovery. To quantify this process, we used a model of FRAP recovery considering the effects of lateral diffusion in the membrane and exchange with the cytoplasm (de Beco et al., 2012; de Beco et al., 2009; Goehring et al., 2010) and relate this model to the parameters of the Gaussian fit of the measured spatial profiles. The rate equation which governs the recovery of a photobleaching event in the membrane $c^*(r,t)$, can be written in terms of the effective diffusion coefficient D_{eff} , and the rate constant of endocytosis from the cytoplasm to the membrane, k_{endo}, as

$$\frac{dc^*}{dt} = D_{eff}\nabla^2 c^* - k_{endo}c^*$$

assuming a reaction limited exchange process where diffusion in the cytoplasm is fast compared the rate of endo- and exocytosis (Coscoy et al., 2002). If the initial bleach event has a Gaussian profile with width a, it may be shown by substitution that the bleach profile will evolve according to

$$c^*(x,t) = \frac{\exp(-k_{endo}t)}{\sqrt{a^2 + 4D_{eff}t}} \exp\left(-\frac{r^2}{a^2 + 4D_{eff}t}\right),$$

a Gaussian with width characterised by $\sigma^2 = a^2 + 4D_{eff}t$ whose height decreases over time due to both diffusion and transport. If the recovery is dominated by cytoplasmic exchange, the width of the bleach region will remain uniform during the recovery as illustrated in Figure S4B. If diffusion is present, the width of the bleach region will increase over time. Simulations of recovery via diffusion and cytoplasmic-exchange are shown as line profiles and kymographs in Figure S4A,B. To estimate the diffusion coefficient, we fitted the spatial bleach profiles over time to the equation above. The diffusion coefficient was estimated from a straight line fit of σ^2 against time. Using this approach we were able to monitor and gain an insight into the contributions of lateral motion and cytoplasmic transport to the FRAP recovery process.

Drug treatment *in vitro* and *in vivo* Dasatinib (Bristol-Myers Squibb, Princeton, NJ) was administered daily by oral gavage in 80 mM citrate buffer (10 mg/kg) (or 200 nM in cell culture medium *in vitro*). Saracatenib (Assay Matrix) was used at a concentration of 2μM in cell culture medium *in vitro*. After pancreatic tumor burden was observed in mice (monitored for swollen abdomen, cachexia, reduced mobility with tumor evident by palpation), the respective animals were then administered dasatinib for three days, as previously described (Morton et al., 2010; Nobis et al., 2013). After the final treatments the tumors were subsequently imaged and FRAP recovery curves obtained, n = 3 mice per imaging group (Morton et al., 2010; Nobis et al., 2013).

Whole body OV100 imaging and cell culture Primary tumor cells from the pancreas of non-invasive Pdx1-Cre; $Kras^{G12D}$; $p53^{-/-}$; E-cadherin-GFP and invasive Pdx1-Cre; $Kras^{G12D}$; $p53^{R172H}$; E-cadherin-GFP mice or secondary metastatic tumor cells which had colonized in the liver were identified using whole body OV100 fluorescent imaging (Olympus) and surgically removed prior to disaggregation *in vitro* and outgrowth in growth media (Dulbecco's modified Eagle medium containing 10% fetal bovine serum and 2 mmol/L L-glutamine) to generate cell lines 107118 primary, 101192 primary, 101912 met, 105925 primary, 105925 met and 111375 met, as previously achieved (Morton et al., 2010). Similarly, $p53^{R172H}$ and $p53^{-/-}$ PDAC cell lines were derived from tumors harvested from Pdx1-Cre; $Kras^{G12D}$; $p53^{R172H}$ mice and Pdx1-Cre; $Kras^{G12D}$; $p53^{flox/+}$ mice (which have lost wildtype p53 expression and are referred to as $p53^{-/-}$ cells throughout the manuscript), respectively (Morton et al., 2010). $p53^{-/-}$ R175H and $p53^{-/-}$ vector cells were generated by stable transfection of $p53^{-/-}$ cells with $p53^{R175H}$ or empty vector, and selected using 0.6 mg/ml G418 (Formedium, gift from Prof Karen Vousden). Subsequently, each cell line was stably transfected with E-cadherin-GFP plasmid (Lock and Stow, 2005; Serrels et al., 2009) and selected using 100 μg/ml hygromycin. Cells were maintained in DMEM supplemented with 10 % FCS + 2 mM L-glutamine + 1 % penicillin/streptomycin solution (Invitrogen) and sub-cultured weekly at a split ratio of 1:10 (Morton et al., 2010).

Proliferation assay 1,000 cells were plated in 96-well microtiter plates (six replicates per cell line) and incubated overnight to allow attachment prior to the day 0 time point. All cell lines were cultured as described above. Cell proliferation was determined at day 3 and day 5 using the CellTiter 96 aqueous assay (Promega), in accordance with manufacturer's instructions, and normalized to day 0 after blank correction.

Cell adhesion measurement via TEER and Dispase assay TEER was measured on confluent PDAC cells seeded into 12 well transwell supports (costar) using an EVOM2 epithelial voltohmmeter with an STX2 electrode (World Precision Instruments). The results were reported as electrical resistance (Ohms) ± SEM, P < 0.05 *, P < ** 0.01, unpaired Student's t-test. For Dispase assays (Calautti et al., 1998), confluent PDAC cells were treated with 6 mg/ml Dispase II (Sigma) in PBS, and subjected to mechanical stress, as previously described (Calautti et al., 1998; Canel et al., 2010b). Single cells were counted after passing through a 40 um nylon filter (BD Falcon). The single cell number was calculated as a percentage of the total cell number achieved after treating a control well with trypsin. Primary metastatic lines were treated with 100nM dasatinib or 1μm saracatenib for 2h. Results were reported by normalizing the drug-treated samples to the corresponding vehicle-treated samples. Columns, mean ± SEM, P < 0.05 *, P < ** 0.01, unpaired Student's t-test.

3D rat tail fibrillar collagen I organotypic assay Briefly, ~ 3 x 10^5/ml primary fibroblasts were embedded in a three-dimensional matrix of rat tail collagen I. Rat tail tendon collagen solution was prepared by the extraction of tendons with 0.5 M acetic acid to a concentration of ~ 2 mg/ml. Detached, polymerized matrix (2.5 ml) in 35 mm petri dishes was allowed to contract for approximately 12 days in complete media (DMEM, supplemented with 10% FCS, Invitrogen) until the fibroblasts had contracted the matrix to ~1.5 cm diameter. Subsequently, 1 x 10^5 PDAC cells were plated on top of the matrix in complete media and allowed to grow to confluence for 4 days. The matrix was then mounted on a metal grid and raised to the air/liquid interface resulting in the matrix being fed from below with complete media that was changed every 2 days. For drug treatment, 200 nM dasatinib or 1μM saracatenib was added to the media below the matrix throughout the 13 day invasion period. After 13 days, the cultures were imaged as described, snap-frozen for cryo-sectioning or fixed using 10% formalin and processed by standard methods for hematoxylin and eosin (H&E). PDAC cell invasive index was determined by counting cells that invaded between 200 μm and 400 μm within the matrix, as previously described using 10 independent images per condition, n= 3 (Nobis et al., 2013; Reverter et al., 2014; Timpson et al., 2011). Similarly, cell clustering upon dasatinib treatment was quantified by counting cell clusters > 50 μm throughout the matrix, as previously described (Dawson et al., 2012). Representative images of 3 independent experiments are shown. Columns, mean ± SEM, *, P <0.05; **, P < 0.01, unpaired Student's t-test.

Immunoblot Cells and tissues were lysed in protein extraction buffer (50mM HEPES, 1% Triton-X-100, 0.5% sodium deoxycholate, 0.1% SDS, 0.5mM EDTA, 50mM NaF, 10mM Na_3VO_4 and protease inhibitor cocktail (Roche)). Lysates were resolved by Bis-Tris gel electrophoresis and proteins transferred onto a PVDF membrane (Immobilon-P, Millipore), blocked with 5% milk, incubated with primary antibodies against E-Cadherin (BD Biosciences, 1:1000), phospho-Y416 Src (Cell Signaling, 1:500), total Src (Cell Signaling, 1:1000), β-actin (SigmaAldrich, 1:40000) and GAPDH (Acris, 1:10000) diluted in 5% BSA overnight at 4 °C and probed with HRP-linked secondary antibodies (GE Healthcare, 1:5000) diluted in 1% milk. Signal was visualized with ECL Reagent (Pierce) on X-Ray films (Fujifilm). Cell lysates were analysed using a Bio-Plex MAGPIX Multiplex Reader (BioRad), according to manufacturer's instructions. Assays for the measurement of p-Src-Y416 (171V50039M, BioRad)) and β-Actin (171V60020M, BioRad) were used. Values obtained for p-Y416 Src were normalised to β-actin (mean +/- SEM, n=3).

Immunofluorescence For E-cadherin immunofluorescence, organotypic cryo sections were de-frosted, fixed in 4% PFA and permeabilized in ice-cold methanol. Subsequently, sections were blocked with 10% FBS and incubated with anti-E-cadherin (BD Biosciences) diluted 1:100 in 1% BSA overnight at 4°C. Sections were incubated with Alexa488-coupled anti-mouse antibody (Jackson ImmunoResearch Laboratories Inc.), counter-stained with DAPI and mounted with Vectashield (Vector). For detection of E-cadherin-GFP signal from E-cadherin-GFP mouse-derived cells, primary cell lines were grown on cover slips. Upon confluence, cells were fixed with 4% PFA for 10min and mounted with Vectashield (Vector) for direct GFP imaging via fluorescence.

Carmine and immunohistochemistry staining Mammary glands were whole mounted and fixed in

10% buffered formaline. Staining was performed using carmine alum. 4µm mammary gland sections were baked for 5 min at 80°C and placed in Xylene for de-paraffinization. Antigen retrieval was performed using target retrieval solution low-pH (S1699) 20-min water bath (α-milk, Accurate Chemical and Scientific Corp.); high-pH (S2367) 20-min water bath (α-GFP; Life Technologies). For immunohistochemistry, slides were quenched in 3% H_2O_2 prior to application of 1:12,000 α-milk, and protein block (Dako) 10 min prior application of 1:200 α-GFP. Secondary antibodies (Envision) were applied for 30 min. Visualization was achieved with diaminobenzidine (DAB+). All immunohistochemistry reagents were purchased from Dako Cytomation unless otherwise stated.

Supplemental References

Akaike, H. (1981). Likelihood of a Model and Information Criteria. J Econometrics *16*, 3-14.

Barron, J.L., Fleet, D.J., and Beauchemin, S.S. (1994). Performance of Optical-Flow Techniques. Int J Comput Vision *12*, 43-77.

Burden, R.L., and Faires, J.D. (1997). Numerical analysis, 6th edn (Pacific Grove, CA: Brooks/Cole Pub. Co.).

Coscoy, S., Waharte, F., Gautreau, A., Martin, M., Louvard, D., Mangeat, P., Arpin, M., and Amblard, F. (2002). Molecular analysis of microscopic ezrin dynamics by two-photon FRAP. Proceedings of the National Academy of Sciences of the United States of America *99*, 12813-12818.

Dawson, J.C., Timpson, P., Kalna, G., and Machesky, L.M. (2012). Mtss1 regulates epidermal growth factor signaling in head and neck squamous carcinoma cells. Oncogene *31*, 1781-1793.

de Beco, S., Gueudry, C., Amblard, F., and Coscoy, S. (2009). Endocytosis is required for E-cadherin redistribution at mature adherens junctions. Proceedings of the National Academy of Sciences of the United States of America *106*, 7010-7015.

Goehring, N.W., Chowdhury, D., Hyman, A.A., and Grill, S.W. (2010). FRAP analysis of membrane-associated proteins: lateral diffusion and membrane-cytoplasmic exchange. Biophysical journal *99*, 2443-2452.

Haralick, R.M., Shanmuga.K, and Dinstein, I. (1973). Textural Features for Image Classification. Ieee T Syst Man Cyb *Smc3*, 610-621.

Huo, C.W., Chew, G., Hill, P., Huang, D., Ingman, W., Hodson, L., Brown, K.A., Magenau, A., Allam, A.H., McGhee, E.*, et al.* (2015). High mammographic density is associated with an increase in stromal collagen and immune cells within the mammary epithelium. Breast cancer research : BCR *17*, 79.

Istratov, A.A., and Vyvenko, O.F. (1999). Exponential analysis in physical phenomena. Rev Sci Instrum *70*, 1233-1257.

Liu, P., Jenkins, N.A., and Copeland, N.G. (2003). A highly efficient recombineering-based method for generating conditional knockout mutations. Genome Res *13*, 476-484.

Lock, J.G., Hammond, L.A., Houghton, F., Gleeson, P.A., and Stow, J.L. (2005). E-cadherin transport from the trans-Golgi network in tubulovesicular carriers is selectively regulated by golgin-97. Traffic *6*, 1142-1156.

Magin, T.M., McWhir, J., and Melton, D.W. (1992). A new mouse embryonic stem cell line with good germ line contribution and gene targeting frequency. Nucleic Acids Res *20*, 3795-3796.

Miller, B.W., Morton, J.P., Pinese, M., Saturno, G., Jamieson, N.B., McGhee, E., Timpson, P., Leach, J., McGarry, L., Shanks, E.*, et al.* (2015). Targeting the LOX/hypoxia axis reverses many of the features that make pancreatic cancer deadly: inhibition of LOX abrogates metastasis and enhances drug efficacy. EMBO molecular medicine *7*, 1063-1076.

Motulsky, H., and Christopoulos, A. (2004). Fitting models to biological data using linear and nonlinear regression : a practical guide to curve fitting (Oxford ; New York: Oxford University Press).

Oehlert, G.W. (1992). A Note on the Delta Method. Am Stat *46*, 27-29.

Reverter, M., Rentero, C., Garcia-Melero, A., Hoque, M., Vila de Muga, S., Alvarez-Guaita, A., Conway, J.R., Wood, P., Cairns, R., Lykopoulou, L.*, et al.* (2014). Cholesterol regulates Syntaxin 6 trafficking at trans-Golgi network endosomal boundaries. Cell Rep *7*, 883-897.

Samuel, M.S., Lopez, J.I., McGhee, E.J., Croft, D.R., Strachan, D., Timpson, P., Munro, J., Schroder, E., Zhou, J., Brunton, V.G.*, et al.* (2011). Actomyosin-mediated cellular tension drives increased tissue stiffness and beta-catenin activation to induce epidermal hyperplasia and tumor growth. Cancer cell *19*, 776-791.

Samuel, M.S., Munro, J., Bryson, S., Forrow, S., Stevenson, D., and Olson, M.F. (2009). Tissue selective expression of conditionally-regulated ROCK by gene targeting to a defined locus. Genesis *47*, 440-446.

Schachtner, H., Li, A., Stevenson, D., Calaminus, S.D., Thomas, S.G., Watson, S.P., Sixt, M., Wedlich-Soldner, R., Strathdee, D., and Machesky, L.M. (2012). Tissue inducible Lifeact expression allows visualization of actin dynamics in vivo and ex vivo. European journal of cell biology *91*, 923-929.

Serebriiskii, I., Castello-Cros, R., Lamb, A., Golemis, E.A., and Cukierman, E. (2008). Fibroblast-derived 3D matrix differentially regulates the growth and drug-responsiveness of human cancer cells. Matrix biology : journal of the International Society for Matrix Biology *27*, 573-585.

Srinivas, S., Watanabe, T., Lin, C.S., William, C.M., Tanabe, Y., Jessell, T.M., and Costantini, F. (2001). Cre reporter strains produced by targeted insertion of EYFP and ECFP into the ROSA26 locus. BMC developmental biology *1*, 4.

Thevenaz, P., Ruttimann, U.E., and Unser, M. (1998). A pyramid approach to subpixel registration based on intensity. IEEE Trans Image Process *7*, 27-41.
Tucker, K.L., Wang, Y., Dausman, J., and Jaenisch, R. (1997). A transgenic mouse strain expressing four drug-selectable marker genes. Nucleic Acids Res *25*, 3745-3746.
