# Supplementary figures and images for: Intravital FRAP Imaging using an E-cadherin-GFP Mouse Reveals Disease- and Drug-Dependent Dynamic Regulation of Cell-Cell Junctions in Live Tissue
Source: Cell Rep. 2015 Dec 24;14(1):152–67. doi: 10.1016/j.celrep.2015.12.020 (PMC4709331; doi:10.1016/j.celrep.2015.12.020)

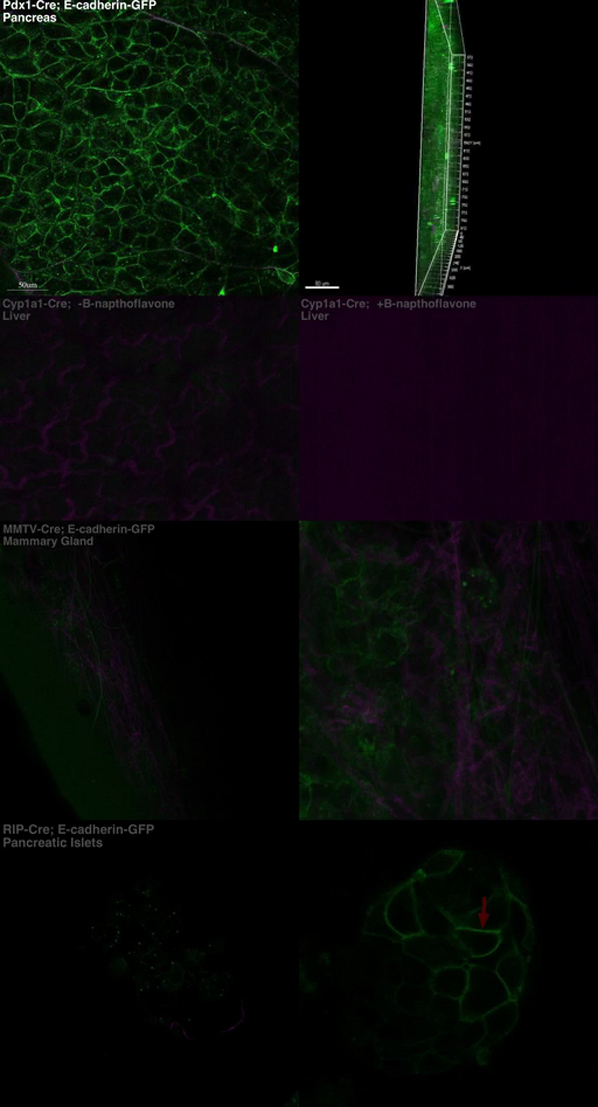

Supplement: Movie S1. Tissue-Specific and Induced Expression of E-cadherin-GFP, Related to Figure 1 [file mmc2.jpg]

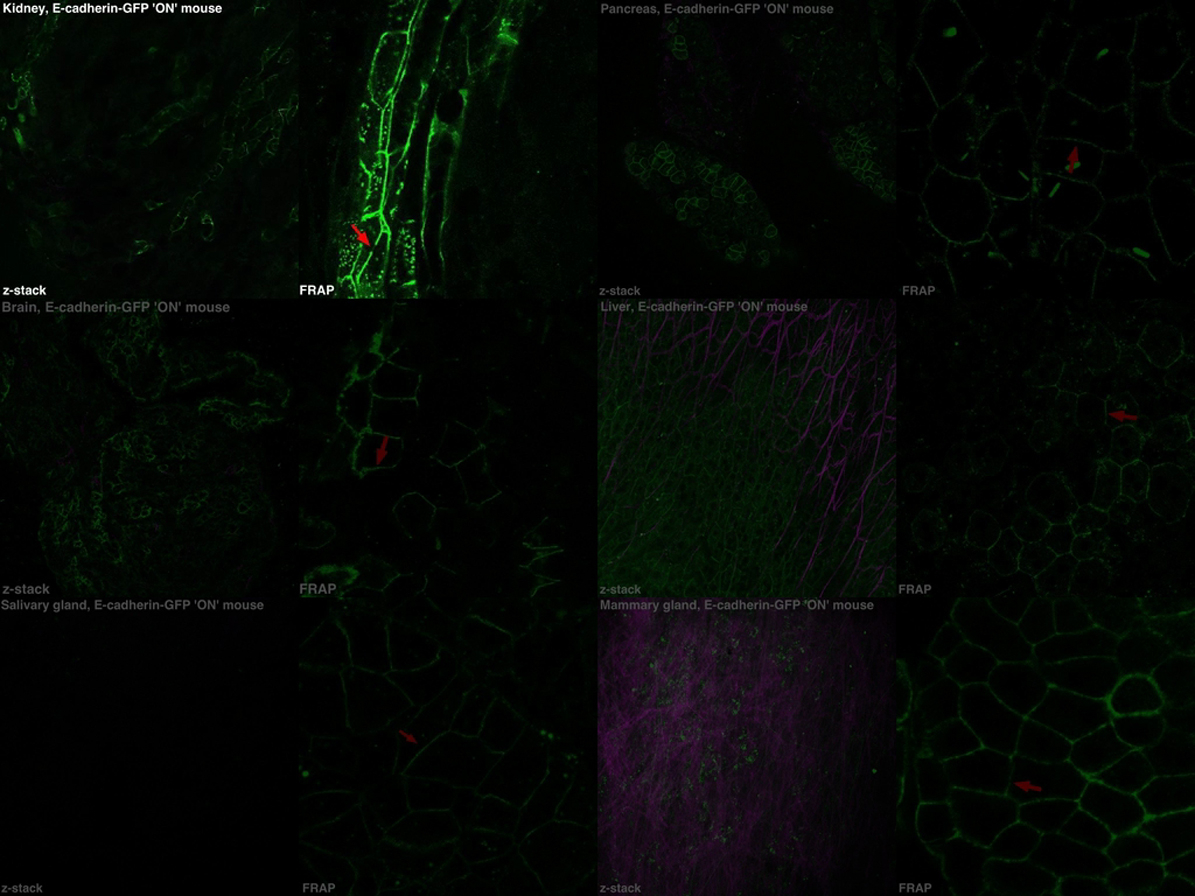

Supplement: Movie S2. Constitutive Expression of E-cadherin-GFP, Related to Figure 1 [file mmc3.jpg]

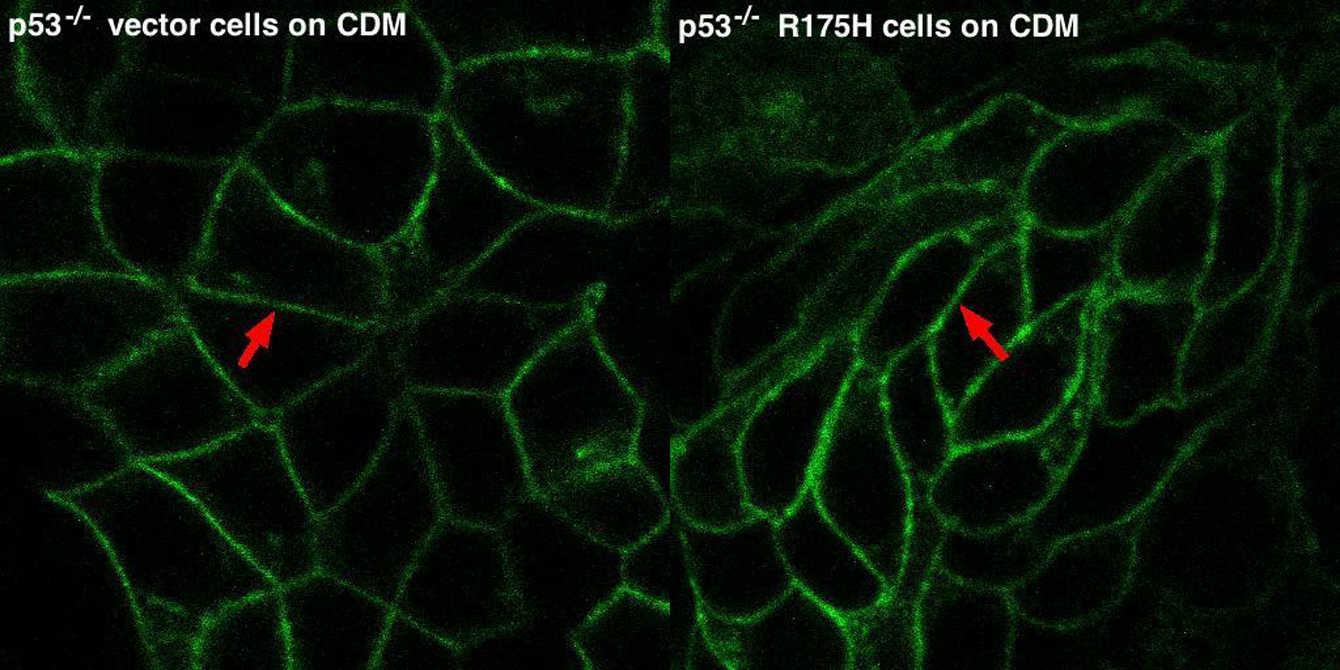

Supplement: Movie S3. FRAP on CDMs, Related to Figure 2 [file mmc4.jpg]

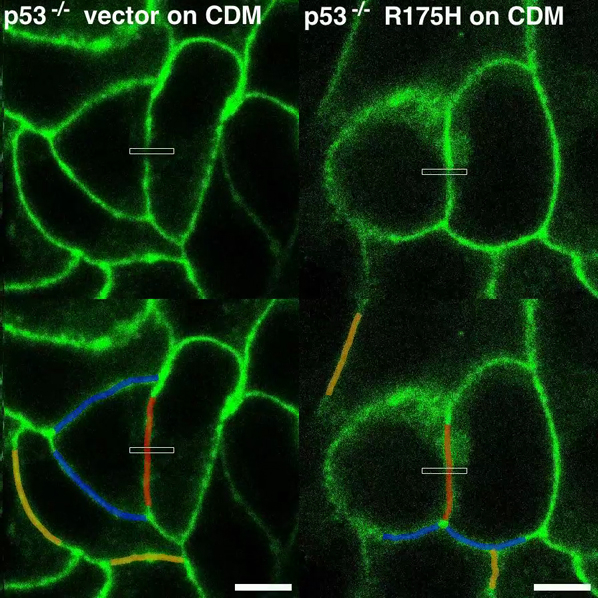

Supplement: Movie S4. FLIP on CDMs, Related to Figure 2 [file mmc5.jpg]

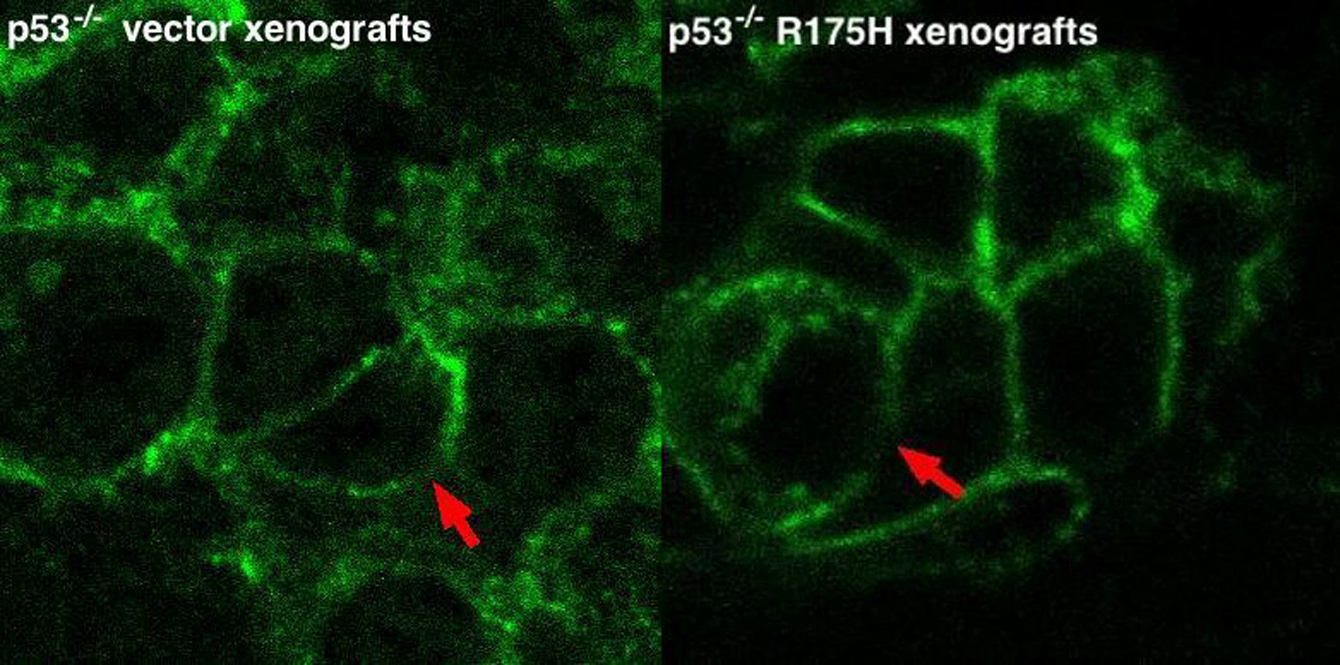

Supplement: Movie S5. FRAP in Xenografts, Related to Figure 3 [file mmc6.jpg]

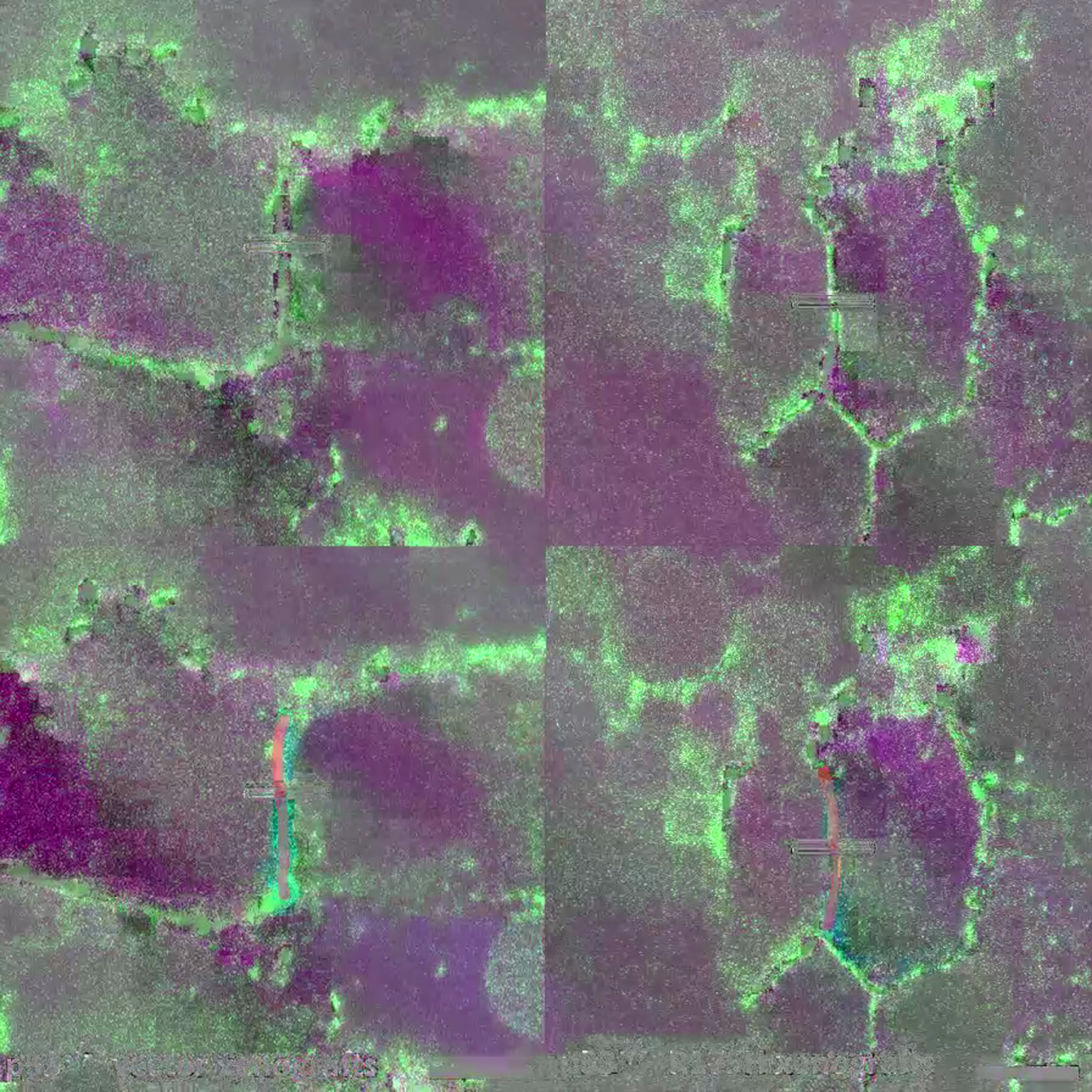

Supplement: Movie S6. FLIP on Xenografts, Related to Figure 3 [file mmc7.jpg]

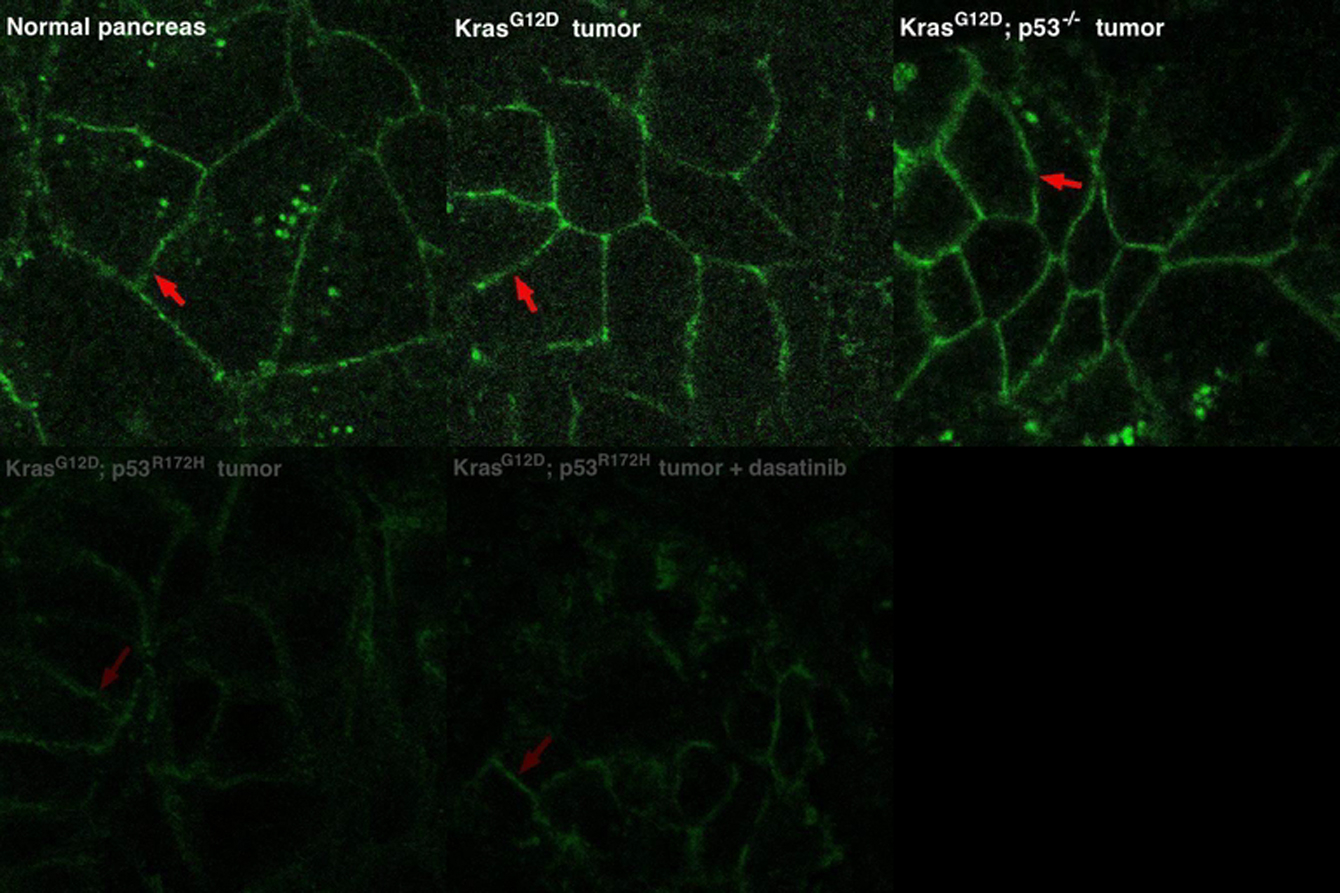

Supplement: Movie S7. FRAP in Genetically Engineered Mouse Models of PDAC Formation and following Drug Treatment, Related to Figures 5 and 6 [file mmc8.jpg]
