# Supplementary material for: Intravital FRAP Imaging using an E-cadherin-GFP Mouse Reveals Disease- and Drug-Dependent Dynamic Regulation of Cell-Cell Junctions in Live Tissue
Source: Cell Rep. 2015 Dec 24;14(1):152–67. doi: 10.1016/j.celrep.2015.12.020 (PMC4709331; doi:10.1016/j.celrep.2015.12.020)
Supplement: Document S2. Article plus Supplemental Information [file mmc9.pdf]

Cell Reports

Resource

Intravital FRAP Imaging using an E-cadherin-GFP Mouse Reveals Disease- and Drug-Dependent Dynamic Regulation of Cell-Cell Junctions in Live Tissue

Graphical Abstract

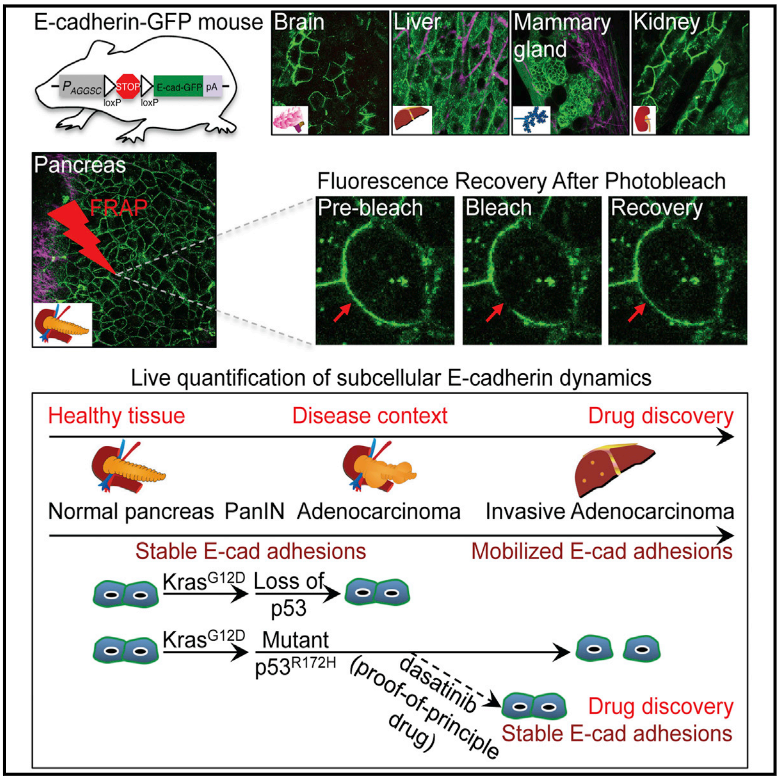

Highlights

- The E-cadherin-GFP mouse allows in situ quantification of E-cadherin mobility
- We monitored E-cadherin mobility during tissue homeostasis and disease development
- Invasive pancreatic cancer driven by mutant Kras/p53 increases E-cadherin mobility
- Dasatinib treatment reverts E-cadherin mobility and reinforces tumor cell junctions

Authors

Zahra Erami, David Herrmann,
Sean C. Warren, ..., Jennifer P. Morton,
Kurt I. Anderson, Paul Timpson

Correspondence

k.anderson@beatson.gla.ac.uk (K.I.A.),
p.timpson@garvan.org.au (P.T.)

In Brief

Erami et al. generate an E-cadherin-GFP mouse to demonstrate real-time quantification of E-cadherin mobility using intravital photobleaching in a range of tissue types. They show that changes in E-cadherin mobility correlate with changes in cell junction integrity and invasiveness while demonstrating applications of the mouse for future drug discovery studies.

CrossMark
Erami et al., 2016, Cell Reports *14*, 152–167
January 5, 2016 ©2016 The Authors
http://dx.doi.org/10.1016/j.celrep.2015.12.020

CellPress

OPEN ACCESS
CellPress

Cell Reports
Resource

Intravital FRAP Imaging using an E-cadherin-GFP Mouse Reveals Disease- and Drug-Dependent Dynamic Regulation of Cell-Cell Junctions in Live Tissue

Zahra Erami,[1,5] David Herrmann,[2,5] Sean C. Warren,[2] Max Nobis,[1] Ewan J. McGhee,[1] Morghan C. Lucas,[2] Wilfred Leung,[2] Nadine Reischmann,[2] Agata Mrowinska,[1] Juliane P. Schwarz,[1] Shereen Kadir,[1] James R.W. Conway,[2] Claire Vennin,[2] Saadia A. Karim,[1] Andrew D. Campbell,[1] David Gallego-Ortega,[2] Astrid Magenau,[2] Kendelle J. Murphy,[2] Rachel A. Ridgway,[1] Andrew M. Law,[2] Stacey N. Walters,[2] Shane T. Grey,[2] David R. Croucher,[2] Lei Zhang,[2] Herbert Herzog,[2] Edna C. Hardeman,[3] Peter W. Gunning,[4] Christopher J. Ormandy,[2] T.R. Jeffry Evans,[1] Douglas Strathdee,[1] Owen J. Sansom,[1] Jennifer P. Morton,[1] Kurt I. Anderson,[1,6,*] and Paul Timpson[2,6,*]
[1]Cancer Research UK Beatson Institute, Switchback Road, Bearsden, Glasgow G61 1BD, UK
[2]The Garvan Institute of Medical Research and The Kinghorn Cancer Centre, Cancer Division, St Vincent's Clinical School, Faculty of Medicine, University of New South Wales, Sydney, NSW 2010, Australia
[3]Neuromuscular and Regenerative Medicine Unit, School of Medical Sciences, University of New South Wales, Sydney, NSW 2052, Australia
[4]Oncology Research Unit, School of Medical Sciences, University of New South Wales, Sydney, NSW 2052, Australia
[5]Co-first author
[6]Co-senior author
*Correspondence: k.anderson@beatson.gla.ac.uk (K.I.A.), p.timpson@garvan.org.au (P.T.)
http://dx.doi.org/10.1016/j.celrep.2015.12.020
This is an open access article under the CC BY license (http://creativecommons.org/licenses/by/4.0/).

SUMMARY

E-cadherin-mediated cell-cell junctions play a prominent role in maintaining the epithelial architecture. The disruption or deregulation of these adhesions in cancer can lead to the collapse of tumor epithelia that precedes invasion and subsequent metastasis. Here we generated an E-cadherin-GFP mouse that enables intravital photobleaching and quantification of E-cadherin mobility in live tissue without affecting normal biology. We demonstrate the broad applications of this mouse by examining E-cadherin regulation in multiple tissues, including mammary, brain, liver, and kidney tissue, while specifically monitoring E-cadherin mobility during disease progression in the pancreas. We assess E-cadherin stability in native pancreatic tissue upon genetic manipulation involving Kras and p53 or in response to anti-invasive drug treatment and gain insights into the dynamic remodeling of E-cadherin during in situ cancer progression. FRAP in the E-cadherin-GFP mouse, therefore, promises to be a valuable tool to fundamentally expand our understanding of E-cadherin-mediated events in native microenvironments.

INTRODUCTION

The capacity of cancer cells to dissociate from primary tumors and invade requires the deregulation of interactions with adjacent cells and the surrounding tissue. A major challenge in biology is the real-time monitoring of protein dynamics involved in this process in their native context (Conway et al., 2014). The ability to quantify the intricate spatiotemporal regulation of cell adhesion molecules, such as E-cadherin, using techniques including fluorescence recovery after photobleaching (FRAP) has rapidly enhanced our understanding of E-cadherin's subcellular roles in regulating cell-cell integrity and dissociation in vitro (Canel et al., 2010a; Shen et al., 2008; Sprague and McNally, 2005; Wu et al., 2014).

FRAP is commonly used for monitoring molecular movement within cells. A small fluorescent region is bleached, and fluorescence recovery into the bleached region is measured over time (Axelrod et al., 1976; Fritzsche and Charras, 2015; Sprague and McNally, 2005). From this, multiple readouts can be derived, including, but not limited to the half-time of recovery, a measure of the rate at which fluorescent molecules move in or out of the region of interest, and the immobile fraction, an indication of how much of the molecule remains trapped and unable to move out of the analyzed region (for in-depth insights into FRAP analysis, see Fritzsche and Charras, 2015). In the case of fluorescently labeled E-cadherin, the immobile fraction can indicate how much E-cadherin is trapped or engaged in cell-cell junctions and may provide a molecular readout of junction stability in real time (Canel et al., 2010b; Serrels et al., 2009).

FRAP has largely been used to probe molecular events within 2D cell culture using transfection-based approaches (Lippincott-Schwartz et al., 2001; Shen et al., 2008), whereas its utility in vivo has been limited (Ellenbroek and van Rheenen, 2014). Recently, we and others have used FRAP in more complex and physiologically relevant environments ranging from application in *Drosophila* (Cavey et al., 2008) to the use of E-cadherin-GFP FRAP in a mammalian system in vivo (Serrels

OPEN ACCESS
CellPress

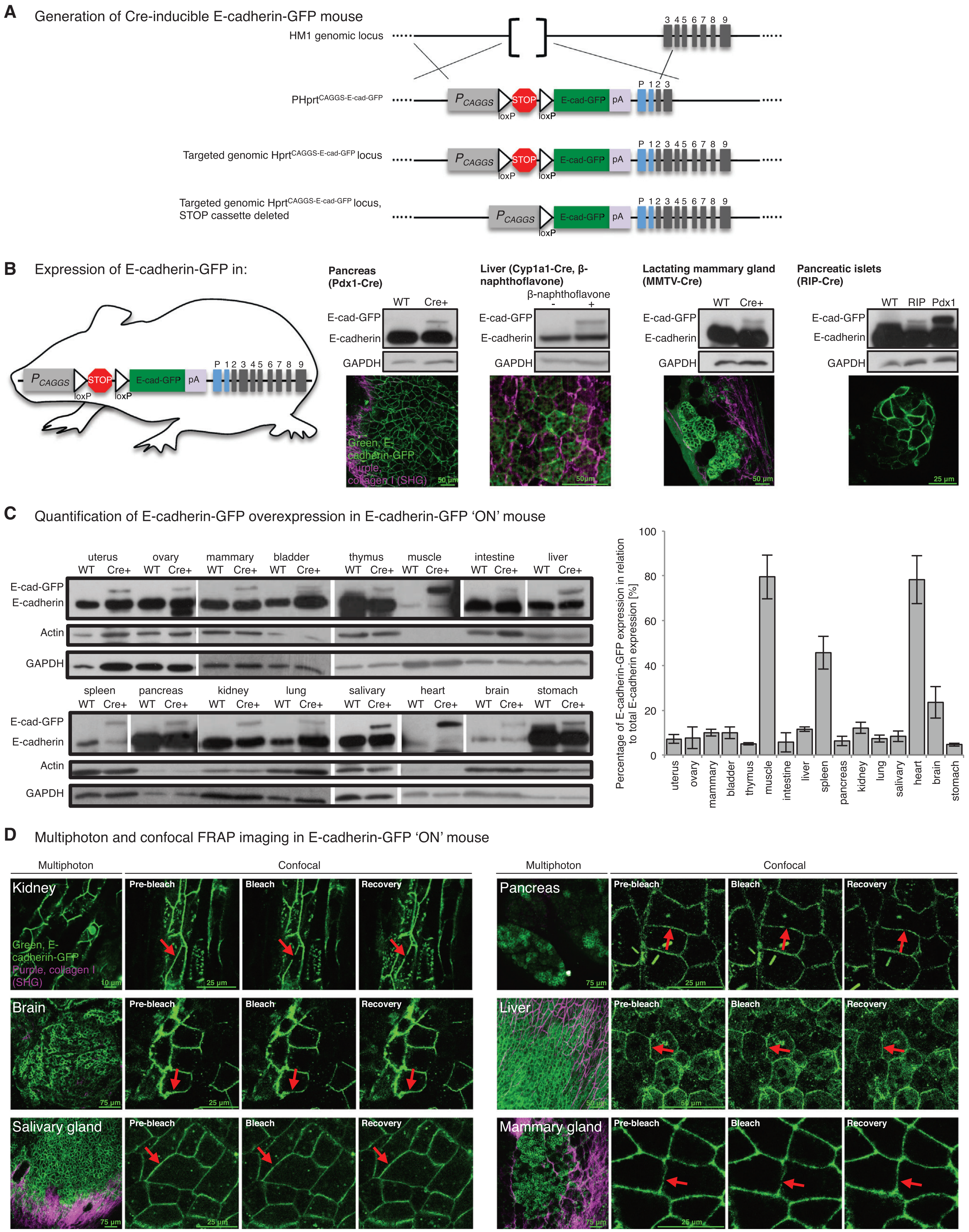

(legend on next page)

et al., 2009), where E-cadherin-GFP-expressing squamous cell carcinoma cells were transplanted and grown subcutaneously in mice. Using this approach, we demonstrated that locally invading cells had a significantly lower immobile fraction of E-cadherin-GFP compared with non-invading cells, illustrating that, in cancer cells that retain E-cadherin expression, mobilization rather than loss of E-cadherin can play a role in reducing cell-cell adhesions, leading to more malleable, motile, and invasive tumor behavior (Friedl et al., 2012; Lamouille et al., 2014; Shamir et al., 2014). Although these approaches provide insights into the mobilization of E-cadherin in subcutaneous xenograft tumors, they lack the fidelity to recapitulate the complex and distinct microenvironment found in specific organs of interest (Conway et al., 2014). It is therefore essential to develop new tools for the quantification of molecular dynamics in distinct organs in which the disease of interest originates, allowing us to understand cell behavior at a subcellular and tissue- and disease-specific level.

In this study, we generated a Cre-inducible E-cadherin-GFP mouse and exploited this model using intravital FRAP imaging to assess changes in E-cadherin-based cell-cell junction integrity during disease progression and in response to drug treatment. Here we mimic the disease etiology of pancreatic cancer in situ, from acquisition of an initiating Kras mutation that occurs in 95% of pancreatic cancers (Biankin et al., 2012; Hingorani et al., 2003) to subsequent loss- or gain-of-function mutations in the tumor suppressor p53, which occur in 50%–75% of pancreatic cancers (Biankin et al., 2012). Using FRAP analysis, we reveal that E-cadherin stability in normal pancreas or in non-invasive pancreatic tumors ($Kras^{G12D}$ alone or $Kras^{G12D}$; $p53^{-/-}$) remains unaltered during disease progression. However, in mice with $Kras^{G12D}$ and gain-of-function mutations in p53 ($p53^{R172H}$), E-cadherin is mobilized, facilitating the weakening of cell-cell contacts, correlating with the enhanced metastasis seen in this model (Hingorani et al., 2005; Morton et al., 2010b). Moreover, in line with recent work assessing Src kinase as an anti-invasive drug target (Avizienyte et al., 2002; Morton et al., 2010a; Nobis et al., 2013, 2014), we demonstrate that the phase II drug dasatinib reverts E-cadherin mobilization in invasive pancreatic tumors and that this stabilization of junctions could partially explain its current anti-invasive role in this disease (T.J. Evans et al., 2012, ASCO, abstract). We therefore present the application of FRAP in the E-cadherin-GFP mouse for live, tissue-specific assessment of vital cell-cell adhesion changes in conjunction with its utility as a pre-clinical imaging tool for evaluating the efficacy of new therapeutics in the pancreas and other organs of interest in real time.

RESULTS

Generation of E-cadherin-GFP Mice for In Vivo FRAP Assessment

Conditional E-cadherin-GFP-expressing mice were generated by targeting a lox-stop-lox transgene under the control of a CAG promoter into the deleted *Hprt* locus of HM1 embryonic stem (ES) cells (Figure 1A; Bronson et al., 1996). We first generated E-cadherin-GFP "OFF" mice, a strain in which expression of the extensively validated EGFP-linked E-cadherin fusion protein (Lock and Stow, 2005) was conditionally prevented by a transcriptional stop sequence (Figure 1A). To demonstrate the utility and scope of the E-cadherin-GFP mouse for examining the regulation of E-cadherin in different organs, we crossed E-cadherin-GFP OFF mice to tissue-specific Cre strains to induce organ- and cell-type-specific expression in the pancreas (the focus of this study), liver, mammary gland, and pancreatic islets (Figure 1B). Expression of E-cadherin-GFP in the pancreas via Pdx1-Cre could be imaged readily at depth (Movie S1, first panel; green, E-cadherin-GFP; purple, second harmonic generation [SHG] imaging of the extracellular matrix [ECM]). In the liver, E-cadherin-GFP OFF mice were crossed to Cyp1a1-Cre mice (Ireland et al., 2004), and deletion of the stop cassette was achieved after three doses of 2 mg β-naphthoflavone over 8 hr (Movie S1, second panel). Robust expression in mammary tissue via MMTV-Cre (Wagner et al., 1997) was imaged in the alveoli of lactating mice where junction integrity is required during milk production (Movie S1, third panel; Figures S1A and S1B), whereas sub-organ specificity within islet cells of the pancreas via RIP-Cre (Postic et al., 1999) could be demonstrated in isolated pancreatic islets (Movie S1, fourth panel).

Conditional E-cadherin-GFP mice were subsequently crossed to mice expressing CMV Cre recombinase (Schwenk et al., 1995) to enable constitutive, low-level expression of E-cadherin-GFP in every tissue (Figure 1C). Analysis of E-cadherin in multiple tissue types from the E-cadherin-GFP "ON" mouse illustrates the broader capacity for subcellular FRAP imaging in normal or disease contexts of interest (Figure 1D; Movie S2, left panels, kidney, brain and salivary gland; right panels, pancreas, liver, and mammary tissue). Importantly, mice of both E-cadherin-GFP strains were born at the expected Mendelian ratio, were fertile and healthy, and exhibited no untoward behaviors. In this study, we demonstrate the application of photobleaching in the E-cadherin-GFP mouse as a method for assessing the subcellular regulation of E-cadherin in the progression of invasive pancreatic cancer. However, this system could be used in other disease states in future investigations.

Figure 1. Generation of Conditional and Constitutive E-cadherin-GFP Mice

(A) Schematic of the generation of a conditional E-cadherin-GFP OFF strain by targeting a lox-stop-lox transgene under the control of a CAG promoter to the *Hprt* locus.

(B) Conditional E-cadherin-GFP expression in pancreas (Pdx1-Cre), liver (Cyp1a1-Cre), mammary gland (MMTV-Cre), and pancreatic islets (RIP-Cre). Shown are western Blot analyses in wild-type (WT) and transgenic organs (top band, E-cadherin-GFP; bottom band, endogenous E-cadherin) and corresponding multiphoton images.

(C and D) Western Blot analyses in 16 organs (C) and multiphoton images of E-cadherin GFP expression (D, column 1) and confocal FRAP (D, columns 2–4) in six organs of the E-cadherin-GFP ON mouse. E-cadherin-GFP, green; SHG signal, purple; red arrow, bleach point.

See also Figure S1.

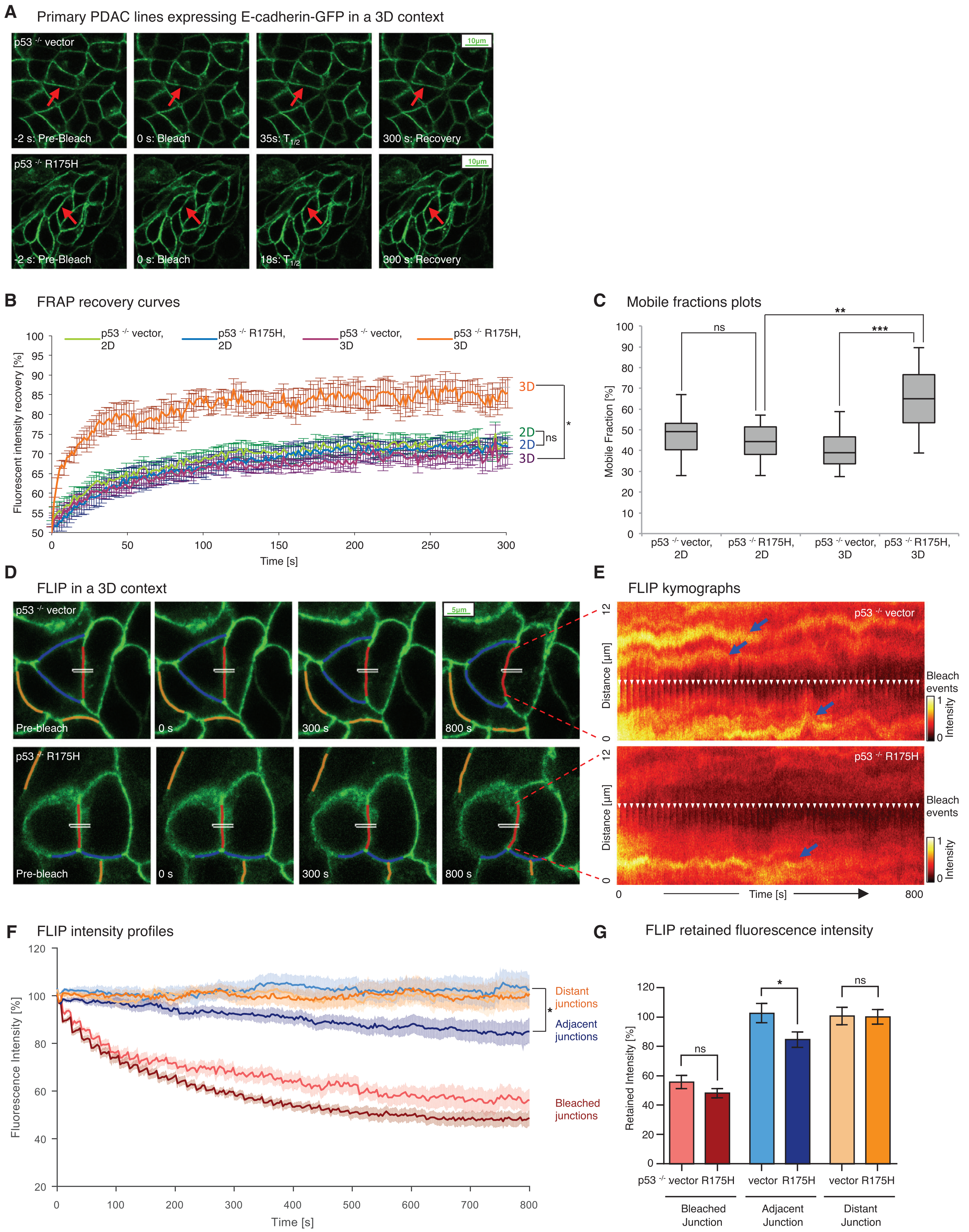

(legend on next page)

FRAP Assessment of E-cadherin in Two versus Three Dimensions Reveals Mobilization Differences between Non-invasive and Invasive Pancreatic Cancer

Prior to generation of the E-cadherin-GFP mouse, we first established primary non-invasive pancreatic tumor cell lines from Pdx1-Cre; Kras$^{G12D/+}$; p53$^{flox/+}$ mice (Morton et al., 2010b) in which the wild-type p53 allele is lost (hereafter referred to as p53$^{-/-}$). Using these cells, we generated stable pancreatic ductal adenocarcinoma (PDAC) p53$^{-/-}$ cells expressing empty vector alone (p53$^{-/-}$ vector) or the human equivalent of murine p53^{R172H} (p53$^{-/-}$ R175H) with E-cadherin-GFP (Muller et al., 2013; Tan et al., 2014). The resulting invasive mutant p53$^{-/-}$ R175H and non-invasive p53$^{-/-}$ vector cells expressing E-cadherin-GFP (Figure 2A; Movie S3) serve as a fluorescent model system to gauge the initial spatiotemporal regulation of E-cadherin in pancreatic cancer (Morton et al., 2010b; Muller et al., 2013; Tan et al., 2014) without affecting fundamental processes such as cell proliferation or invasive capacity (Figures S1C and S1D).

FRAP recovery curves from time-lapse movies of p53$^{-/-}$ vector or p53$^{-/-}$ R175H primary PDAC cells were then analyzed (Canel et al., 2010a, 2010b; Serrels et al., 2009; Vlahov et al., 2015; Supplemental Experimental Procedures; Figure S2). Assessment in two dimensions revealed no difference in E-cadherin mobility of invasive p53$^{-/-}$ R175H versus non-invasive p53$^{-/-}$ vector cells (Figure 2B, blue recovery curve versus green), where the fraction of E-cadherin-GFP stabilized in cell-cell junctions was similar in both cell types (Figure 2C, mobile fraction 45.6% ± 2.7% for p53$^{-/-}$ vector compared with 46% ± 3.1% for p53$^{-/-}$ R175H cells).

Critically, assessment in a 3D environment using cells grown on cell-derived matrices (CDMs) to mimic in vivo conditions (Herrmann et al., 2014; Yamada and Cukierman, 2007) revealed enhanced mobilization of E-cadherin in invasive cells, where mutant p53$^{-/-}$ R175H cells had a significantly higher mobile fraction of E-cadherin compared with p53$^{-/-}$ vector cells (Figures 2B, orange recovery curve versus purple, and2C, average mobile fraction 39.9% ± 2.8% versus 65.6% ± 4.4%; Figure S3A). This is in line with the early cell-cell dissociation preceding cell scattering and invasion we observed recently in mutant p53 cells compared with p53$^{-/-}$ cells (Muller et al., 2013; Tan et al., 2014) and highlights the need to assess E-cadherin cell-cell adhesion events in a 3D context.

To further assess E-cadherin mobility and corroborate our findings in this 3D setting, complementary fluorescence loss in photobleaching (FLIP) analysis was performed on p53$^{-/-}$ vector versus mutant p53$^{-/-}$ R175H cells grown on CDMs (Figures 2D–2G; Sakurai-Yageta et al., 2015). Here, by continually bleaching an area within a cell-cell junction, we observed that, in p53$^{-/-}$ vector cells, junctions adjacent to the bleached region did not significantly reduce fluorescence intensity over the time course of our FLIP analysis after correcting for imaging-induced photobleaching (Figures 2D and 2E; Movie S4 [the white rectangle indicates a repeated bleach region, and red, blue, and orange lines represent bleached, adjacent, and distant non-related control junctions, respectively]). Here, FLIP traces of adjacent junctions (Figures 2F and 2G, light blue line and bar graph) show no significant reduction in intensity over the 800-s time course. In contrast, mutant p53$^{-/-}$ R175H cells exhibited a significant loss of fluorescence intensity in junctions adjacent to the bleach region over time (Figures 2F and 2G, dark blue line and bar graph), commensurate with the transfer of bleached E-cadherin molecules into this area and an enhanced mobilization of E-cadherin junctions in mutant p53 cells compared with p53$^{-/-}$ vector cells.

To assess whether E-cadherin is distributed equally at distinct points of the membrane in PDAC cells, we created kymographs of unbleached junctions from p53$^{-/-}$ vector and p53$^{-/-}$ R175H cells (Figures S3B–S3D). We observed an elevated distribution of high-intensity zones of E-cadherin-GFP in p53$^{-/-}$ vector cells compared with p53$^{-/-}$ R175H cells (Figures S3C and S3D, blue arrows). These zones persisted over the time course of imaging and could represent stable E-cadherin zones, as identified previously by others (Adams et al., 1998; Cavey et al., 2008). To quantify the uniformity or texture of this E-cadherin intensity across the membrane, we used orientation-dependent grey level co-occurrence matrix (OD-GLCM) analysis of the kymographs (Figure S3B; Hu et al., 2012; Nobis et al., 2013). This analysis revealed that the inherent distribution of high-intensity E-cadherin-GFP zones was significantly different between p53$^{-/-}$ vector and p53$^{-/-}$ R175H junctions (quantified as contrast in Figure S3E), indicating a larger number of zones of high E-cadherin intensity in non-invasive p53$^{-/-}$ vector cells. Collectively, these data support our initial FRAP results, revealing a critical mobilization of E-cadherin in mutant p53 cells in 3D in vitro settings.

PDAC cells were therefore injected into CD1 nude mice and allowed to form subcutaneous tumors. In line with previous work (Muller et al., 2013; Timpson et al., 2011), p53$^{-/-}$ R175H tumor-bearing mice demonstrated collective local invasion compared with encapsulated non-invasive p53$^{-/-}$ vector tumors (Figure 3A). FRAP recovery curves from time-lapse movies of xenograft tumors obtained using intravital FRAP imaging (Figure 3B) revealed an enhanced mobile fraction in p53$^{-/-}$ R175H versus p53$^{-/-}$ vector cells in this setting (Figures 3C and 3D,

Figure 2. E-cadherin Junctions Are Mobilized in Three Dimensions in Invasive PDAC Cells

(A) Representative images from confocal FRAP movies of non-invasive p53$^{-/-}$ vector (top) versus invasive p53$^{-/-}$ R175H cells (bottom) grown on CDM. Arrows, bleached regions.

(B and C) FRAP recovery curves (B) and mobile fraction plots (C) comparing E-cadherin-GFP mobilization between p53$^{-/-}$ vector and p53$^{-/-}$ R175H cells grown in a 2D versus 3D environment. n = 3, >10 junctions/group.

(D) Representative images from FLIP movies of p53$^{-/-}$ vector (top) and p53$^{-/-}$ R175H cells (bottom) grown on CDM. White rectangles, bleach regions; red lines, tracked bleached junctions; blue lines, tracked adjacent junctions; orange lines, distant control junctions.

(E) Photobleach-corrected kymographs showing the intensity profile along the bleached junction over time. White arrows, bleach events.

(F and G) Photobleach-corrected, normalized intensity profiles (F) and averaged fraction of fluorescence retained after 800 s (G) for bleached (red), adjacent (blue), and distant junctions (orange) of p53$^{-/-}$ vector (light colors, n = 13) and p53$^{-/-}$ R175H cells (dark colors, n = 14).

Columns, mean; bars, ± SE. *p < 0.05; **p < 0.01; ***p < 0.001. ns, not significant (unpaired Student's t test). See also Figures S2 and S3.

 ©2016 The Authors

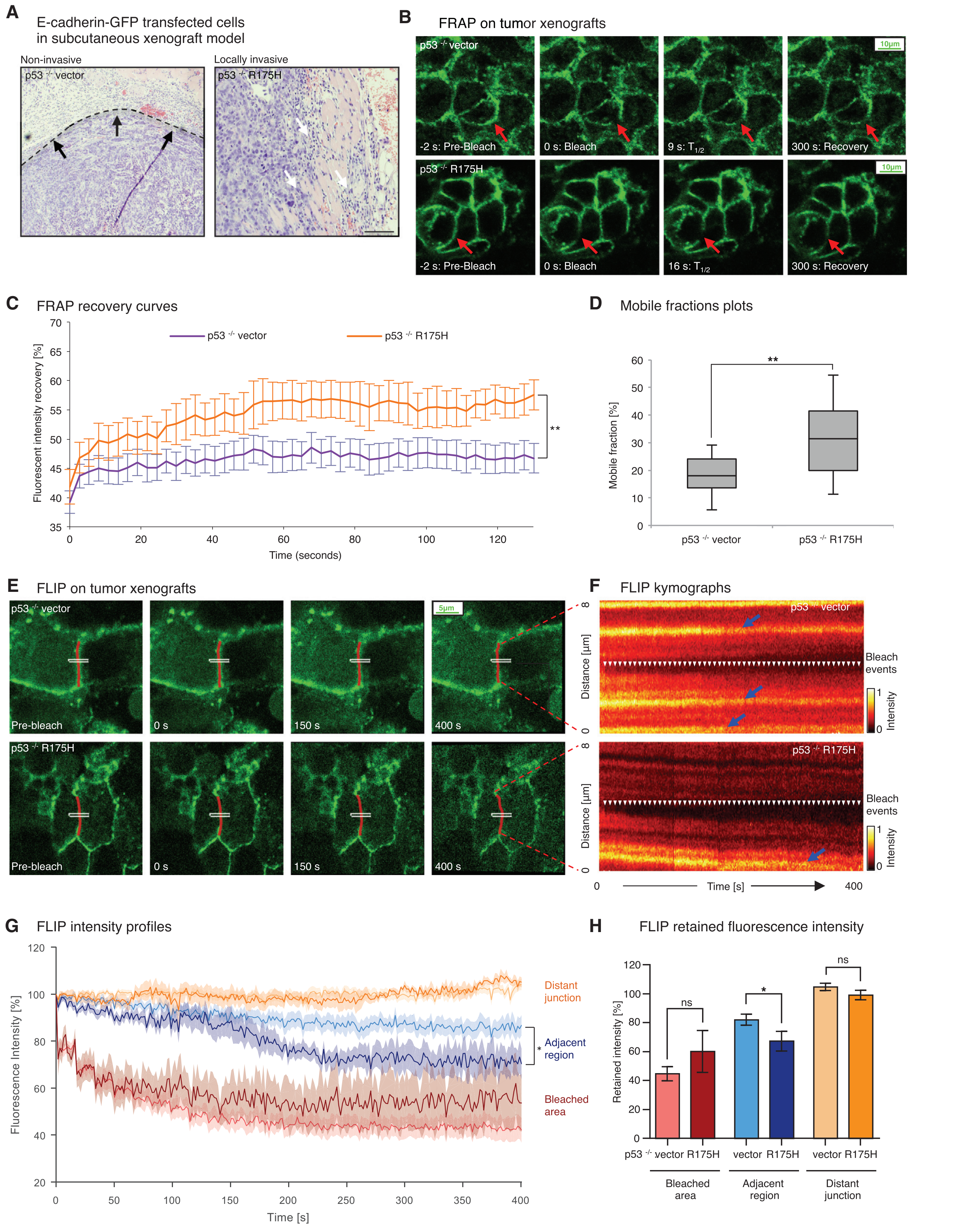

(legend on next page)

32.1% ± 2.9% versus 18.8% ± 1.7%; Figure S3F; Movie S5). Similarly, FLIP analysis of the xenograft tumors (Figures 3E–3H; Movie S6) shows a significantly higher loss of fluorescence in areas proximal to the bleach region in p53$^{-/-}$ R175H tumors compared with p53$^{-/-}$ vector tumors, corroborating our FRAP analysis (Figures 3G and 3H, compare light blue and dark blue FLIP traces and bar graphs). Furthermore, OD-GLCM analysis of unbleached junctions again showed a higher contrast of high versus low E-cadherin-GFP intensity/texture in p53$^{-/-}$ vector tumors compared with p53$^{-/-}$ R175H tumors (Figures S3G–S3I), indicating an increased stabilization of E-cadherin in p53$^{-/-}$ vector control cells in vivo.

To further investigate the differences in E-cadherin mobility between p53$^{-/-}$ vector and p53$^{-/-}$ R175H tumors, we bleached a large region of interest with a 3-μm diameter. The change in intensity profile along the membrane of a large FRAP bleach region over time can provide insights into the processes governing recovery (modeled in Figures 4A and 4B). We therefore generated kymographs of the bleached junction and measured the fluorescence intensity along the junction via line scan analysis at defined time points (Figures 4C and 4F, colored arrowheads; de Beco et al., 2012; Delva and Kowalczyk, 2009). We then fitted the results of our line scan analysis to a Gaussian curve model (Figures 4D and 4G) and plotted the decrease in width of the Gaussian curves over time as a measure of the speed of fluorescence recovery (Figures 4E and 4H). If the recovery was due to lateral movement in the membrane, then the width of the bleached region should increase linearly with time (Figure 4A). Conversely, if the recovery was due to cytoplasmic uniformed exchange, then the width of the bleached region should remain uniform with time (Figure 4B). Here we find that the width of the bleached region expands significantly faster in p53$^{-/-}$ R175H xenografts compared with p53$^{-/-}$ vector xenografts, indicating faster lateral motion of E-cadherin in the mutant xenografts (Figures 4C–4H; compare the slopes as a measure of the recovery speed between Figures 4E and 4H, with the effective diffusion coefficient D_{eff} = 4.88 ± 0.3 × 10^{-3} μm^2 s^{-1} versus 1.70 ± 0.1 × 10^{-3} μm^2 s^{-1}, respectively). This indicates that the increase in E-cadherin mobility observed in locally invasive p53$^{-/-}$ R175H xenografts may be primarily attributed to increased mobility within the membrane.

Although this subcutaneous model confirmed that enhanced mobilization of E-cadherin exists in live 3D tumor settings, it does not recapitulate the distinct microenvironment found in the pancreas. This prompted us to investigate whether subcellular mobilization of E-cadherin occurs in the organ of tumor origin and, if so, whether the stage of in situ disease progression in which these changes occur can be identified (Bardeesy and DePinho, 2002).

FRAP in the E-cadherin-GFP Mouse Reveals Genetically Driven Progression of Cell-Cell Junction Disruption in Pancreatic Tissue during In Situ Disease Development

Our ability to use FRAP in vivo led us to investigate whether we can assess E-cadherin mobility in live pancreatic tissue. The E-cadherin-GFP mouse was therefore crossed with either wild-type mice expressing Pdx1-Cre, mice bearing a Pdx1-Cre-driven initiating Kras^{G12D} mutation alone, Pdx1-Cre; Kras^{G12D}; p53$^{-/-}$ mice, or Pdx1-Cre; Kras^{G12D}; mutant p53^{R172H} mice and monitored for ~150 days to allow tumor formation to occur. This allowed us to recapitulate the genetic changes commonly occurring during the formation of invasive PDAC (Figures 5A–5C; Movie S7) in this extensively validated model of pancreatic cancer.

FRAP recovery curves from time-lapse movies of wild-type E-cadherin-GFP mice (Figure 5D, green line) were analyzed to gauge the inherent mobility of E-cadherin in normal pancreatic tissue (Figure 5E, average mobile fraction = 21.9% ± 1.6%; Figure S4A). Interestingly, the fraction of E-cadherin stabilized in cell-cell junctions in wild-type mice was similar to that of non-invasive tumors bearing Kras^{G12D} (Figures 5D and 5E, blue line, mobile fraction = 24.3% ± 2.3%). This indicates that initiating Kras^{G12D} mutations in the pancreas are not sufficient to drive E-cadherin mobilization in situ.

Importantly, loss of p53 on a Kras^{G12D} background from age-matched tumors also had no effect on E-cadherin mobility compared with normal pancreatic tissue or Kras^{G12D} tumors alone (Figures 5D and 5E, purple line, 21.6% ± 1.7% mobile fraction). This demonstrates that, although loss of the tumor suppressor p53 in combination with initiating Kras^{G12D} mutations enhances tumor progression, it does not act to mobilize E-cadherin, correlating with the lack of invasion or metastasis we observed previously in Kras^{G12D}; p53$^{-/-}$ mice (Morton et al., 2010b).

Critically, FRAP analysis of tumors from ~150-day-old, Kras^{G12D}; p53^{R172H}; E-cadherin-GFP mice revealed that gain-of-function mutations in p53 on a Kras^{G12D} background significantly mobilizes E-cadherin junctions in the pancreas (Figures 5D and 5E, orange line, 31.8% ± 1.8% mobile fraction compared with the average of 22.4% ± 1.1% found in normal pancreas,

Figure 3. FRAP and FLIP in Live Tumors Reveal E-cadherin Mobilization during Invasion

(A) Representative images of encapsulated p53$^{-/-}$ vector tumors (left, dashed line, black arrows) versus locally invasive p53$^{-/-}$ R175H tumors (right, white arrows).

(B) Representative images from confocal FRAP movies of p53$^{-/-}$ vector (top) versus p53$^{-/-}$ R175H xenografts (bottom). Red arrows, bleached regions.

(C and D) FRAP recovery curves (C) and mobile fraction plots (D) comparing E-cadherin-GFP mobilization between p53$^{-/-}$ vector and p53$^{-/-}$ R175H cells grown in a 2D versus 3D environment. n = 3, >10 junctions/group.

(E) Representative images from FLIP movies of p53$^{-/-}$ vector (top) and p53$^{-/-}$ R175H xenografts (bottom). White rectangles, bleach regions; red lines, tracked bleached junctions.

(F) Photobleach-corrected kymographs showing the intensity profile along the bleached junction over time. Arrows, bleach events.

(G and H) Photobleach-corrected, normalized intensity profiles (G) and averaged fraction of fluorescence retained after 400 s (H) for the bleached region (red), areas 3 μm distant from the bleached region (blue), and distant control junctions (orange) of p53$^{-/-}$ vector (light colors, n = 9) and p53$^{-/-}$ R175H cells (dark colors, n = 5).

Columns, mean; bars, ± SE; n = 3 mice; >5 cells/group. *p < 0.05; **p < 0.01 (unpaired Student's t test). See also Figures S2 and S3.

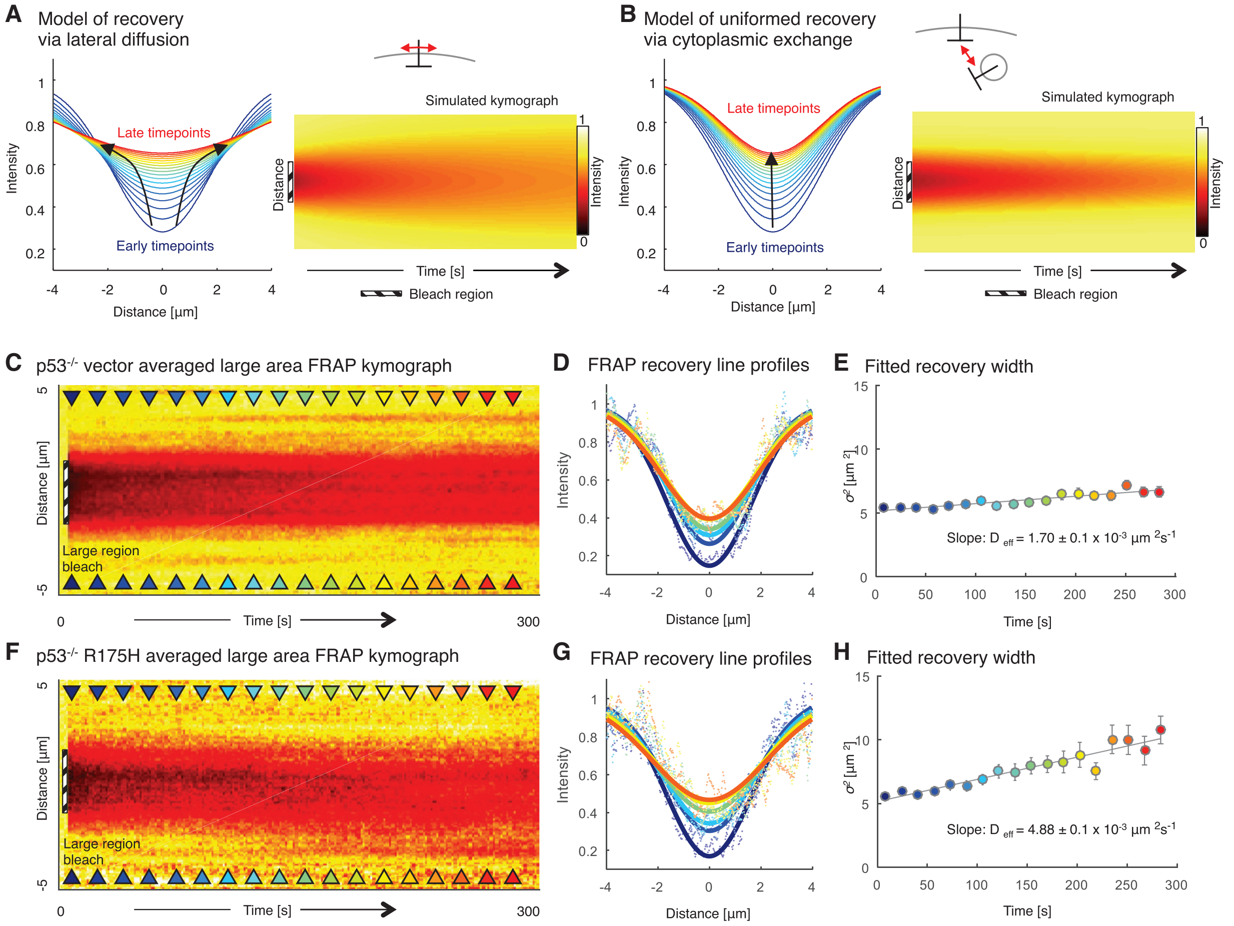

Figure 4. Large-Area E-cadherin-GFP FRAP in PDAC Xenografts

(A, B, C, and F) Models of recovery via lateral membrane diffusion (A) and via cytoplasmic exchange (B) with line profiles along the bleached region during recovery (early time points, blue; late time points, red) and simulated intensity kymographs. Black arrows highlight changes in recovery (C and F). Also shown is a normalized average kymograph for large-region FRAP of $p53^{-/-}$ vector (C, n = 9) and $p53^{-/-}$ R175H (F, n = 9) xenografts. Colored arrowheads indicate time points where profiles were fitted and shown in later plots (early time points, blue; late time points, red). Dashed black areas, bleached regions.

(D and G) Line profiles from recovery (early time points, blue; late time points, red) with fitted Gaussian curves (solid lines).

(E and H) Fitted width (σ^2) of Gaussian curves for selected time points, with linear fit (gray line) used to calculate the diffusion coefficient (slope). Error bars, confidence interval on fitted parameter.

$Kras^{G12D}$, or $Kras^{G12D}$; $p53^{-/-}$ tumors). This indicates that acquisition of mutant $p53^{R172H}$, which we have shown previously to drive an invasive and metastatic program over and above the loss of p53 in pancreatic cancer (Morton et al., 2010b), could partially achieve this by allowing early tumor dissolution via mobilization of E-cadherin-based cell-cell contacts (Figure 5F). Therefore, we sought to assess whether this potentially pro-invasive molecular event could be impaired pharmacologically and monitored in a pre-clinical setting using the E-cadherin-GFP mouse.

The E-cadherin-GFP Mouse as a Tool to Monitor Early Anti-invasive Drug Response in Real Time

Recently we demonstrated that Src kinase, which is known to induce the disruption of E-cadherin-based cell-cell junctions either directly (Avizienyte et al., 2002; Calautti et al., 1998; Frame et al., 2002) or indirectly via the stroma (Yang et al., 2010), plays a prominent role in invasive pancreatic cancer (Morton et al., 2010a). We therefore examined whether the mobilization and potential weakening of E-cadherin junctions observed in invasive pancreatic tumors of Pdx1-Cre; $Kras^{G12D}$; $p53^{R172H}$ E-cadherin-GFP mice could be reverted using dasatinib.

As before, Pdx1-Cre; $Kras^{G12D}$; $p53^{R172H}$; E-cadherin-GFP mice were allowed to form tumors for ~150 days and, upon signs of tumor burden, were placed on daily dasatinib treatment (10 mg/kg) for 3 consecutive days (Figure 6A), followed by FRAP analysis (Figure 6B; Movie S7). This revealed that reversion of E-cadherin mobilization back to the levels found in wild-type pancreas or non-invasive $Kras^{G12D}$; $p53^{-/-}$ tumors could be achieved using dasatinib treatment at this stage of the disease (Figures 6C and 6D, compare mobile fraction of $Kras^{G12D}$; $p53^{R172H}$ mice ± dasatinib, 18.1% ± 1.3% versus 31.8% ± 1.8%, and 5E, $Kras^{G12D}$ alone or $Kras^{G12D}$; $p53^{-/-}$, mobile fraction of 24.3% ± 2.3% and 21.6% ± 1.7%; Figure S4B).

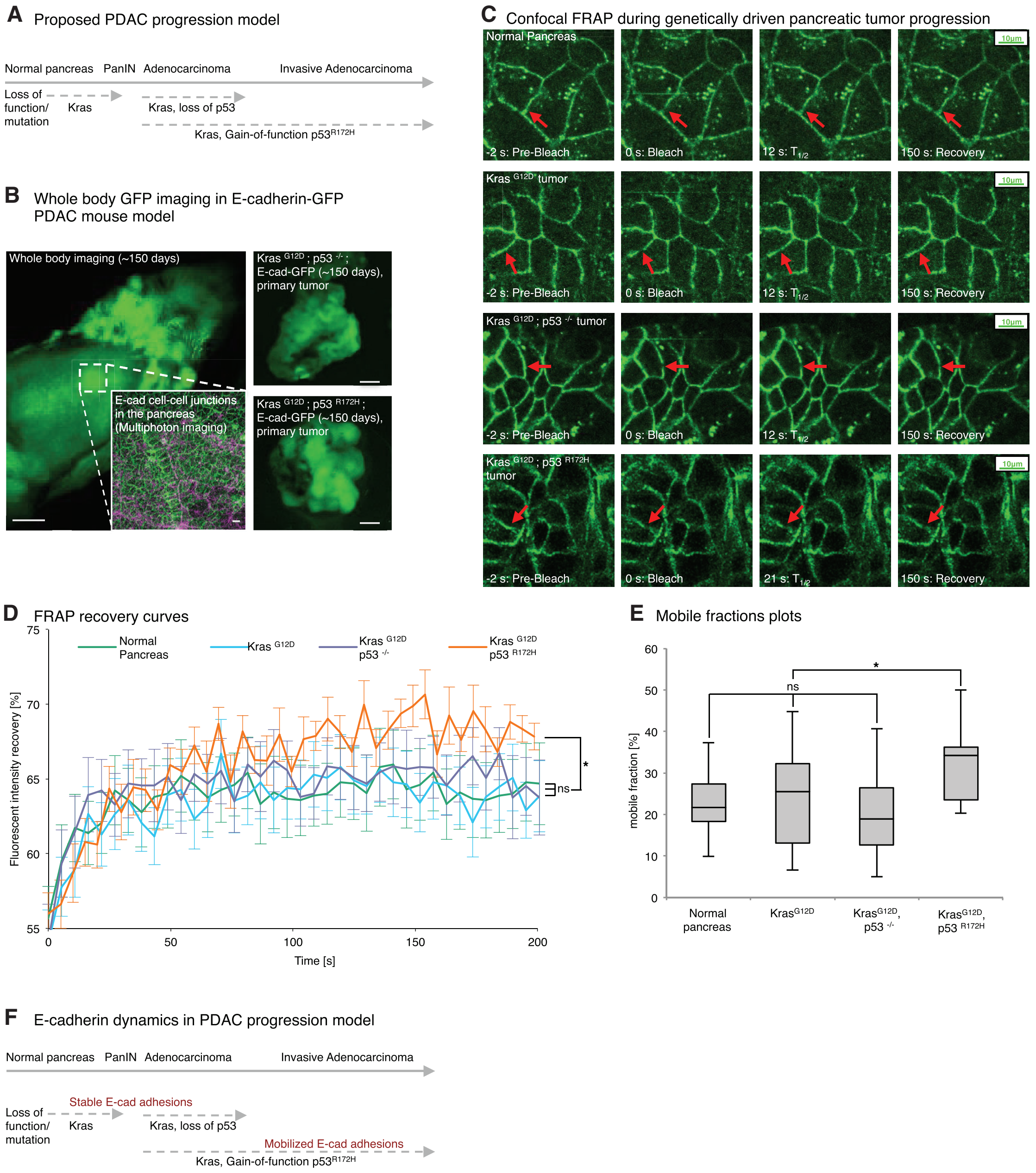

Figure 5. E-cadherin-GFP FRAP during Tissue Homeostasis, Initiation, and Progression of Pancreatic Cancer
(A) Proposed pancreatic cancer progression model from initiating Kras mutations (PanIN) to subsequent loss of p53 or gain-of-function mutations in p53.
(B) Representative whole-body image of an E-cadherin-GFP mouse with multiphoton-based cell-cell junction imaging (inset: E-cadherin-GFP, green; SHG signal, purple).
(C) Representative images from confocal FRAP movies in the E-cadherin-GFP mouse from normal pancreas (first row) to acquiring $Kras^{G12D}$ (second row) with subsequent loss of p53 (third row) or $p53^{R172H}$ gain-of function mutations (bottom row), respectively. Red arrows, bleached regions.
(D and E) Graphs comparing E-cadherin-GFP mobilization between in situ pancreatic tissue from normal (green), $Kras^{G12D}$ alone (blue), $Kras^{G12D}$; $p53^{-/-}$ (purple), and $Kras^{G12D}$; $p53^{R172H}$ (orange) mice. Columns, mean; bars, ± SE, n = 3 mice/group, ≥21 junctions in total. *$p < 0.05$ (unpaired Student's t test).
(F) Proposed schematic of E-cadherin dynamics during PDAC progression.
See also Figure S4.

These results indicate that the recently demonstrated efficacy of dasatinib in this model could partly be due to its capacity to stabilize E-cadherin junctions and prevent dissociation from the primary tumor. We therefore sought to determine whether the E-cadherin-GFP FRAP readouts we observed in this study could be correlated with changes in junction integrity and strength.

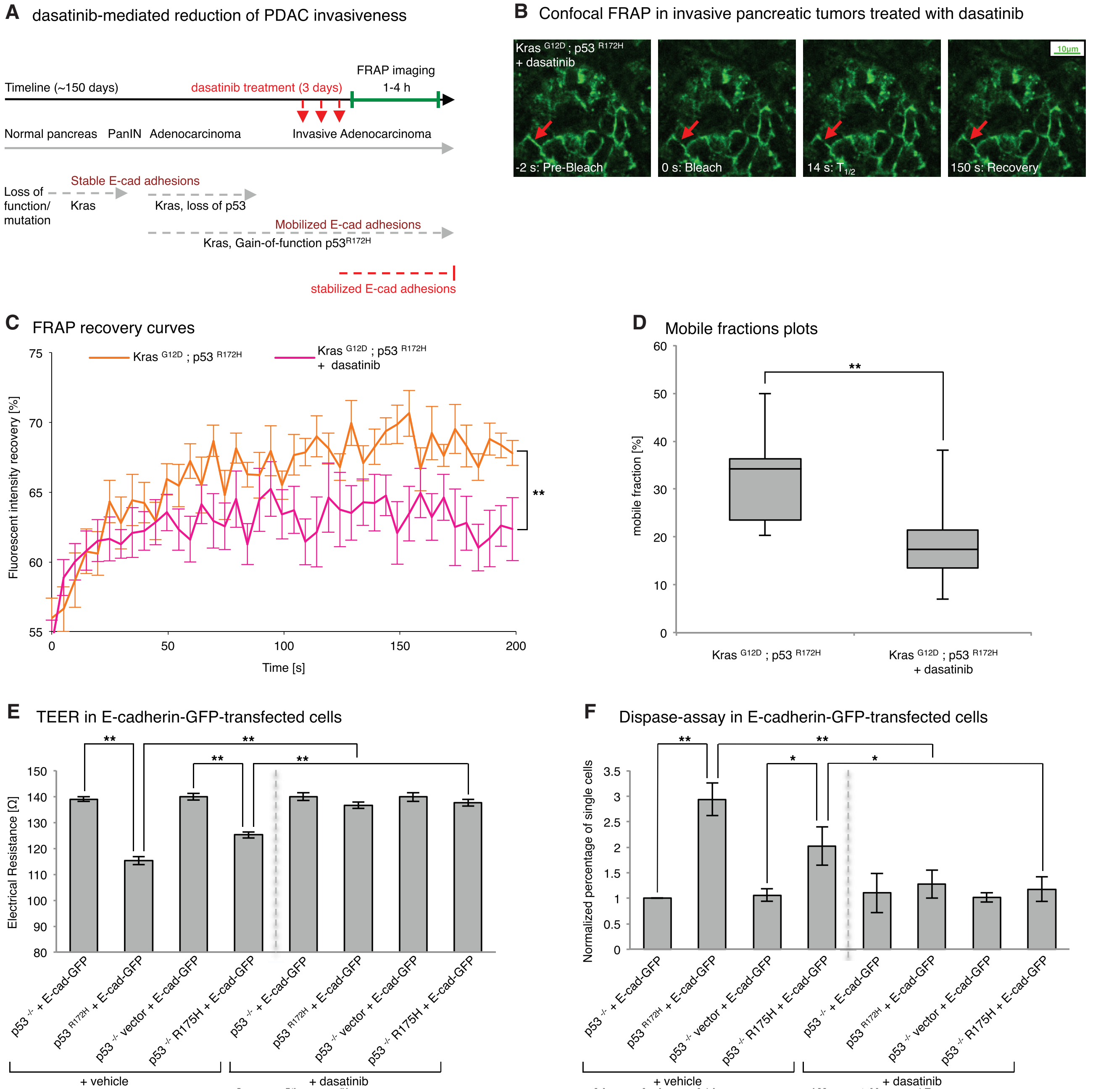

Figure 6. The E-cadherin-GFP Mouse as a Tool to Monitor the Early Anti-invasive Drug Response

(A) Schematic of anti-invasive dasatinib treatment using Kras^{G12D}; p53^{R172H}; E-cadherin-GFP mice.

(B) Representative images from confocal FRAP movies of dasatinib-treated Kras^{G12D}; p53^{R172H}; E-cadherin-GFP mouse (oral gavage, 3× daily, 10 mg/kg). Red arrows, bleached regions.

(C and D) Graphs comparing E-cadherin-GFP mobilization in in situ pancreatic tissue of control (orange) and dasatinib-treated (pink) Kras^{G12D}; p53^{R172H}; E-cadherin-GFP mice. Columns, mean; bars, ± SE; n = 3 mice/group, ≥21 junctions in total. **$p < 0.01$ (unpaired Student's t test).

(E and F) TEER (E) and Dispase assays (F) in E-cadherin-GFP-transfected p53$^{-/-}$ PDAC cells (columns 1 and 3, pre-dasatinib; columns 5 and 7, post-dasatinib) versus mutant p53 PDAC cells (columns 2 and 4, pre-dasatinib; columns 6 and 8, post-dasatinib). Columns, mean; bars, ± SE; n = 3 mice/group. *$p < 0.05$; **$p < 0.01$ (unpaired Student's t test).

See also Figure S4.

Using complementary assays, we interrogated the integrity and strength of cell-cell junctions using trans-endothelial electrical resistance (TEER) and Dispase II assays, respectively (Calautti et al., 1998; Canel et al., 2010b; von Bonsdorff et al., 1985). TEER allows a rapid evaluation of junction integrity as a function of electrical resistance between cells where high resistance correlates with high junction integrity (von Bonsdorff et al., 1985). Examination of the electrical resistance between non-invasive $p53^{-/-}$ and invasive $p53^{R172H}$ cells transfected with E-cadherin-GFP demonstrated that junction integrity was significantly lower in a mutant $p53^{R172H}$ background than in a $p53^{-/-}$ setting (Figure 6E, columns 1 and 2). Similar results were obtained in E-cadherin-GFP-transfected $p53^{-/-}$ R175H and $p53^{-/-}$ vector cells (Figure 6E, columns 3 and 4), confirming that mutant p53 reduces junction integrity in PDAC cells.

In line with our results demonstrating that dasatinib impairs invasion via potentially enhancing cell-cell adhesions, TEER analysis of dasatinib-treated mutant p53-expressing PDAC cells (E-cadherin-GFP-transfected $p53^{R172H}$ or $p53^{-/-}$ R175H cells) revealed that Src inhibition (Figures S4D and S4E) stabilized and strengthened junctions to a similar level as that found in a non-invasive $p53^{-/-}$ setting (Figure 6E, columns 5–8). This was confirmed using the Dispase II assay, where mutant p53 cells (E-cadherin-GFP-transfected $p53^{R172H}$ or $p53^{-/-}$ R175H cells) show an increased number of single cells upon mechanical disruption compared with $p53^{-/-}$ E-cadherin-GFP-transfected counterparts (Figure 6F, columns 1–4). Importantly, cell dissociation was reverted to similar levels as those found in non-invasive $p53^{-/-}$ or vector-alone counterparts when mutant p53 cells were treated with dasatinib (Figure 6F, compare columns 1–4 with columns 5–8). These results illustrate that mutant p53 on an initiating $Kras^{G12D}$ background weakens cell-cell junction strength, which could lead to tumor dissociation, and that Src inhibition can partially impair this breakdown. Importantly, we corroborated these finding with another Src inhibitor, saracatenib (Figures S4D and S4E; Nam et al., 2013), and observed a similar strengthening and enhancement of junction integrity via TEER and Dispase II analysis (Figures S4F and S4G), with a corresponding stabilization of E-cadherin mobility, as assessed by FRAP on CDMs (Figure S4C). These data, therefore, demonstrate that imaging subtle changes in the mobility of proteins involved in maintaining normal epithelial architecture, such as E-cadherin, can provide a surrogate marker of dissociation events that may precede the collapse of cell-cell adhesions, correlating with tumor cell invasion.

Monitoring E-cadherin Dynamics in Secondary Metastases

In line with the concept of stabilizing E-cadherin junctions via Src inhibition, we isolated metastatic tumor cells that had colonized the liver of invasive Pdx1-Cre; $Kras^{G12D}$; $p53^{R172H}$; E-cadherin-GFP mice using whole-body fluorescence imaging (Figures 7A and 7B, whole-body imaging and inset, respectively, showing primary tumor and liver micro-metastases isolated from the same mouse). We confirmed that metastatic cells from the E-cadherin-GFP mouse retained similar E-cadherin expression levels as cell lines obtained from primary tumors, and similar levels of proliferation (Figures 7C–7E, Figures S5A and S5B), while maintaining the capacity to invade in 3D organotypic matrices (Figures 7F, 7G, and S5C, left). Importantly, upon treatment of these invasive cells with dasatinib, invasion was reduced significantly compared with control cells (Figures 7F, 7G, and S5C, right, for three independent metastatic lines from three independent mice [101912 met, 101025 met, and 111375 met, respectively]). Moreover, the integrity of cell-cell contacts in all lines was partially restored under dasatinib-treated conditions, leading to enhanced cell-cell clustering (Figures 7F, 7G, and S5C). Subcellular immunofluorescent staining of E-cadherin showed improved cell clustering in cells treated with dasatinib (Figures 7H, 7I, and S5D, red arrows). Importantly, we observed a similar reduction in invasion and a restoration in junction integrity for all three metastatic cell lines treated with saracatenib in organotypic assays (Figure S6A–S6E). Collectively, these data indicate that Src inhibition may partially stabilize E-cadherin-based cell-cell junctions and could retard early pancreatic tumor cell dissociation and, subsequently, decrease invasive efficiency by inducing tumor cell clustering.

This led us to examine whether the enhanced mobilization of E-cadherin and weakening of junction strength observed in invasive mutant p53 pancreatic tumors (Figures 3 and 5) could also be partly reverted in these more aggressive metastatic lines from the E-cadherin-GFP mouse. As before, TEER and Dispase II assays were carried out, and, in all three metastatic lines, a significant enhancement in junction integrity and strength was observed following dasatinib treatment (Figures 7J, 7K, and S5E). Again, similar effects on TEER and Dispase II analysis were observed upon saracatenib treatment in all three metastatic cell lines (Figures S6F–S6H). In contrast, dasatinib and saracatenib treatment had no effect on junction integrity and strength in primary tumor cells isolated from Pdx1-Cre; $Kras^{G12D}$; $p53^{-/-}$; E-cadherin-GFP mice (Figures S5F and S6I), which inherently possess high junction strength and integrity. Lastly, in line with our CDM and xenograft data (Figures 2 and 3), OD-GLCM analysis of mutant metastatic lines also revealed a reduction in contrast and a more uniform, malleable organization of E-cadherin-GFP distribution compared with primary tumor cells isolated from Pdx1-Cre; $Kras^{G12D}$; $p53^{-/-}$; E-cadherin-GFP mice (Figures S6J and S6K), suggesting that E-cadherin destabilization is maintained in mutant p53 metastatic cells derived from the E-cadherin-GFP mouse. Collectively, these results highlight the utility of the E-cadherin-GFP mouse as a pre-clinical imaging tool for monitoring early subcellular molecular changes that correlate with more aggressive tumor behavior.

DISCUSSION

Downregulation or loss of E-cadherin expression occurs in a number of cancer types and is thought to play a key role in epithelial-to-mesenchymal transition (EMT) associated with invasive cancer phenotypes (Gregory et al., 2008; Lamouille et al., 2014). However, in many invasive cancers, including pancreatic cancer, E-cadherin expression is often retained (David and Rajasekaran, 2012; Shamir et al., 2014) and utilized positively during cancer progression (Campbell and Casanova,

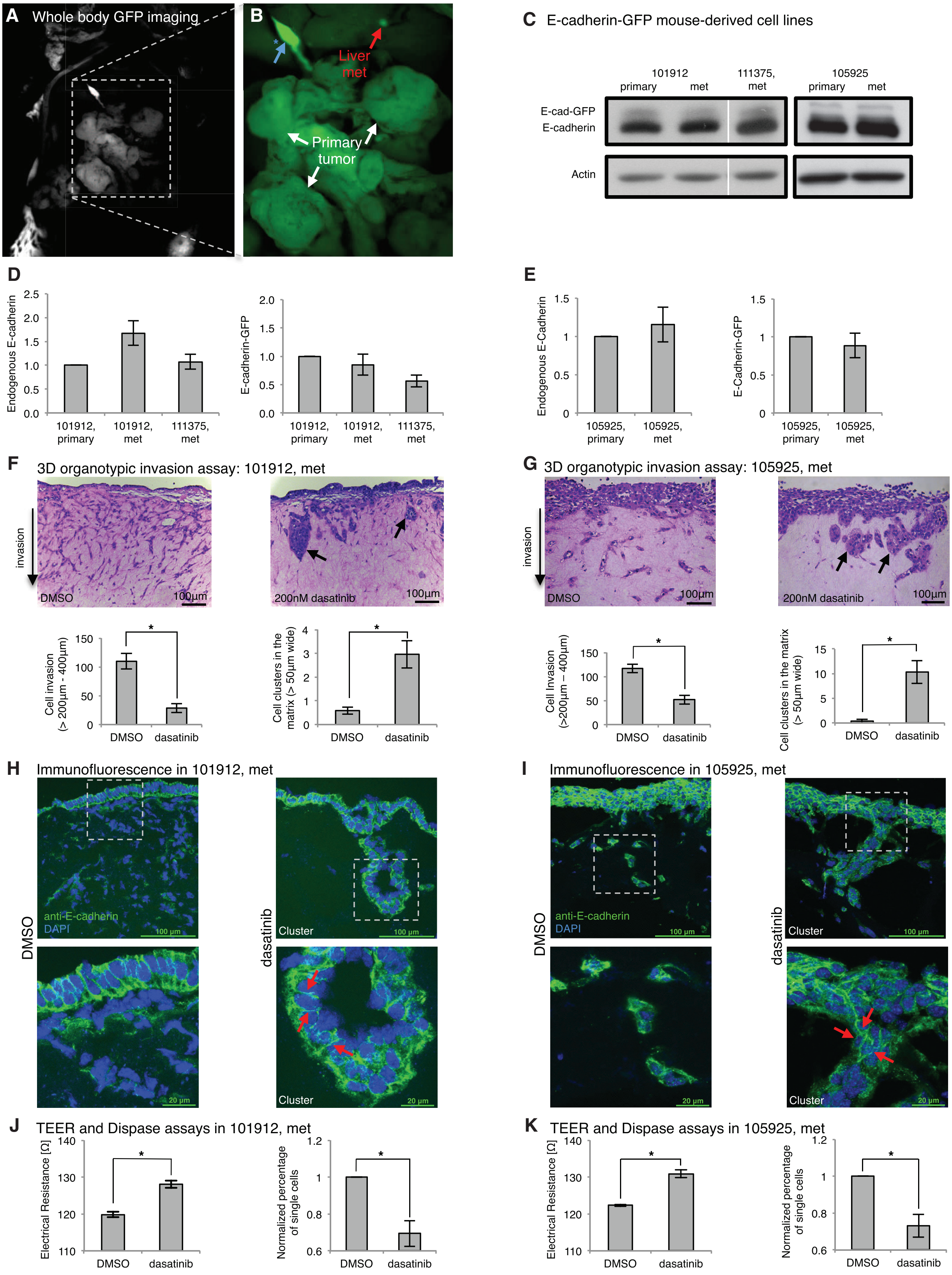

(legend on next page)

2015; Wicki et al., 2006). This indicates that, in many cases, deregulation of E-cadherin mobility rather than expression level may play an additional role in promoting invasion and suggests that monitoring E-cadherin behavior in this context could lead to a greater understanding of cancer invasion and response to new therapeutics (Wicki et al., 2006). Here, we present the generation of an E-cadherin-GFP mouse in which E-cadherin behavior can be monitored within intact tissues, such as the pancreas, liver, brain, kidney, and mammary tissue, via FRAP. Using this mouse in the context of pancreatic cancer, we reveal significant biological insights into the in situ regulation of E-cadherin during normal tissue homeostasis, disease initiation, and progression of pancreatic cancer while highlighting its potential utility for monitoring drug target activity in vivo using FRAP.

We chose the *Hprt* locus to drive E-Cadherin-GFP expression to provide the best signal-to-noise ratio for intravital imaging while aiming to minimize disturbance to early developmental events, such as tissue sorting (Halbleib and Nelson, 2006), and normal tissue function. Our assessment of the E-cadherin-GFP mouse, in the context of the comprehensively characterized *Kras*$^{G12D/+}$; *Trp53*$^{R172H/+}$; *Pdx1-Cre* (KPC) mouse model of pancreatic cancer, suggests that we gauged expression levels well, providing enough fluorescence for detection of pre- and post-photobleaching events without affecting primary tumor growth and metastasis. This is in line with our recent development of the Rac-Förster resonance energy transfer (FRET) biosensor mouse in which low-level expression driven via the *ROSA26* locus provided a sufficient signal for in vivo FRET imaging during disease progression without affecting the biology of the cells (Johnsson et al., 2014).

Crossing the E-cadherin-GFP mouse with distinct genetic models of pancreatic cancer has demonstrated its utility for examining the effects of specific mutations on E-cadherin in a time- and tissue-specific and subcellular manner. In pancreatic cancer, initiating mutations in the *KRAS* gene occur in approximately 95% of patients and are often followed by sequential accumulation of genetic alterations, such as loss of expression, function, or mutations in tumor suppressors and regulators, such as *TP53*, *CDKN2A*, *INK4A/ARF*, and/or *PTEN* (Bardeesy and DePinho, 2002; Kennedy et al., 2011; Ling et al., 2012; Morran et al., 2014). Although we know the frequency of such mutations in PDAC (Biankin et al., 2012), their specific function during development of invasive pancreatic cancer remains unclear. Here, we show that acquiring Kras^{G12D} mutations or subsequent loss of p53 on a Kras^{G12D} background in the pancreas is not sufficient to drive alterations to E-cadherin mobility, whereas acquisition of a gain-of-function mutation in p53 on the same initiating Kras^{G12D} background mobilizes E-cadherin, consistent with the tumor dissolution and metastasis found in these mice.

Importantly, reduction or reversion of cell-cell junction strength was achieved by molecular manipulation in the form of overexpressing the human equivalent of murine p53^{R172H} (R175H) or inhibiting E-cadherin mobilization via Src inactivation, respectively. This demonstrates that mutant p53 can partially drive the breakdown of tumors by disrupting E-cadherin cell-cell engagement via Src. It should be noted that both dasatinib and saracatenib, while inhibiting Src kinase, also target Abl and other kinases (Haubeiss et al., 2010) and were used here in a proof-of-principle context to demonstrate the utility of the mouse in pre-clinical drug discovery applications. To this end, the E-cadherin-GFP mouse could also be used to decipher other molecular pathways downstream of mutant p53 to be targeted in future studies or be used with other Kras or p53 mutations to determine whether E-cadherin mobilization is a general phenomenon for all mutant p53 variants in this disease (Muller et al., 2009, 2013; Muller and Vousden, 2014).

It is important to note that, in this study, we examined E-cadherin-GFP mobility throughout the entire pancreatic tumor tissue. However, heterogeneity is a hallmark of in vivo cancer, and it will be possible, in the future, to map areas of weakened cell-cell adhesions or drug response in the KPC model by examining E-cadherin mobilization in the center versus the invasive front of the tumor (Serrels et al., 2009). Similarly, the drug response in cancer cells in relation to local blood or drug supply could be assessed, as we and others have described previously (Manning et al., 2013; Nobis et al., 2013). In this way, we could determine, in the future, whether spatially regulated environmental cues in the native pancreas drive this heterogeneity or reflect a gradient in anti-invasive drug response in vivo.

Finally, the intricate control of in vivo distribution, function, as well as potential redundancy of EMT regulators such as Snail, Slug, Zeb, or Twist (Brabletz et al., 2013) could be investigated in pancreatic or other cancer types using the E-cadherin-GFP mouse. This could help dissect, in real time, the complex changes in E-cadherin mobility that occur during EMT, which, in many cases, does not involve loss of E-cadherin expression but rather a more complex dynamic regulation and utilization of E-cadherin function (David and Rajasekaran, 2012; Shamir et al., 2014; Wu et al., 2015).

The location, signaling, and dynamics of E-cadherin in the context of the surrounding environment can play a vital role

Figure 7. Monitoring E-cadherin Dynamics in Secondary Metastases from the E-cadherin-GFP Mouse

(A and B) Whole-body image of primary tumor (A) (B, white arrows) and isolated liver micro-metastases of a Kras$^{G12D/+}$; p53^{R172H}; E-cadherin-GFP mouse (B, red arrow). Blue arrow, auto-fluorescence in the bile duct.

(C–E) Western blot (C, top band, E-cadherin-GFP; bottom band, endogenous E-cadherin) and quantification (D and E) in isolated the primary and metastatic cell lines 101912 and 111375 (D) and 105925 (E).

(F and G) The isolated metastatic cell lines 101912 met (F) and 105925 met (G) from the liver of the E-cadherin-GFP mouse invading in organotypic matrices ± dasatinib (13 days, 200 nM). Black arrows, cluster formation upon dasatinib treatment. Shown is the quantification of cell invasion between 200–400 μm and cell clustering within matrices ± dasatinib.

(H and I) Immunofluorescence staining of E-cadherin within matrices ± dasatinib for the metastatic lines 101912 met (H) and 105925 met (I). Red arrows, junctions within cell clusters upon dasatinib treatment. E-cadherin, green; DAPI, blue.

(J and K) TEER (left) and Dispase assays (right) in metastatic lines derived from transgenic mouse, 101912 met (J), and 105925 met (K) ± dasatinib.

Columns, mean; bars, ± SE; n = 4 mice/group. *$p < 0.05$ (unpaired Student's t test). See also Figures S5 and S6.

 ©2016 The Authors

in disease progression. Our capacity to isolate and image micrometastases in the liver illustrates the power of this model for assessing subcellular changes in E-cadherin dynamics at secondary tumor sites that retain E-cadherin expression and may take advantage of this existing pool of E-cadherin during the early stages of mesenchymal-to-epithelial transition (MET) and tumor colonization (Lamouille et al., 2014; Ocaña et al., 2012). EMT-to-MET switching during extravasation and colonization is thought to be controlled precisely and has significant implications on whether anti-invasive drug targeting will be effective in impairing subsequent tumor colonization of the primary tumor in secondary sites (Brabletz et al., 2013). Preventing niche localization at the early stage of colonization by using anti-migratory drug targeting has been shown recently to be an effective strategy in impairing metastasis formation in the liver (Ritsma et al., 2012). However, understanding when and where EMT versus MET are detrimental or conducive to tumor extravasation will be of great interest in the future (Brabletz et al., 2013). Therefore, FRAP in the E-cadherin-GFP mouse could help us to understand this largely unexplored area of the metastatic cascade in a spatiotemporal and pre-clinical manner by allowing us to follow E-cadherin dynamics in both primary and secondary sites in the same mouse.

We focus on the application of FRAP in this study. However, other imaging techniques such as FLIP or anisotropy imaging of homo-FRET could also be employed in the future using the E-cadherin-GFP mouse to address more fundamental questions regarding E-cadherin regulation at distinct points in the membrane in situ (Levitt et al., 2015; Roberti et al., 2011). Furthermore, for fine-tuned assessment of E-cadherin via the endogenous E-cadherin promoter, engineering of a knockin E-cadherin-GFP mouse is currently underway as a next-generation mouse. Therefore, subtle changes to both E-cadherin levels and mobility could be monitored simultaneously during disease progression in cancers that also modulate E-cadherin expression. This could avoid any potential caveats or off-target effects caused by wrong lineage expression and could circumvent any minor changes to physiology that may have gone undetected in our model. Endogenous knockin strategies have been employed recently to examine the intricate dynamics of other cell-cell junction types in situ via the recent generation of ZO-1-GFP mice (Foote et al., 2013). Here, in line with our current work on adherens junctions, the authors report fundamental differences in tight junction dynamics in epidermal tissue in vivo, which cannot be recapitulated using 2D culture as a surrogate for living tissue. However, others have shown that E-cadherin can be differentially regulated within distinct points on the membrane, which could potentially be associated with zona adherens crosstalk (Priya et al., 2013).

In conclusion, FRAP in the E-cadherin-GFP mouse provides a flexible approach to monitor the intricate control of E-cadherin junctions in distinct biological processes in a time-, location-, and signaling-specific context. Future applications of this mouse as a tool alone or in combination with deficiencies of E-cadherin regulators and other tight junction components will profoundly expand our insights into the networks that govern E-cadherin responses in developmental, normal, or disease states while guiding therapeutic intervention strategies aimed at targeting cell-cell junctions during disease progression.

EXPERIMENTAL PROCEDURES

Generation of E-cadherin-GFP Mice

A cDNA encoding EGFP-linked E-cadherin fusion protein (Lock and Stow, 2005) was cloned into pHPRT.CAAG.STOP downstream of a lox-stop-lox cassette, and the transgene was recombineered into pSKB1 (Bronson et al., 1996) to generate the final targeting vector. Upon germline transmission, the lox-stop-lox-E-cadherin-GFP mice (OFF mice) were bred with tissue-specific Cre driver lines or with CMV-Cre "deleter" mice to generate E-cadherin-GFP ON mice with ubiquitous E-cadherin-GFP expression.

All animal work was carried out under the control of the British Home Office and the local Animal Welfare and Experimental Ethics Committee (Cancer Research UK Beatson Institute and the Garvan Institute of Medical Research/St. Vincent's Hospital Animal Ethics Committee).

Confocal Photobleaching Experiments in Cell Culture, Cell-Derived Matrix and In Vivo

Confluent cells were imaged on 35-mm glass bottom dishes at 37°C and 5% CO_2 using a Leica DMI 6000 SP8 confocal microscope or an Olympus FV1000 confocal microscope with a SIM scanner (Serrels et al., 2009). For photobleaching (FRAP, FLIP) experiments in live xenograft tumors, mice were anesthetized using a combination of 1:1 hypnorm - H_2O + 1:1 hypnovel - H_2O, and the subcutaneous tumor was exposed surgically via a skin flap procedure (Serrels et al., 2009) on a 37°C heated stage. Mice were sacrificed within 4 hr of imaging.

Detailed protocols can be found in the Supplemental Experimental Procedures.

SUPPLEMENTAL INFORMATION

Supplemental Information includes Supplemental Experimental Procedures, six figures, and seven movies and can be found with this article online at http://dx.doi.org/10.1016/j.celrep.2015.12.020.

AUTHOR CONTRIBUTIONS

Methodology, Z.E., D.H., S.C.W., E.J.M., J.P.M., K.I.A., and P.T.; Software, S.C.W. and E.J.M.; Formal Analysis, Z.E., D.H., and S.C.W.; Investigation, Z.E., D.H., S.C.W., M.N., M.C.L., W.L., N.R., A.M., J.P.S., S.K., J.R.W.C., C.V., S.A.K., A.D.C., D.G., A.M., K.J.M., R.A.R., A.M.L., S.N.W., D.R.C., and J.P.M.; Resources, D.G., A.M., S.N.W., S.T.G., D.R.C., L.Z., H.H., E.C.H., P.W.G., C.J.O., T.R.J.E., D.S., O.J.S., and J.P.M.; Conceptualization, K.I.A. and P.T.

ACKNOWLEDGMENTS

The authors thank Dr. Haley Bennett, Dr. Marina Pajic, and Dr. Ilse Rooman for critical reading of the manuscript. P.T., D.H., S.C.W., M.C.L., W.L., N.R., J.R.W.C., C.V., D.G., A.M., K.J.M., A.M.L., D.R.C., and C.J.O. were funded by an NHMRC project grant, an ARC Future grant, a CINSW equipment grant, an Ainsworth Pancreatic Cancer Fellowship, Cancer Council NSW, and a Tour de Cure grant. K.I.A., Z.E., M.N., E.J.M., A.M., J.P.S., S.K., S.A.K., A.D.C., R.A.R., T.R.J.E., D.S., O.J.S., and J.P.M. were funded by a CRUK core grant.

Received: July 2, 2015
Revised: October 21, 2015
Accepted: November 23, 2015
Published: December 24, 2015

REFERENCES

Adams, C.L., Chen, Y.T., Smith, S.J., and Nelson, W.J. (1998). Mechanisms of epithelial cell-cell adhesion and cell compaction revealed by high-resolution tracking of E-cadherin-green fluorescent protein. J. Cell Biol. *142*, 1105–1119.

Avizienyte, E., Wyke, A.W., Jones, R.J., McLean, G.W., Westhoff, M.A., Brunton, V.G., and Frame, M.C. (2002). Src-induced de-regulation of E-cadherin in colon cancer cells requires integrin signalling. Nat. Cell Biol. *4*, 632–638.

Axelrod, D., Koppel, D.E., Schlessinger, J., Elson, E., and Webb, W.W. (1976). Mobility measurement by analysis of fluorescence photobleaching recovery kinetics. Biophys. J. *16*, 1055–1069.

Bardeesy, N., and DePinho, R.A. (2002). Pancreatic cancer biology and genetics. Nat. Rev. Cancer *2*, 897–909.

Biankin, A.V., Waddell, N., Kassahn, K.S., Gingras, M.C., Muthuswamy, L.B., Johns, A.L., Miller, D.K., Wilson, P.J., Patch, A.M., Wu, J., et al.; Australian Pancreatic Cancer Genome Initiative (2012). Pancreatic cancer genomes reveal aberrations in axon guidance pathway genes. Nature *491*, 399–405.

Brabletz, T., Lyden, D., Steeg, P.S., and Werb, Z. (2013). Roadblocks to translational advances on metastasis research. Nat. Med. *19*, 1104–1109.

Bronson, S.K., Plaehn, E.G., Kluckman, K.D., Hagaman, J.R., Maeda, N., and Smithies, O. (1996). Single-copy transgenic mice with chosen-site integration. Proc. Natl. Acad. Sci. USA *93*, 9067–9072.

Calautti, E., Cabodi, S., Stein, P.L., Hatzfeld, M., Kedersha, N., and Paolo Dotto, G. (1998). Tyrosine phosphorylation and src family kinases control keratinocyte cell-cell adhesion. J. Cell Biol. *141*, 1449–1465.

Campbell, K., and Casanova, J. (2015). A role for E-cadherin in ensuring cohesive migration of a heterogeneous population of non-epithelial cells. Nat. Commun. *6*, 7998.

Canel, M., Serrels, A., Anderson, K.I., Frame, M.C., and Brunton, V.G. (2010a). Use of photoactivation and photobleaching to monitor the dynamic regulation of E-cadherin at the plasma membrane. Cell Adhes. Migr. *4*, 491–501.

Canel, M., Serrels, A., Miller, D., Timpson, P., Serrels, B., Frame, M.C., and Brunton, V.G. (2010b). Quantitative in vivo imaging of the effects of inhibiting integrin signaling via Src and FAK on cancer cell movement: effects on E-cadherin dynamics. Cancer Res. *70*, 9413–9422.

Cavey, M., Rauzi, M., Lenne, P.F., and Lecuit, T. (2008). A two-tiered mechanism for stabilization and immobilization of E-cadherin. Nature *453*, 751–756.

Conway, J.R., Carragher, N.O., and Timpson, P. (2014). Developments in preclinical cancer imaging: innovating the discovery of therapeutics. Nat. Rev. Cancer *14*, 314–328.

David, J.M., and Rajasekaran, A.K. (2012). Dishonorable discharge: the oncogenic roles of cleaved E-cadherin fragments. Cancer Res. *72*, 2917–2923.

de Beco, S., Amblard, F., and Coscoy, S. (2012). New insights into the regulation of E-cadherin distribution by endocytosis. Int. Rev. Cell Mol. Biol. *295*, 63–108.

Delva, E., and Kowalczyk, A.P. (2009). Regulation of cadherin trafficking. Traffic *10*, 259–267.

Ellenbroek, S.I., and van Rheenen, J. (2014). Imaging hallmarks of cancer in living mice. Nat. Rev. Cancer *14*, 406–418.

Foote, H.P., Sumigray, K.D., and Lechler, T. (2013). FRAP analysis reveals stabilization of adhesion structures in the epidermis compared to cultured keratinocytes. PLoS ONE *8*, e71491.

Frame, M.C., Fincham, V.J., Carragher, N.O., and Wyke, J.A. (2002). v-Src's hold over actin and cell adhesions. Nat. Rev. Mol. Cell Biol. *3*, 233–245.

Friedl, P., Locker, J., Sahai, E., and Segall, J.E. (2012). Classifying collective cancer cell invasion. Nat. Cell Biol. *14*, 777–783.

Fritzsche, M., and Charras, G. (2015). Dissecting protein reaction dynamics in living cells by fluorescence recovery after photobleaching. Nat. Protoc. *10*, 660–680.

Gregory, P.A., Bert, A.G., Paterson, E.L., Barry, S.C., Tsykin, A., Farshid, G., Vadas, M.A., Khew-Goodall, Y., and Goodall, G.J. (2008). The miR-200 family and miR-205 regulate epithelial to mesenchymal transition by targeting ZEB1 and SIP1. Nat. Cell Biol. *10*, 593–601.

Halbleib, J.M., and Nelson, W.J. (2006). Cadherins in development: cell adhesion, sorting, and tissue morphogenesis. Genes Dev. *20*, 3199–3214.

Haubeiss, S., Schmid, J.O., Mürdter, T.E., Sonnenberg, M., Friedel, G., van der Kuip, H., and Aulitzky, W.E. (2010). Dasatinib reverses cancer-associated fibroblasts (CAFs) from primary lung carcinomas to a phenotype comparable to that of normal fibroblasts. Mol. Cancer *9*, 168.

Herrmann, D., Conway, J.R., Vennin, C., Magenau, A., Hughes, W.E., Morton, J.P., and Timpson, P. (2014). Three-dimensional cancer models mimic cell-matrix interactions in the tumour microenvironment. Carcinogenesis *35*, 1671–1679.

Hingorani, S.R., Petricoin, E.F., Maitra, A., Rajapakse, V., King, C., Jacobetz, M.A., Ross, S., Conrads, T.P., Veenstra, T.D., Hitt, B.A., et al. (2003). Preinvasive and invasive ductal pancreatic cancer and its early detection in the mouse. Cancer Cell *4*, 437–450.

Hingorani, S.R., Wang, L., Multani, A.S., Combs, C., Deramaudt, T.B., Hruban, R.H., Rustgi, A.K., Chang, S., and Tuveson, D.A. (2005). Trp53R172H and KrasG12D cooperate to promote chromosomal instability and widely metastatic pancreatic ductal adenocarcinoma in mice. Cancer Cell *7*, 469–483.

Hu, W., Li, H., Wang, C., Gou, S., and Fu, L. (2012). Characterization of collagen fibers by means of texture analysis of second harmonic generation images using orientation-dependent gray level co-occurrence matrix method. J. Biomed. Opt. *17*, 026007.

Ireland, H., Kemp, R., Houghton, C., Howard, L., Clarke, A.R., Sansom, O.J., and Winton, D.J. (2004). Inducible Cre-mediated control of gene expression in the murine gastrointestinal tract: effect of loss of beta-catenin. Gastroenterology *126*, 1236–1246.

Johnsson, A.K., Dai, Y., Nobis, M., Baker, M.J., McGhee, E.J., Walker, S., Schwarz, J.P., Kadir, S., Morton, J.P., Myant, K.B., et al. (2014). The Rac-FRET mouse reveals tight spatiotemporal control of Rac activity in primary cells and tissues. Cell Rep. *6*, 1153–1164.

Kennedy, A.L., Morton, J.P., Manoharan, I., Nelson, D.M., Jamieson, N.B., Pawlikowski, J.S., McBryan, T., Doyle, B., McKay, C., Oien, K.A., et al. (2011). Activation of the PIK3CA/AKT pathway suppresses senescence induced by an activated RAS oncogene to promote tumorigenesis. Mol. Cell *42*, 36–49.

Lamouille, S., Xu, J., and Derynck, R. (2014). Molecular mechanisms of epithelial-mesenchymal transition. Nat. Rev. Mol. Cell Biol. *15*, 178–196.

Levitt, J.A.M., Morton, P.E., Fruhwirth, G.O., Santis, G., Chung, P.H., Parsons, M., and Suhling, K. (2015). Simultaneous FRAP, FLIM and FAIM for measurements of protein mobility and interaction in living cells. Biomed. Opt. Express *6*, 3842–3854.

Ling, J., Kang, Y., Zhao, R., Xia, Q., Lee, D.F., Chang, Z., Li, J., Peng, B., Fleming, J.B., Wang, H., et al. (2012). KrasG12D-induced IKK2/β/NF-κB activation by IL-1α and p62 feedforward loops is required for development of pancreatic ductal adenocarcinoma. Cancer Cell *21*, 105–120.

Lippincott-Schwartz, J., Snapp, E., and Kenworthy, A. (2001). Studying protein dynamics in living cells. Nat. Rev. Mol. Cell Biol. *2*, 444–456.

Lock, J.G., and Stow, J.L. (2005). Rab11 in recycling endosomes regulates the sorting and basolateral transport of E-cadherin. Mol. Biol. Cell *16*, 1744–1755.

Manning, C.S., Jenkins, R., Hooper, S., Gerhardt, H., Marais, R., Adams, S., Adams, R.H., van Rheenen, J., and Sahai, E. (2013). Intravital imaging reveals conversion between distinct tumor vascular morphologies and localized vascular response to Sunitinib. Intravital *2*, e24790.

Morran, D.C., Wu, J., Jamieson, N.B., Mrowinska, A., Kalna, G., Karim, S.A., Au, A.Y., Scarlett, C.J., Chang, D.K., Pajak, M.Z., et al.; Australian Pancreatic Cancer Genome Initiative (APGI) (2014). Targeting mTOR dependency in pancreatic cancer. Gut *63*, 1481–1489.

Morton, J.P., Karim, S.A., Graham, K., Timpson, P., Jamieson, N., Athineos, D., Doyle, B., McKay, C., Heung, M.Y., Oien, K.A., et al. (2010a). Dasatinib inhibits the development of metastases in a mouse model of pancreatic ductal adenocarcinoma. Gastroenterology *139*, 292–303.

Morton, J.P., Timpson, P., Karim, S.A., Ridgway, R.A., Athineos, D., Doyle, B., Jamieson, N.B., Oien, K.A., Lowy, A.M., Brunton, V.G., et al. (2010b). Mutant p53 drives metastasis and overcomes growth arrest/senescence in pancreatic cancer. Proc. Natl. Acad. Sci. USA *107*, 246–251.

Muller, P.A., and Vousden, K.H. (2014). Mutant p53 in cancer: new functions and therapeutic opportunities. Cancer Cell *25*, 304–317.

Muller, P.A., Caswell, P.T., Doyle, B., Iwanicki, M.P., Tan, E.H., Karim, S., Lukashchuk, N., Gillespie, D.A., Ludwig, R.L., Gosselin, P., et al. (2009). Mutant p53 drives invasion by promoting integrin recycling. Cell *139*, 1327–1341.

Muller, P.A., Trinidad, A.G., Timpson, P., Morton, J.P., Zanivan, S., van den Berghe, P.V., Nixon, C., Karim, S.A., Caswell, P.T., Noll, J.E., et al. (2013). Mutant p53 enhances MET trafficking and signalling to drive cell scattering and invasion. Oncogene *32*, 1252–1265.

Nam, H.J., Im, S.A., Oh, D.Y., Elvin, P., Kim, H.P., Yoon, Y.K., Min, A., Song, S.H., Han, S.W., Kim, T.Y., and Bang, Y.J. (2013). Antitumor activity of saracatinib (AZD0530), a c-Src/Abl kinase inhibitor, alone or in combination with chemotherapeutic agents in gastric cancer. Mol. Cancer Ther. *12*, 16–26.

Nobis, M., McGhee, E.J., Morton, J.P., Schwarz, J.P., Karim, S.A., Quinn, J., Edward, M., Campbell, A.D., McGarry, L.C., Evans, T.R., et al. (2013). Intravital FLIM-FRET imaging reveals dasatinib-induced spatial control of src in pancreatic cancer. Cancer Res. *73*, 4674–4686.

Nobis, M., McGhee, E.J., Herrmann, D., Magenau, A., Morton, J.P., Anderson, K.I., and Timpson, P. (2014). Monitoring the dynamics of Src activity in response to anti-invasive dasatinib treatment at a subcellular level using dual intravital imaging. Cell Adhes. Migr. *8*, 478–486.

Ocaña, O.H., Córcoles, R., Fabra, A., Moreno-Bueno, G., Acloque, H., Vega, S., Barrallo-Gimeno, A., Cano, A., and Nieto, M.A. (2012). Metastatic colonization requires the repression of the epithelial-mesenchymal transition inducer Prrx1. Cancer Cell *22*, 709–724.

Postic, C., Shiota, M., Niswender, K.D., Jetton, T.L., Chen, Y., Moates, J.M., Shelton, K.D., Lindner, J., Cherrington, A.D., and Magnuson, M.A. (1999). Dual roles for glucokinase in glucose homeostasis as determined by liver and pancreatic beta cell-specific gene knock-outs using Cre recombinase. J. Biol. Chem. *274*, 305–315.

Priya, R., Yap, A.S., and Gomez, G.A. (2013). E-cadherin supports steady-state Rho signaling at the epithelial zonula adherens. Differentiation *86*, 133–140.

Ritsma, L., Steller, E.J., Beerling, E., Loomans, C.J., Zomer, A., Gerlach, C., Vrisekoop, N., Seinstra, D., van Gurp, L., Schäfer, R., et al. (2012). Intravital microscopy through an abdominal imaging window reveals a pre-micrometastasis stage during liver metastasis. Sci. Transl. Med. *4*, 158ra145.

Roberti, M.J., Jovin, T.M., and Jares-Erijman, E. (2011). Confocal fluorescence anisotropy and FRAP imaging of α-synuclein amyloid aggregates in living cells. PLoS ONE *6*, e23338.

Sakurai-Yageta, M., Maruyama, T., Suzuki, T., Ichikawa, K., and Murakami, Y. (2015). Dynamic regulation of a cell adhesion protein complex including CADM1 by combinatorial analysis of FRAP with exponential curve-fitting. PLoS ONE *10*, e0116637.

Schwenk, F., Baron, U., and Rajewsky, K. (1995). A cre-transgenic mouse strain for the ubiquitous deletion of loxP-flanked gene segments including deletion in germ cells. Nucleic Acids Res. *23*, 5080–5081.

Serrels, A., Timpson, P., Canel, M., Schwarz, J.P., Carragher, N.O., Frame, M.C., Brunton, V.G., and Anderson, K.I. (2009). Real-time study of E-cadherin and membrane dynamics in living animals: implications for disease modeling and drug development. Cancer Res. *69*, 2714–2719.

Shamir, E.R., Pappalardo, E., Jorgens, D.M., Coutinho, K., Tsai, W.T., Aziz, K., Auer, M., Tran, P.T., Bader, J.S., and Ewald, A.J. (2014). Twist1-induced dissemination preserves epithelial identity and requires E-cadherin. J. Cell Biol. *204*, 839–856.

Shen, L., Weber, C.R., and Turner, J.R. (2008). The tight junction protein complex undergoes rapid and continuous molecular remodeling at steady state. J. Cell Biol. *181*, 683–695.

Sprague, B.L., and McNally, J.G. (2005). FRAP analysis of binding: proper and fitting. Trends Cell Biol. *15*, 84–91.

Tan, E.H., Morton, J.P., Timpson, P., Tucci, P., Melino, G., Flores, E.R., Sansom, O.J., Vousden, K.H., and Muller, P.A. (2014). Functions of TAp63 and p53 in restraining the development of metastatic cancer. Oncogene *33*, 3325–3333.

Timpson, P., McGhee, E.J., Morton, J.P., von Kriegsheim, A., Schwarz, J.P., Karim, S.A., Doyle, B., Quinn, J.A., Carragher, N.O., Edward, M., et al. (2011). Spatial regulation of RhoA activity during pancreatic cancer cell invasion driven by mutant p53. Cancer Res. *71*, 747–757.

Vlahov, N., Scrace, S., Soto, M.S., Grawenda, A.M., Bradley, L., Pankova, D., Papaspyropoulos, A., Yee, K.S., Buffa, F., Goding, C.R., et al. (2015). Alternate RASSF1 Transcripts Control SRC Activity, E-Cadherin Contacts, and YAP-Mediated Invasion. Curr. Biol. S0960-9822(15)01227-0. http://dx.doi.org/10.1016/j.cub.2015.09.072.

von Bonsdorff, C.H., Fuller, S.D., and Simons, K. (1985). Apical and basolateral endocytosis in Madin-Darby canine kidney (MDCK) cells grown on nitrocellulose filters. EMBO J. *4*, 2781–2792.

Wagner, K.U., Wall, R.J., St-Onge, L., Gruss, P., Wynshaw-Boris, A., Garrett, L., Li, M., Furth, P.A., and Hennighausen, L. (1997). Cre-mediated gene deletion in the mammary gland. Nucleic Acids Res. *25*, 4323–4330.

Wicki, A., Lehembre, F., Wick, N., Hantusch, B., Kerjaschki, D., and Christofori, G. (2006). Tumor invasion in the absence of epithelial-mesenchymal transition: podoplanin-mediated remodeling of the actin cytoskeleton. Cancer Cell *9*, 261–272.

Wu, S.K., Gomez, G.A., Michael, M., Verma, S., Cox, H.L., Lefevre, J.G., Parton, R.G., Hamilton, N.A., Neufeld, Z., and Yap, A.S. (2014). Cortical F-actin stabilization generates apical-lateral patterns of junctional contractility that integrate cells into epithelia. Nat. Cell Biol. *16*, 167–178.

Wu, Y., Kanchanawong, P., and Zaidel-Bar, R. (2015). Actin-delimited adhesion-independent clustering of E-cadherin forms the nanoscale building blocks of adherens junctions. Dev. Cell *32*, 139–154.

Yamada, K.M., and Cukierman, E. (2007). Modeling tissue morphogenesis and cancer in 3D. Cell *130*, 601–610.

Yang, J.C., Bai, L., Yap, S., Gao, A.C., Kung, H.J., and Evans, C.P. (2010). Effect of the specific Src family kinase inhibitor saracatinib on osteolytic lesions using the PC-3 bone model. Mol. Cancer Ther. *9*, 1629–1637.

Cell Reports

Supplemental Information

Intravital FRAP Imaging using an E-cadherin-GFP Mouse Reveals Disease- and Drug-Dependent Dynamic Regulation of Cell-Cell Junctions in Live Tissue

Zahra Erami, David Herrmann, Sean C. Warren, Max Nobis, Ewan J. McGhee, Morghan C. Lucas, Wilfred Leung, Nadine Reischmann, Agata Mrowinska, Juliane P. Schwarz, Shereen Kadir, James R.W. Conway, Claire Vennin, Saadia A. Karim, Andrew D. Campbell, David Gallego-Ortega, Astrid Magenau, Kendelle J. Murphy, Rachel A. Ridgway, Andrew M. Law, Stacey N. Walters, Shane T. Grey, David R. Croucher, Lei Zhang,, Herbert Herzog, Edna C. Hardeman, Peter W. Gunning, Christopher J. Ormandy, T.R. Jeffry Evans, Douglas Strathdee, Owen J. Sansom, Jennifer P. Morton, Kurt I. Anderson, and Paul Timpson

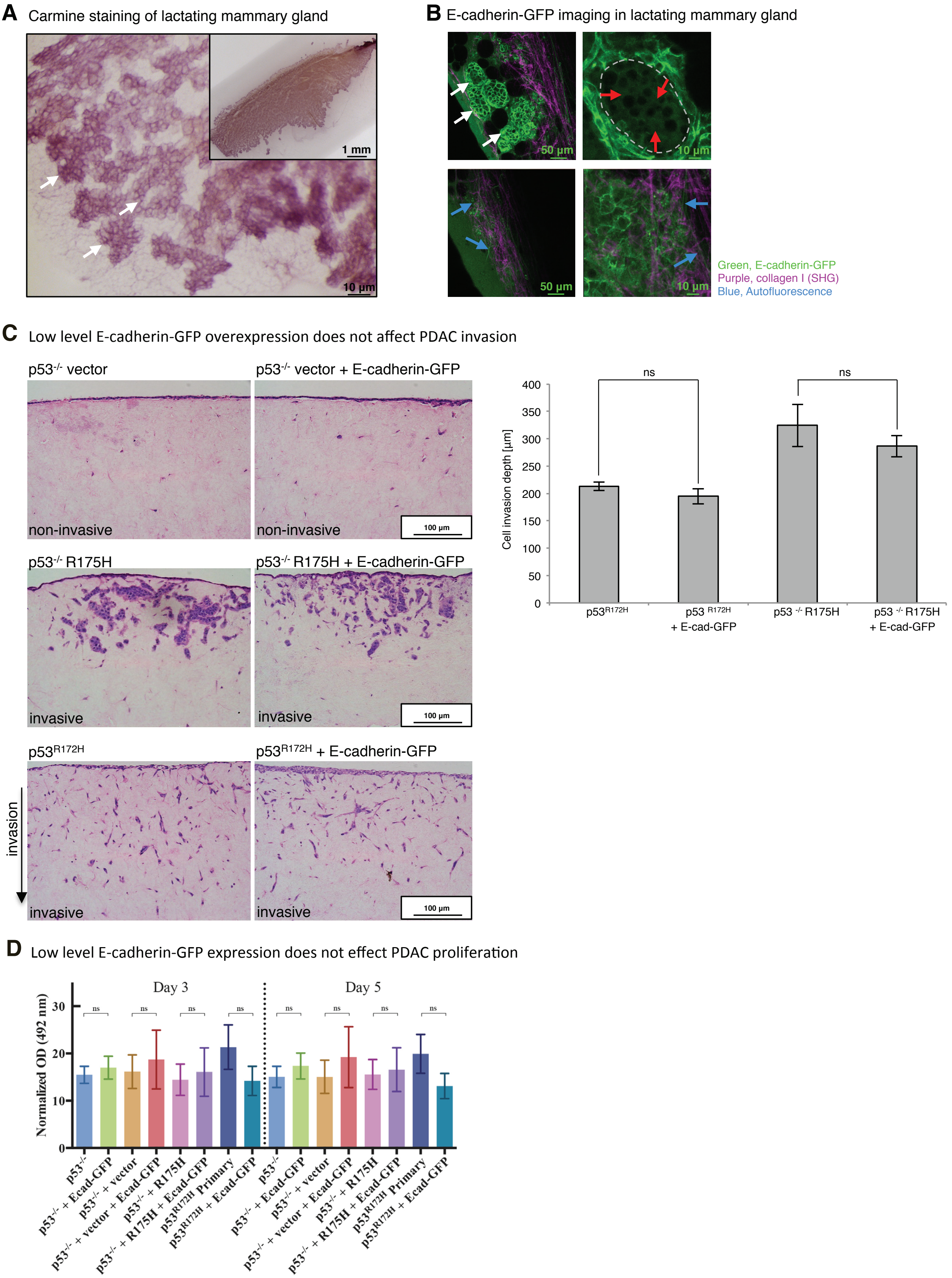
A Carmine staining of lactating mammary gland
1 mm
10 µm
B E-cadherin-GFP imaging in lactating mammary gland
50 µm
10 µm
50 µm
10 µm
Green, E-cadherin-GFP
Purple, collagen I (SHG)
Blue, Autofluorescence
C Low level E-cadherin-GFP overexpression does not affect PDAC invasion
p53-/- vector
p53-/- vector + E-cadherin-GFP
non-invasive
non-invasive
100 µm
p53-/- R175H
p53-/- R175H + E-cadherin-GFP
invasive
invasive
100 µm
p53R172H
p53R172H + E-cadherin-GFP
invasion
invasive
invasive
100 µm
Cell invasion depth [µm]
0
50
100
150
200
250
300
350
400
ns
ns
p53R172H
p53 R172H + E-cad-GFP
p53 -/- R175H
p53 -/- R175H + E-cad-GFP
D Low level E-cadherin-GFP expression does not effect PDAC proliferation
Normalized OD (492 nm)
0
10
20
30
Day 3
Day 5
ns
p53-/-
p53-/- + Ecad-GFP
p53-/- + vector
p53-/- + vector + Ecad-GFP
p53-/- + R175H
p53-/- + R175H + Ecad-GFP
p53R172H Primary
p53R172H + Ecad-GFP

Figure S1, related to Figure 1. Expressing E-cadherin-GFP in PDAC cells does not impair cell invasion and proliferation.

(A) Carmine staining of the whole-mounted lactating mammary gland (inset) showing fully developed alveoli (white arrows). (B) Representative still images of E-cadherin-GFP cell-cell junctions in the mammary gland at low (left panel) and high magnification (right panel). Expression is driven via MMTV-Cre. E-cadherin-GFP, green; SHG signal, purple. Note elastin autofluorescence (blue arrows). (C) Representative H&E images of primary PDAC cells on 3D-organotypic matrices with or without E-cadherin-GFP expression and quantification of cell invasion (bar, 100 µm). (D) Growth of primary cell lines from $p53^{-/-}$ and $p53^{R172H}$ tumors demonstrating that low-level expression of E-cadherin-GFP has no significant effect on tumor cell proliferation. Cells were stably transfected in combinations of vector alone, $p53^{R175H}$ and/or E-cadherin-GFP. Columns, mean; bars, ± SE. *, $P < 0.05$; **, $P < 0.01$ by unpaired Student's t-test.

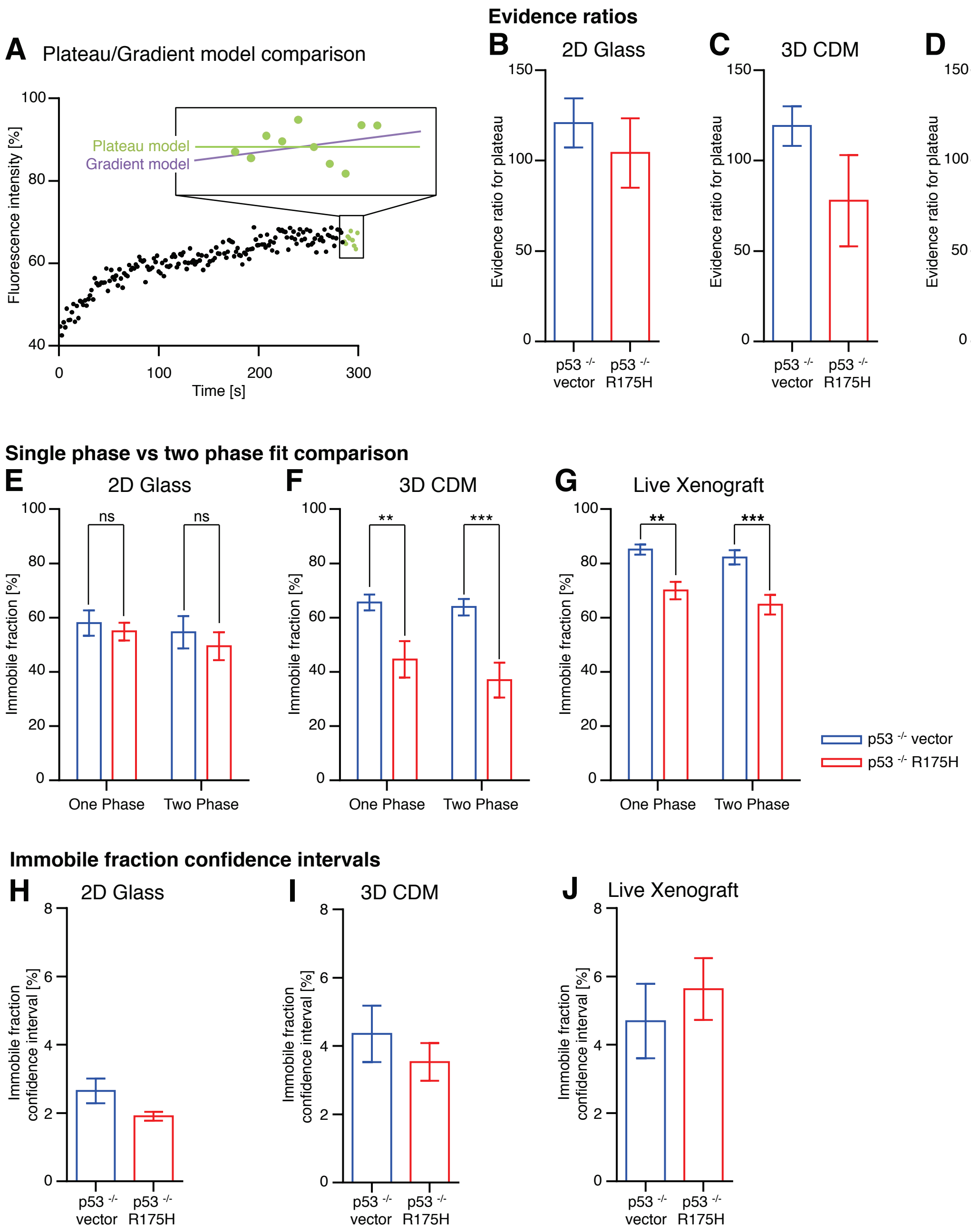

Evidence ratios
A
Plateau/Gradient model comparison
Plateau model
Gradient model
Fluorescence intensity [%]
Time [s]
B
2D Glass
C
3D CDM
D
Live Xenograft
Evidence ratio for plateau
p53 -/- vector
p53 -/- R175H
Single phase vs two phase fit comparison
E
2D Glass
F
3D CDM
G
Live Xenograft
Immobile fraction [%]
ns
**
***
One Phase
Two Phase
p53 -/- vector
p53 -/- R175H
Immobile fraction confidence intervals
H
2D Glass
I
3D CDM
J
Live Xenograft
Immobile fraction confidence interval [%]

Figure S2, related to Figures 2,3. Validation of FRAP quantification.

(A) Cartoon of approach used to determine the relative likelihood that a curve has reached a plateau (green line) or is continuing to rise (purple line) using model comparison. (B-D) Graph of evidence ratio for a plateau, compared to a gradient, for the final ten points in the FRAP recovery curve for $p53^{-/-}$ vector (blue) and $p53^{-/-}$ R175H cells (red) grown on glass (B), CDM (C) or in a xenograft (D). Evidence ratios larger than 1 indicate that it is more likely that the curve has plateaued. (E-G) Immobile fractions calculated when using one (left) and two phase FRAP recovery models (right) for $p53^{-/-}$ vector (blue) and $p53^{-/-}$ R175H cells (red) grown on glass (E), CDM (F) and in a xenograft (G). (H-J) Confidence interval on value of fitted immobile fraction calculated using the Delta method for $p53^{-/-}$ vector (blue) and $p53^{-/-}$ R175H cells plated on glass (H), CDM (I) and in a xenograft (J) Columns, mean; bars, ± SE. *, $P < 0.05$; **, $P < 0.01$; ***, $P < 0.001$ by unpaired Student's t-test.

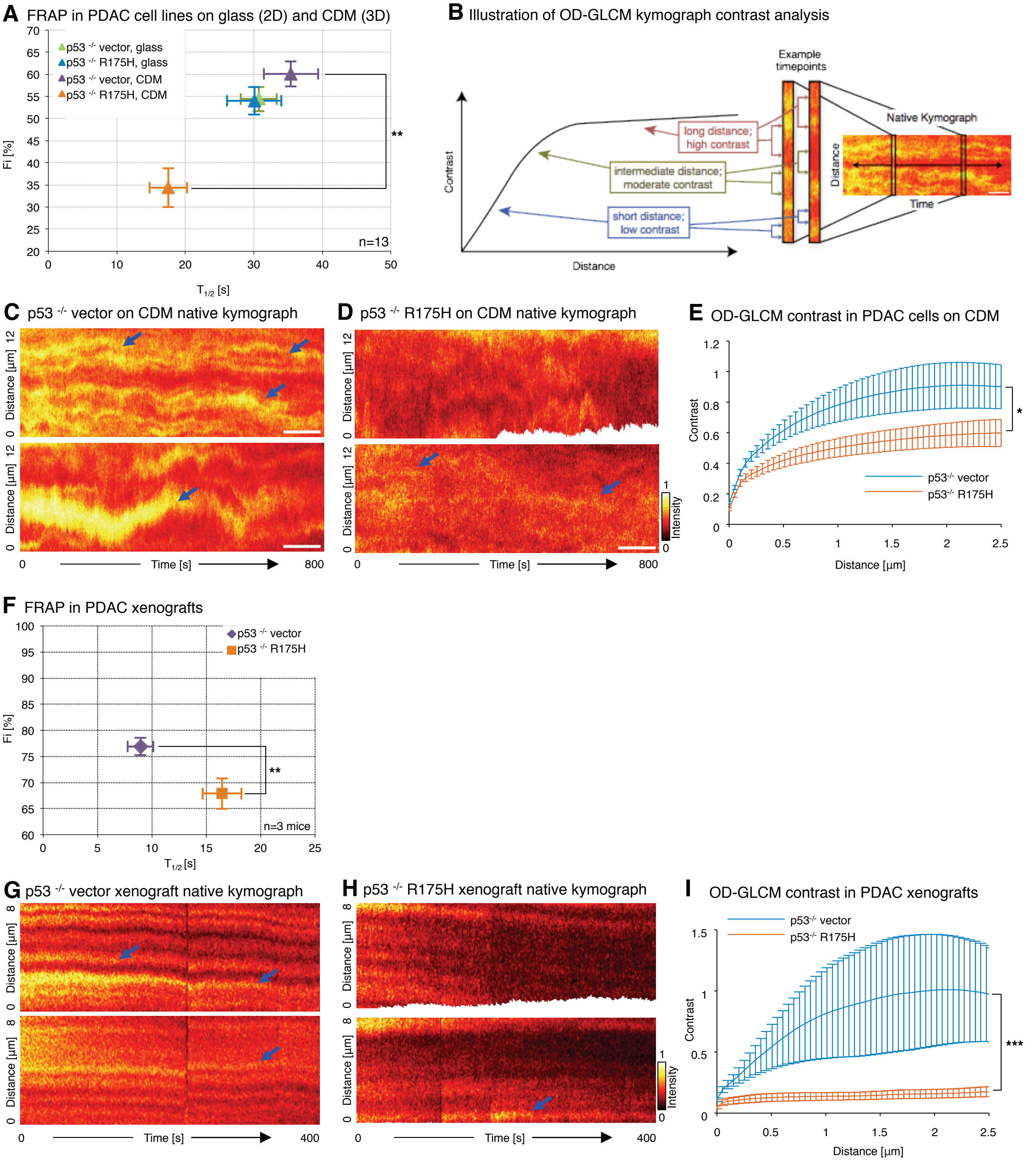

A FRAP in PDAC cell lines on glass (2D) and CDM (3D)
p53 -/- vector, glass
p53 -/- R175H, glass
p53 -/- vector, CDM
p53 -/- R175H, CDM
Fi [%]
T1/2 [s]
n=13
**
B Illustration of OD-GLCM kymograph contrast analysis
Example timepoints
Native Kymograph
long distance; high contrast
intermediate distance; moderate contrast
short distance; low contrast
Contrast
Distance
Time
C p53 -/- vector on CDM native kymograph
D p53 -/- R175H on CDM native kymograph
Distance [µm]
Time [s]
Intensity
E OD-GLCM contrast in PDAC cells on CDM
p53-/- vector
p53-/- R175H
Contrast
Distance [µm]
*
F FRAP in PDAC xenografts
p53 -/- vector
p53 -/- R175H
Fi [%]
T1/2 [s]
n=3 mice
**
G p53 -/- vector xenograft native kymograph
H p53 -/- R175H xenograft native kymograph
Distance [µm]
Time [s]
Intensity
I OD-GLCM contrast in PDAC xenografts
p53-/- vector
p53-/- R175H
Contrast
Distance [µm]
***

Figure S3, related to Figures 2,3. E-cadherin-GFP FRAP, FLIP and OD-GLCM analysis in non-invasive versus invasive PDAC cells on CDMs and in xenografts.

(A) Graphs comparing the mobilization of E-cadherin-GFP in cell-cell junctions between $p53^{-/-}$ vector and $p53^{-/-}$ R175H cells on CDMs. (B) Cartoon schematically illustrating the OD-GLCM kymograph contrast parameter. Each point on the graph gives a measure of the contrast between points along the junction at a particular distances from one another, averaged over the kymograph. This example shows two spatial profiles with specific examples illustrating how pairs of points contribute to the OD-GLCM contrast at short (blue), intermediate (yellow) and long (red) distances. (C,D) Representative kymographs of the native, unbleached junctions for $p53^{-/-}$ vector (C) and $p53^{-/-}$ R175H cells (D). Blue arrows indicate persistent regions of high intensity. Changes in the size of the kymograph represent slight changes in length of the junction over the imaging period. (E) OD-GLCM contrast as a function of distance for the $p53^{-/-}$ vector cells (blue, n=30) and $p53^{-/-}$ R175H cells (red, n=28). Columns, mean; bars, ± SE; *, $P < 0.05$; **, $P < 0.01$ by unpaired Student's t-test. (F) Graphs comparing the mobilization of E-cadherin-GFP in cell-cell junctions between $p53^{-/-}$ vector and $p53^{-/-}$ R175H cells on CDMs. (G,H) Representative kymographs of the native, unbleached junctions for $p53^{-/-}$ vector (G) and $p53^{-/-}$ R175H (H) xenografts. Blue arrows indicate persistent regions of high intensity. Slight discontinuities in the kymographs are due to movement of the imaging region to correct for drift. Changes in the size of the kymograph represent slight changes in length of the junction over the imaging period. (I) GLCM contrast as a function of distance for the $p53^{-/-}$ vector (blue, n=18) and $p53^{-/-}$ R175H xenografts (red, n=10). Statistical significance for OD-GLCM was calculated based on the average contrast. Columns, mean; bars, ± SE; *, $P < 0.05$; **, $P < 0.01$ by unpaired Student's t-test.

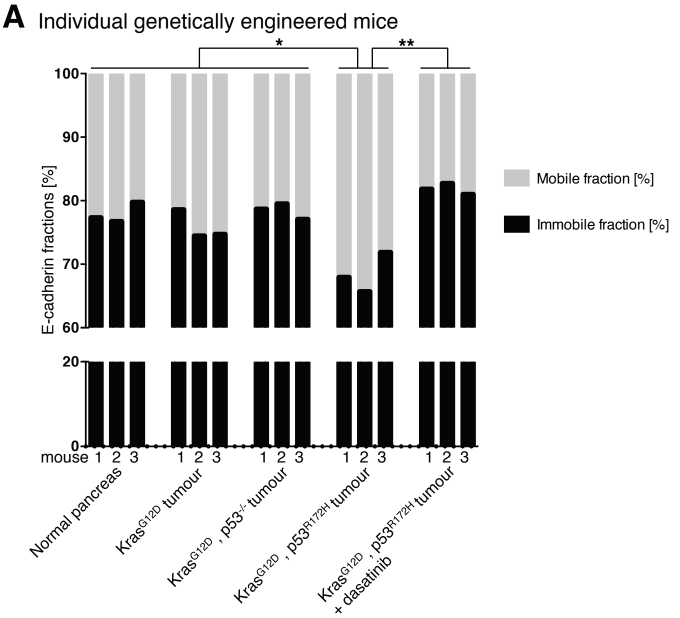

		Immobile fraction [%]	Mobile fraction [%]
**Wildtype pancreas**	mouse 1	77.3	22.7
	mouse 2	76.7	23.3
	mouse 3	79.7	20.3
**Kras^{G12D} tumour**	mouse 1	78.6	21.4
	mouse 2	74.5	25.5
	mouse 3	74.7	25.3
**Kras^{G12D}; p53$^{-/-}$ tumour**	mouse 1	78.7	21.3
	mouse 2	79.5	20.5
	mouse 3	77.1	22.9
**Kras^{G12D}; p53^{R172H} tumour**	mouse 1	67.9	32.1
	mouse 2	65.7	34.3
	mouse 3	71.9	28.1
**Kras^{G12D}; p53^{R172H} tumour + dasatinib**	mouse 1	81.8	18.2
	mouse 2	82.7	17.3
	mouse 3	81.0	19.0

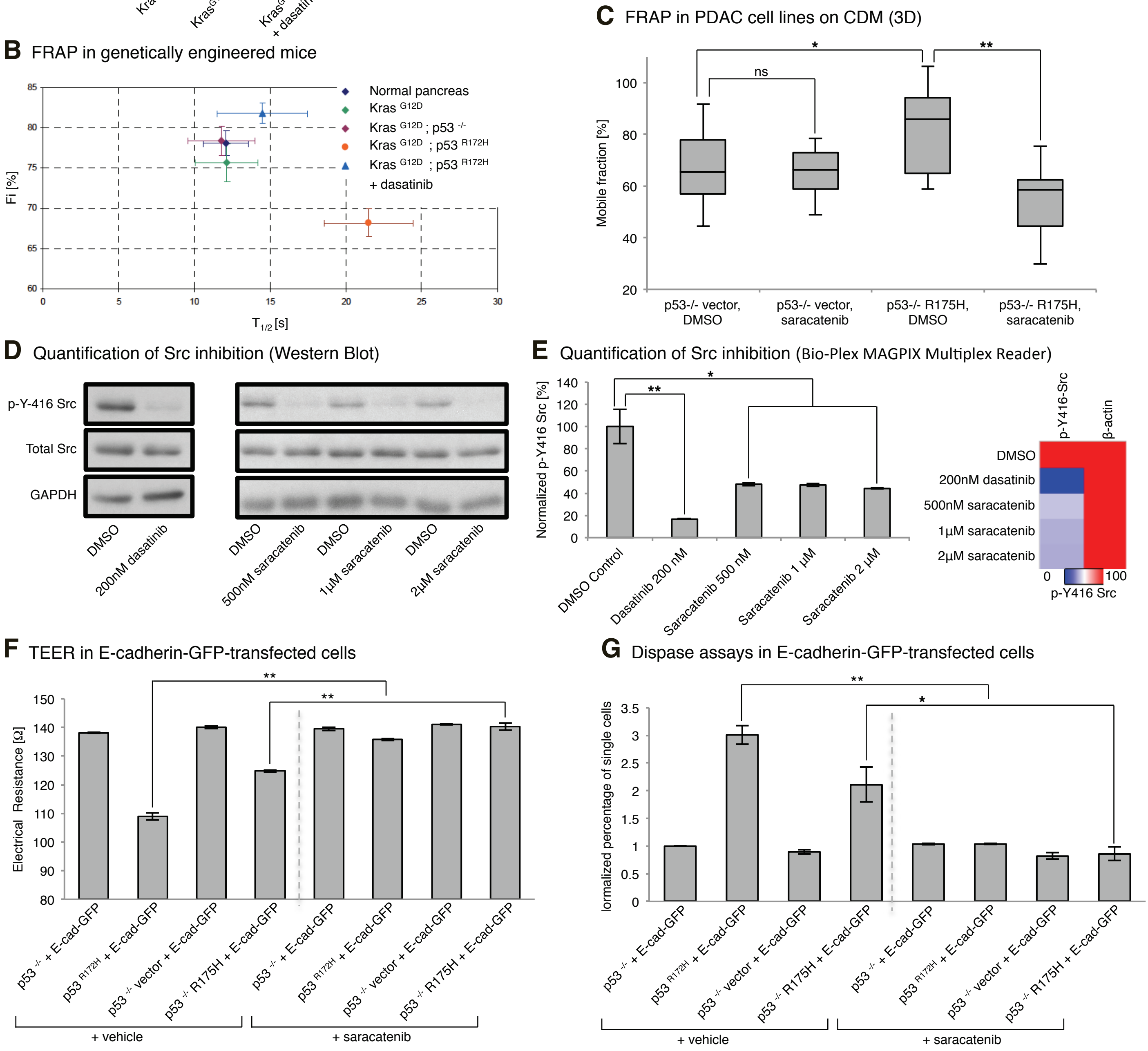

Figure S4, related to Figures 5,6. E-cadherin-GFP FRAP in *in situ* pancreatic tissue.

(A) Graph and table showing an increase in E-cadherin mobility in $Kras^{G12D}$; $p53^{R172H}$ tumors compared to normal pancreas, tumors of $Kras^{G12D}$ alone, $Kras^{G12D}$; $p53^{-/-}$ $Kras^{G12D}$; $p53^{R172H}$ and tumors of dasatinib-treated $Kras^{G12D}$; $p53^{R172H}$ mice for each mouse analyzed. (B) Graphs comparing the mobilization of E-cadherin-GFP in cell-cell junctions between normal pancreas, tumors of $Kras^{G12D}$ alone, $Kras^{G12D}$; $p53^{-/-}$ $Kras^{G12D}$; $p53^{R172H}$ and tumors of dasatinib-treated $Kras^{G12D}$; $p53^{R172H}$ mice (C) Graphs comparing the mobilization of E-cadherin-GFP in cell-cell junctions between $p53^{-/-}$ vector and $p53^{-/-}$ R175H cells grown on CDMs after treatment with 2μM saracatenib or DMSO for 4h; > 10 junctions per group. (D,E) Assessment of phospho-Y416 Src following treatment of $p53^{-/-}$ R175H cells with dasatinib or saracatenib for 4 hours with Western Blot analysis (D) and using Bio-Plex MAGPIX Multiplex Reader (E). (F,G) Trans-Endothelial Electrical Resistance (TEER) measurements of cell-cell junction integrity (F) and Dispase-based mechanical disruption (G) in non-invasive, E-cadherin-GFP-transfected $p53^{-/-}$ PDAC cells derived from Pdx1-Cre; $Kras^{G12D/+}$; $p53^{-/-}$ mice (columns, 1 and 3, pre-saracatenib and columns, 5 and 7, post- saracatenib) versus invasive, E-cadherin-GFP-transfected mutant p53 bearing PDAC cells (columns, 2 and 4, pre- saracatenib and columns, 6 and 8, post-saracatenib). Columns, mean; bars, ± SE, n = 3 per group, *, $P <0.05$; **, $P < 0.01$ by unpaired Student's t-test.

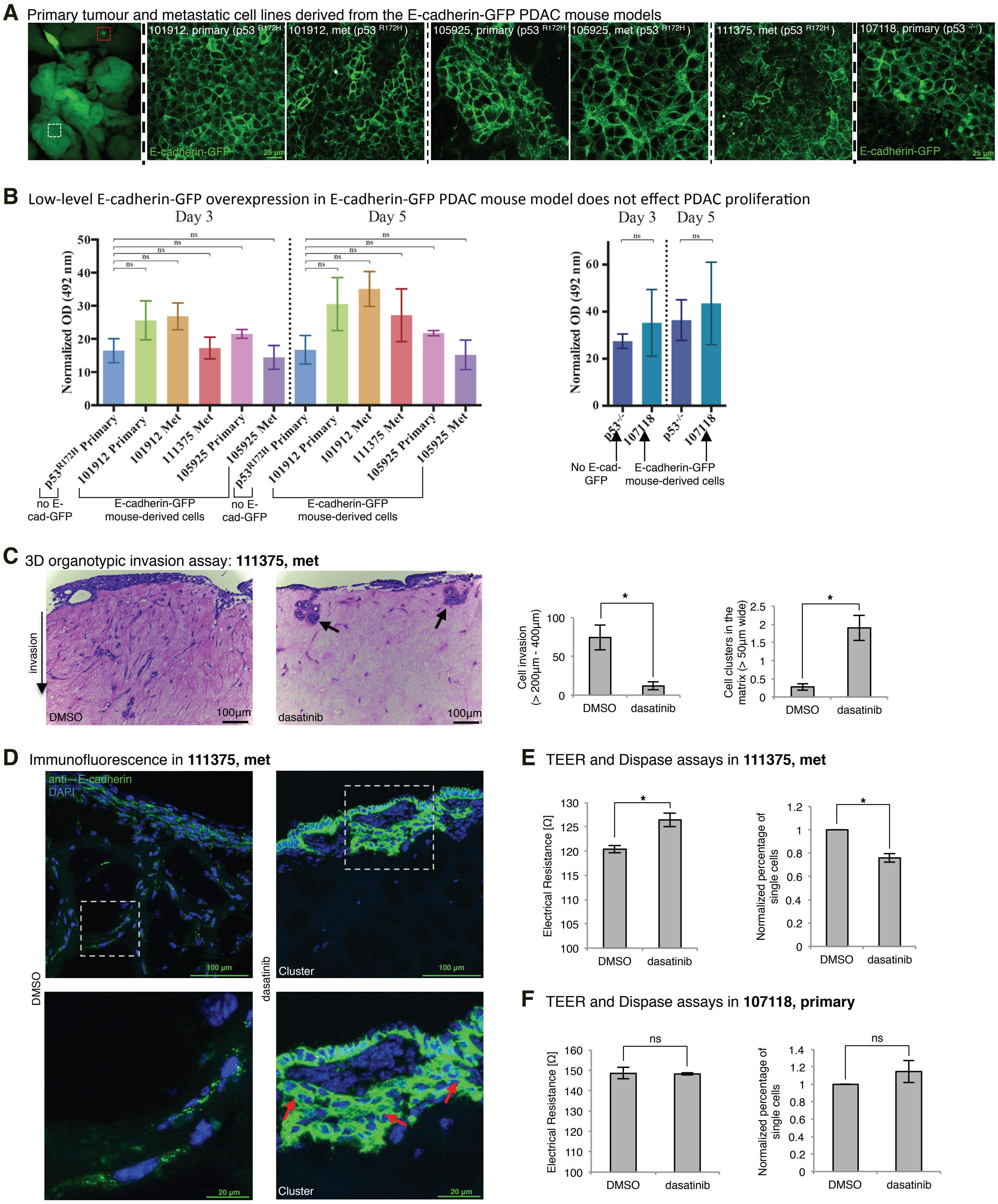
A Primary tumour and metastatic cell lines derived from the E-cadherin-GFP PDAC mouse models
101912, primary (p53 R172H)
101912, met (p53 R172H)
105925, primary (p53 R172H)
105925, met (p53 R172H)
111375, met (p53 R172H)
107118, primary (p53 -/-)
E-cadherin-GFP
25 μm
B Low-level E-cadherin-GFP overexpression in E-cadherin-GFP PDAC mouse model does not effect PDAC proliferation
Day 3
Day 5
Normalized OD (492 nm)
ns
p53R172H Primary
101912 Primary
101912 Met
111375 Met
105925 Primary
105925 Met
no E-cad-GFP
E-cadherin-GFP mouse-derived cells
p53-/-
107118
No E-cad-GFP
C 3D organotypic invasion assay: 111375, met
invasion
DMSO
dasatinib
100μm
Cell invasion (> 200μm - 400μm)
Cell clusters in the matrix (> 50μm wide)
D Immunofluorescence in 111375, met
anti – E-cadherin
DAPI
Cluster
100 μm
20 μm
E TEER and Dispase assays in 111375, met
Electrical Resistance [Ω]
Normalized percentage of single cells
F TEER and Dispase assays in 107118, primary

Figure S5, related to Figure 7. E-cadherin-GFP expression in primary cells derived from Pdx1-Cre; Kras^{G12D}; p53R^{172H}; E-cadherin-GFP mice with dasatinib-induced cluster formation in 3D organotypic matrices.

(A) Whole body fluorescent image of primary tumor and isolated liver micro-metastases of a Pdx1-Cre; Kras^{G12D}; p53^{R172H}; E-cadherin-GFP mouse. Cell lines derived from the primary tumor and liver metastases of 3 independent mice (101912, 105925, 111375) as well as cells derived from the primary tunor of a Pdx1-Cre; Kras^{G12D}; p53$^{-/-}$; E-cadherin-GFP mouse (107118, primary) were chosen for further analysis. (B) Growth of primary cell lines from the Pdx1-Cre; Kras^{G12D}; p53^{R172H}; E-cadherin-GFP mouse was comparable to p53^{R172H} cells without E-cadherin-GFP expression (left panel). Growth of primary cell lines from the Pdx1-Cre; Kras^{G12D}; p53$^{-/-}$; E-cadherin-GFP mouse was comparable to p53$^{-/-}$ cells without E-cadherin-GFP expression (right panel). (C) Isolated secondary metastatic cell line (111375, met) from the liver of a Pdx1-Cre; Kras^{G12D}; p53^{R172H}; E-cadherin-GFP mouse invading into 3D-organotypic matrices ± dasatinib (13 days, 200 nM). Black arrows indicate cluster formation upon dasatinib treatment. Quantification of cell invasion as number of cells invaded beyond > 200 μm and quantification of cell clustering within matrices ± dasatinib (13 days, 200 nM). (D) Immunofluorescence staining of E-cadherin within matrices ± dasatinib (13 days, 200 nM) for metastatic line 111375 met. Red arrows indicate stabilized E-cadherin-GFP expression within cell clusters within the matrix. Anti-E-cadherin in green, DAPI in blue. (E,F) TEER measurements (left graph) and Dispase-based mechanical disruption (right graph) in 111375 met ± dasatinib (E) and 107118 ± dasatinib (F). Columns, mean; bars, ± SE; n = 3 per group; *, $P < 0.05$ by unpaired Student's t-test.

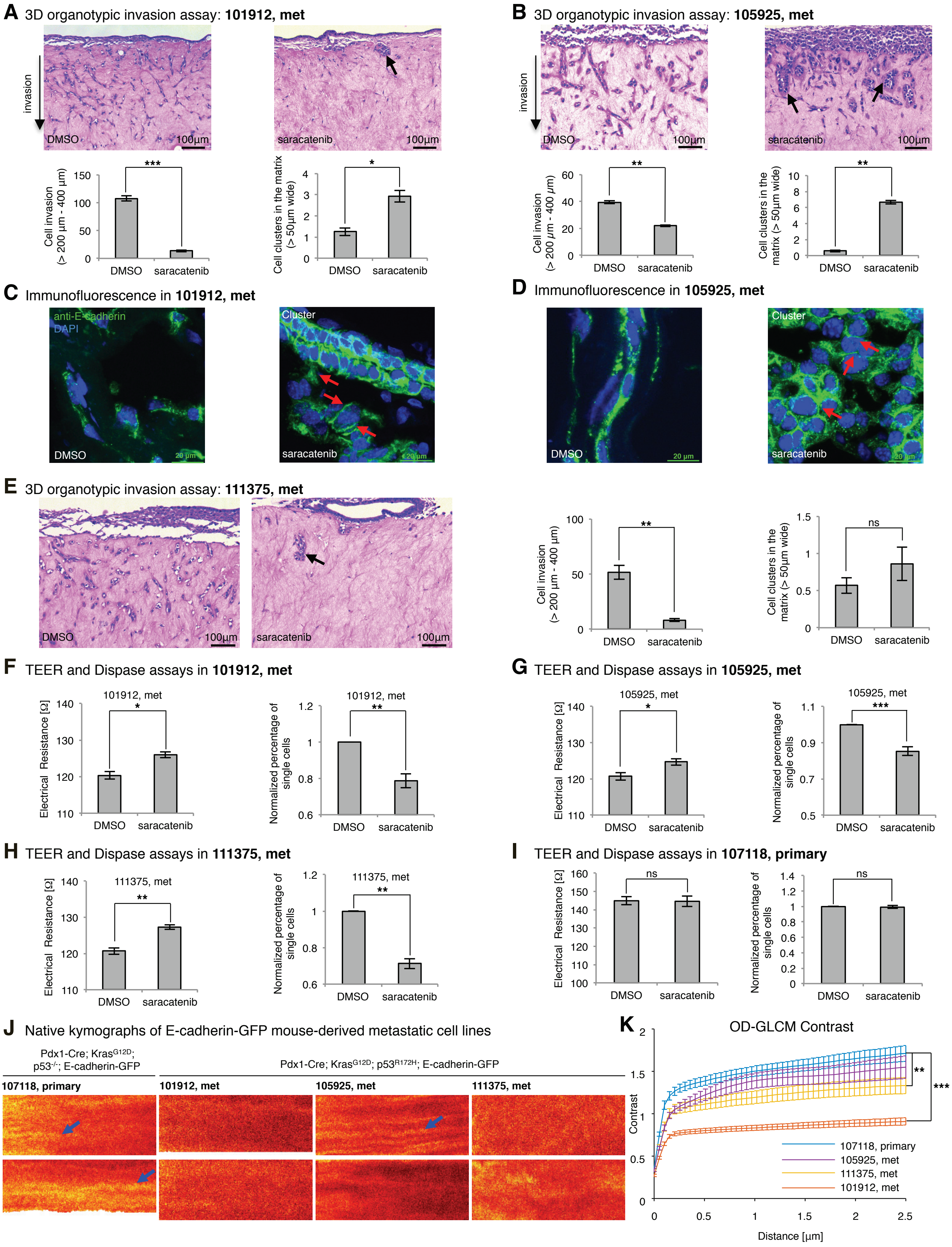
A 3D organotypic invasion assay: 101912, met
invasion
DMSO
100µm
saracatenib
100µm
Cell invasion (> 200 µm - 400 µm)
***
Cell clusters in the matrix (> 50µm wide)
*
DMSO
saracatenib
B 3D organotypic invasion assay: 105925, met
**
C Immunofluorescence in 101912, met
anti-E-cadherin
DAPI
Cluster
D Immunofluorescence in 105925, met
E 3D organotypic invasion assay: 111375, met
ns
F TEER and Dispase assays in 101912, met
101912, met
Electrical Resistance [Ω]
Normalized percentage of single cells
G TEER and Dispase assays in 105925, met
105925, met
H TEER and Dispase assays in 111375, met
111375, met
I TEER and Dispase assays in 107118, primary
J Native kymographs of E-cadherin-GFP mouse-derived metastatic cell lines
Pdx1-Cre; Kras^G12D; p53^-/-; E-cadherin-GFP
Pdx1-Cre; Kras^G12D; p53^R172H; E-cadherin-GFP
107118, primary
101912, met
105925, met
111375, met
K OD-GLCM Contrast
Contrast
Distance [µm]
107118, primary
105925, met
111375, met
101912, met

Figure S6, related to Figure 7. Saracatenib-induced cluster formation of metastatic cells derived from the E-cadherin-GFP mouse in 3D organotypic matrices and OD-GLCM junction analysis of cells derived from the E-cadherin-GFP mouse.

(A,B) Isolated metastatic cell lines 101912 met (A) and 105925 met (B) from the liver of the transgenic mouse invading in 3D-organotypic matrices ± saracatenib (13 days, 1µM). Black arrows indicate cluster formation upon saracatenib treatment. Quantification of cell invasion between 200 µm - 400 µm and cell clustering within matrices ± saracatenib (13 days, 1µM). (C,D) Immunofluorescence staining of E-cadherin within matrices ± saracatenib (13 days, 1µM) for metastatic lines 101912 met (C) and 105925 met (D). Red arrows label cell-cell junctions within cell clusters upon saracatenib treatment. E-cadherin, green; DAPI, blue. (E) Isolated secondary metastatic cell line 111375 met invading into 3D-organotypic matrices ± saracatenib (13 days, 1µM). Black arrows indicate cluster formation upon saracatenib treatment. Quantification of cell invasion as number of cells invaded beyond > 200 µm and quantification of cell clustering within matrices ± saracatenib (13 days, 1µM). (F-I) Trans-Endothelial Electrical Resistance (TEER) measurements (left) and Dispase-based mechanical disruption (right) in cell lines derived from the E-cadherin-GFP mouse, 101912 met ± saracatenib (F) and 105925 met (G), 111375 ± saracatenib (H) and 107118 ± saracatenib (I). (J) Representative kymographs of native, unbleached junctions for 107118 primary, 101912 met, 105925 met and 111375 met. Blue arrows indicate persistent regions of high intensity. Changes in the size of the kymograph represent slight changes in length of the junction over the imaging period. (K) OD-GLCM contrast as a function of distance for 107118 primary (blue, n=36), 101912 met (red, n=30), 105925 met (yellow, n=20) and 111375 met (purple, n=22). Statistical significance for OD-GLCM was calculated based on the average contrast. Columns, mean; bars, ± SE; n = 3 per group; *, $P < 0.05$; **, $P < 0.01$; ***, $P < 0.001$ by unpaired Student's t-test.

Supplemental Movie Legends

Movie S1, related to Figure 1. Tissue-specific and induced expression of E-cadherin-GFP.

First panel, Z-stack (left) and maximum-intensity 3D movie (right) of E-cadherin-GFP cell-cell junctions in the pancreas (Pdx1-Cre; E-Cadherin-GFP), E-cadherin-GFP in green, collagen-derived SHG-signal in purple.

Second panel, Z-stack through the liver of an untreated (left) and treated (right, 3 doses of 2mg ß-naphthoflavone over 8 hours) Cyp1a1-Cre; E-Cadherin-GFP control mouse (Cyp1a1-Cre; E-Cadherin-GFP), E-cadherin-GFP in green, collagen-derived SHG-signal in purple.

Third panel, Z-stack of mammary gland alveoli (left) and mammary gland lumen (right; MMTV-Cre; E-Cadherin-GFP), E-cadherin-GFP in green, collagen I-derived SHG-signal in purple. Note elastin autofluorescence in striated green lines.

Fourth panel, Z-stack movie (left) and FRAP time lapse movie (right) of E-cadherin-GFP in isolated pancreatic islets (RIP-Cre; E-Cadherin-GFP), E-cadherin-GFP in green, collagen I-derived SHG-signal in purple. Red arrow indicates bleached region.

Movie S2, related to Figure 1. Constitutive expression of E-cadherin-GFP.

First panel, Z-stack movie (left) and FRAP time-lapse movie (right) of E-cadherin-GFP in the kidney (E-cadherin-GFP 'ON' mouse), E-cadherin-GFP in green, collagen I-derived SHG-signal in purple. Red arrow indicates bleached region.

Second panel, Z-stack movie (left) and FRAP time-lapse movie (right) of E-cadherin-GFP in the choroid epithelium of the brain (E-cadherin-GFP 'ON' mouse), E-cadherin-GFP in green, collagen I-derived SHG-signal in purple. Red arrow indicates bleached region.

Third panel, Z-stack movie (left) and FRAP time-lapse movie (right) of E-cadherin-GFP in the salivary gland (E-cadherin-GFP 'ON' mouse), E-cadherin-GFP in green, collagen I-derived SHG-signal in purple. Red arrow indicates bleached region.

Fourth panel, Z-stack movie (left) and FRAP time-lapse movie (right) of E-cadherin-GFP in the pancreas (E-cadherin-GFP 'ON' mouse), E-cadherin-GFP in green, collagen I-derived SHG-signal in purple. Red arrow indicates bleached region.

Fifth panel, Z-stack movie (left) and FRAP time-lapse movie (right) of E-cadherin-GFP in the liver (E-cadherin-GFP 'ON' mouse), E-cadherin-GFP in green, collagen I-derived SHG-signal in purple. Red arrow indicates bleached region.

Sixth panel, Z-stack movie (left) and FRAP time-lapse movie (right) of E-cadherin-GFP in the lactating mammary gland (E-cadherin-GFP 'ON' mouse), E-cadherin-GFP in green, collagen I-derived SHG-signal in purple. Red arrow indicates bleached region.

Movie S3, related to Figure 2. FRAP on CDMs

FRAP time-lapse in E-cadherin-GFP expressing $p53^{-/-}$ vector cells (left) and E-cadherin-GFP expressing $p53^{-/-}$ R175H cells (right) on 3D-CDM, E-cadherin-GFP in green. Red arrow indicates bleached region.

Movie S4, related to Figure 2. FLIP on CDMs

FLIP time-lapse imaging (top panel) and tracking of cell-cell junctions (bottom panel) in E-cadherin-GFP expressing $p53^{-/-}$ vector cells (left panel) and E-cadherin-GFP expressing $p53^{-/-}$ R175H cells (right panel) on 3D-CDM, E-cadherin-GFP in green. White rectangle indicates repeatedly bleached region.

Movie S5, related to Figure 3. FRAP in xenografts

FRAP time-lapse in E-cadherin-GFP expressing $p53^{-/-}$ vector cells (left) and E-cadherin-GFP expressing $p53^{-/-}$ R175H cells (right) in live xenograft tumors, E-cadherin-GFP in green. Red arrow indicates bleached region.

Movie S6, related to Figure 3. FLIP on xenografts.

FLIP time-lapse imaging (top panel) and tracking of cell-cell junctions (bottom panel) in E-cadherin-GFP expressing $p53^{-/-}$ vector cells (left panel) and E-cadherin-GFP expressing $p53^{-/-}$ R175H cells (right panel) in live xenograft tumors, E-cadherin-GFP in green. White rectangle indicates repeatedly bleached region.

Movie S7, related to Figures 5,6. FRAP in genetically engineered mouse models of PDAC formation and following drug treatment.

First panel, FRAP time-lapse in Pdx1-Cre; E-Cadherin-GFP pancreas (left), Pdx1-Cre; $Kras^{G12D}$; E-Cadherin-GFP tumors (middle) and Pdx1-Cre; $Kras^{G12D}$; $p53^{-/-}$; E-Cadherin-GFP tumors (right) of age-

matched mice (~ 150 days old), E-cadherin-GFP in green. Red arrow indicates bleached region.

Second panel, FRAP time-lapse in Pdx1-Cre; $Kras^{G12D}$; $p53^{R172H}$; E-Cadherin-GFP tumors (left) and Pdx1-Cre; $Kras^{G12D}$; $p53^{R172H}$; E-Cadherin-GFP tumors treated with dasatinib for 3 days (right) of age-matched mice (~ 150 days old), E-cadherin-GFP in green. Red arrow indicates bleached region.

Supplemental Experimental Procedures

Generation of E-cadherin-GFP mice Conditional E-cadherin-GFP expressing mice were generated by targeting a lox-stop-lox transgene under the control of a CAG promoter to the Hprt locus (Figure 1A), as described previously (Bronson et al., 1996; Samuel et al., 2009; Schachtner et al., 2012). The CAG lox-stop-lox transgene was constructed by firstly replacing the SA of plasmid pBigT (Srinivas et al., 2001) with the CAG promoter from pTurbo-Cre. The PGK promoter and the EM7 promoter sequence from pL452 were subsequently inserted into pBigT.CAAG by recombineering in E. coli (Liu et al., 2003). Short 5' and 3' arms homologous to sequences within the *Hprt* targeting plasmid pSKB1 (Bronson et al., 1996) were then inserted upstream and downstream of the expression cassette to generate pHprt.CAAG.STOP. A cDNA encoding the extensively validated EGFP-linked E-cadherin fusion protein ((Lock et al., 2005; Lock and Stow, 2005), a kind gift from Jennifer Stow, Institute for Molecular Bioscience, University of Queensland, St. Lucia, Queensland, Australia) was then cloned downstream of the lox-stop-lox cassette. The transgene was then recombineered into pSKB1 (Bronson et al., 1996) to generate the final targeting vector.

The vector was linearised and electroporated into *Hprt*-deficient HM1 ES cells, cultured on a DR4 mouse embryonic fibroblast feeder layer (Magin et al., 1992; Tucker et al., 1997). Homologous recombinants were selected in medium containing HAT supplement (Sigma). Correct targeting of the vector to the *Hprt* locus on both the 5' and 3' sides was confirmed using PCR on genomic DNA prepared from HAT-resistant colonies. Following identification of correctly targeted clones, mouse lines were derived from C57BL/6J blastocysts according to standard protocols. Germline transmission was identified by coat color and correct transmission of the transgene was confimed by PCR to the GFP sequences using the primers AAGTTCATCTGCACCACCG and TCCTTGAAGAAGATGGTGCG.

Animals All animals were kept in conventional animal facilities and monitored daily. Animal experiments were performed in accordance with U.K. Home Office guidelines and the Australian code of practice for the care and use of animals for scientific purposes. All mice were genotyped by Transnetyx (Cordova, TN).

Multiphoton Imaging Imaging was conducted on an inverted Leica SP8 microscope and the excitation source used was a Ti:Sapphire femtosecond pulsed laser (Coherent Chameleon Ultra II), operating at 80 MHz and tuned to a wavelength of 890 nm. Images were acquired with a 25x NA0.95 water objective. A dichroic filter (495nm) was used to separate the SHG signal from GFP emission, which were further selected with band pass filters (460/50 and 525/50, respectively). Intensity was recorded with external RLD HyD detectors. For z-stacks, images were acquired at a resolution of 1080x1080 pixels and a z-step size of 2.52 μm.

Confocal photobleaching experiments in cell culture, cell-derived matrix and *in vivo* 2.5×10^6 confluent PDAC cells (E-cadherin-GFP-transfected cell lines) or 5×10^6 confluent metastatic cells (derived from the E-cadherin-GFP mouse) were plated onto 35 mm glass bottom dishes (MatTek) and allowed to adhere and form cell-cell junctions for 36h prior to photobleaching analysis. Dishes coated with cell-derived matrix (Serebriiskii et al., 2008) were used for photobleaching on 3D substrates. Photobleaching experiments were performed using a Leica DMI 6000 SP8 Confocal microscope or an Olympus FV1000 Confocal microscope with SIM scanner (Serrels et al., 2009). Cells were maintained at 37 °C and imaging performed at 488 nm excitation using the following settings: pixel dwell time 4 μs/px, pixel resolution 512x512. For FRAP imaging, a circular area with 1.5μm diameter (FRAP imaging) or 3μm diameter (large region FRAP) bleached to approximately 50% of the original pre-bleach intensity was used in all experimental setups (Sprague and McNally, 2005). Photobleaching was achieved using a 405 nm laser, 20 μs/pixel dwell time for one frame (at > 70 μm depth *in vivo*). Images were captured every 1.6 s for ~ 5 minutes.

For FLIP imaging, a rectangular area (0.5μm wide parallel to the junction and 4μm long perpendicular to the junction) was bleached using a 405 nm laser (20 μs/pixel dwell time for one frame) followed by 10 post-bleach images acquired with 488 nm as described above. The bleach-postbleach protocol was repeated for 50 cycles to achieve robust FLIP. Images were captured every 1.6 s for ~ 20 minutes.

For photobleaching experiments in live xenograft tumors, 1×10^6 PDAC cells suspended in 100 μL HBSS (Invitrogen) were subcutaneously injected into the rear flank of CD-1 nude mouse and allowed to grow tumors ~ 1-1.4 mm, in line with animal ethics guidelines. To permit imaging mice were anaesthetized using a combination of 1:1 hypnorm - H_2O + 1:1 hypnovel - H_2O. Following induction of anaesthesia the subcutaneous tumor was surgically exposed via skin flap procedure, as

previously described (Canel et al., 2010b; Serrels et al., 2009) and the mouse restrained on a 37 °C heated stage. Intravital FRAP and FLIP imaging was performed as described above and the mice were sacrificed after the experiment within 4 h of imaging. FRAP on pancreatic tissues and tumors was performed using similar parameters as described above upon ~ 150 days of tumor development with the appearance of swollen abdomen, cachexia or reduced mobility due to tumor burden (n = 3 independent mice, per condition, ≥ 21 cell-cell junctions in total).

FRAP data analysis Recovery curves obtained from fluorescent intensity measurements were exported for exponential curve fitting, as described previously (Canel et al., 2010a; Canel et al., 2010b; Serrels et al., 2009). Data were fit using the following exponential function: $y(t) = y_0 + a * (1 - \exp(-b * x))$. The immobile fraction (Fi) was calculated as follows using values derived from the curve fit: $Fi = 100 * \left(1 - \frac{a}{1-y_0}\right)$. The half-time of recovery was calculated using the formula $t_{1/2} = (\ln 2)/b$, where b was obtained from the exponential curve fit. We calculated the 95% confidence interval on the fitted value of the immobile fraction using the Delta method (Figure S2H-J control, blue bar; mutant, red bar (Oehlert, 1992).

Quality control and validation of FRAP quantification The following quality control criteria were applied to reject data corrupted by unacceptable experimental artefacts. To control for drift in the z-axis, the intensity in unbleached areas of the image were assessed. If the intensity in these areas fluctuated or drifted over the time course, the image was excluded from the analysis. Images that exhibited minor lateral drift were realigned using the ImageJ plugin StackReg (Thevenaz et al., 1998). Images where realignment failed, were excluded from the analysis. For each fitted curve, the quality of the fit was assessed using the sum-of-squares residual R^2. Curves exhibiting an unacceptably low signal-to-noise level, as defined by a value of R^2 below 0.9 *in vitro* and 0.7 *in vivo*, were rejected from the analysis.

The FRAP data were fitted using a one phase FRAP recovery model to estimate the E-cadherin immobile fraction, as discussed above and described previously (Canel et al., 2010a; Canel et al., 2010b; Serrels et al., 2009). If multiple processes contribute to the recovery then a multiple phase recovery model, $y(t) = y_0 + a * [1 - \sum_{i=1}^{n} c_i \exp(-b_i \cdot t)]$, may be used to quantify the recovery times of the different processes. The immobile fraction, Fi= $100 * \left(1 - \frac{a}{1-y_0}\right)$, in principle depends only on the depth of the initial bleach event, y_0, and the height of the recovery, a. If a measured FRAP curve has plateaued before the end of the measurement then the estimated immobile fraction does not depend on the number and rate of the processes in the model used to fit the decay. To validate our approach to estimating the E-cadherin immobile fraction, we a) verified that our FRAP curves had reached a plateau in the measurement period and b) compared the immobile fraction calculated using a one phase and two phase model.

a) To determine whether the FRAP curves had truly reached a plateau by the end of the recorded trace we compared the fit of two models, (i) a straight line fit with a slope equal to zero and (ii) a straight line fit with a positive gradient, to the final ten points of the FRAP curve using Akaike's information criteria (AIC) (Akaike, 1981; Motulsky and Christopoulos, 2004). These models correspond to a plateau and a continued gradient, respectively. The AIC for a model is given by

$$\mathrm{AIC} = \mathrm{N} \cdot \ln\left(\frac{\mathrm{SS}}{\mathrm{N}}\right) + 2\mathrm{K}$$

where SS is the sum-of-squares residuals of the fit, N is the number of data points and K the number of fitted parameters. The difference between the AIC of the two models, $\mathrm{AIC}_{gradient}$-$\mathrm{AIC}_{plateau}$ gives an insight into the relative probabilities of the two models given the data. In particular, the evidence ratio, gives an estimate of the ratio of probabilities of the two models (Figure S2A):

$$\text{Evidence Ratio} = \frac{\mathrm{P(plateau)}}{\mathrm{P(gradient)}} = \frac{1}{e^{-0.5(\mathrm{AIC_{plateau}} - \mathrm{AIC_{gradient}})}}$$

This analysis was applied to the *in vitro* and *in vivo* FRAP data. For each dataset, the average evidence ratio for a plateau was greater than 75, indicating that the plateau model was at least 75 times more likely than the gradient model and so providing strong evidence the data had become asymptotic and plateaued (Figure S2B-D).

b) We also compared the immobile fraction calculated by fitting the *in vitro* and *in vivo* FRAP data shown to a one phase and two phase model (Figure S2E-G). Where fitting with a two-phase model failed to converge, the datasets were excluded from the analysis. We found that fitting either a single or two-phase model to our data generated from 2D (glass), 3D (CDMs) or xenografts models made no significant difference to our analysis, indicating that use of a single exponential fit

does not bias our readout of the immobile fraction (Figure S2E-G control, blue bar; mutant, red bar) and is in line with previous publications (Canel et al., 2010a; Canel et al., 2010b; Serrels et al., 2009). We note that a two-phase analysis requires a significantly higher signal to noise ratio to reliably constrain the fit parameters (Istratov and Vyvenko, 1999) and therefore chose to use a single-phase model throughout our manuscript to determine the immobile fraction. The AIC analysis providing strong evidence that the curves have plateaued in combination with the comparison of the two models suggests that fitting a single exponential model provides an appropriate and robust estimate of the immobile fraction.

Tracking junctions during FLIP acquisition Junctions were tracked over time using an optical flow based procedure. Junctions were identified using a series of manually selected points. From these points, ten evenly spaced key points along the junction were obtained using spline interpolation (Burden and Faires, 1997). The optical flow between consecutive images in the sequence was calculated using the Horn-Schunck method (Barron et al., 1994) with the Matlab Vision toolbox. The optical flow provides an estimate of the velocity of objects in the image. The optical flow map was smoothed with a circular kernel of radius eight pixels and used to calculate the displacement of the key points along the junctions between the two frames to estimate the position of the junction in the latter frame. Ten new evenly spaced points were generated from the spline connecting the displaced points and the process was repeated for all consecutive pairs of images in the sequence. This approach allowed us to follow the intensity profile along a deforming junction for up to 800s.

FLIP data analysis Where sample drift was detected, the FLIP image sequences were aligned using rigid-body registration with the ImageJ plugin "StackReg" (Thevenaz et al., 1998). In each image five junctions were tracked; the bleached junction, two junction adjacent to the bleached region and two distant junctions in cells which were not bleached. The average intensity of the junctions was tracked with time to yield intensity time profiles for each junction. In addition, the average intensity in a 1μm region around the bleach point on the membrane and two points 3μm from the bleach point was calculated for each time point. Photobleaching due to imaging was corrected by fitting an exponential decay curve to the average of the distant junctions' time profiles and dividing each time profile by the fitted decay curve. The fraction of retained fluorescence was calculated using the ratio of the average of the final five points in the time profile to the initial intensity.

Generation of kymographs Kymographs were generated for tracked junctions. At each time point, 200 evenly spaced points along the junction were generated using spline interpolation (Burden and Faires, 1997). At each point, a ten pixel line perpendicular to and centered on the junction was integrated to estimate the intensity of the junction at that point and generate a line profile of the intensity along the junction. Where the length of the tracked junction changed, the line profiles were resampled to match the spacing in the first frame. Using this analysis we were able to visualise the loss of fluorescence across a junction with time as a single point is bleached.

Kymograph GLCM analysis To assess the level of intensity variation across a junction and quantify the uniformity or texture of the E-cadherin signal, Orientation Dependent Gray-Level Co-occurrence Matrix (OD-GLCM) analysis (Haralick et al., 1973) was applied to kymographs of native (unbleached) junctions. A GLCM quantifies the number of occurrences of different intensity values at a particular spatial offset over an image and can be used to quantify the heterogeneity of an image, as previously demonstrated (Hu et al., 2012; Huo et al., 2015; Miller et al., 2015; Nobis et al., 2013; Samuel et al., 2011). The contrast parameter, $\sum_{i,j}|i-j|^2\,p(i,j)$, provides a measure of contrast between a pixel and its neighbour over the whole kymograph and serves as an indication of the differences in intensity values along the junction; a low contrast value indicates a homogeneous intensity distribution, while a high contrast value indicates areas of high and low intensity are present (Figure S3B). The contrast parameter was calculated over a range of offsets from zero to 80 pixels along the junction.

Source code for the Kymograph generation and FLIP analysis used in the manuscript is available to download from https://github.com/timpsonlab/

Large area FRAP analysis Kymographs of large region FRAP movies were generated using the approach described above. To generate an average kymograph, the kymographs were aligned based on the location of the bleach region and resampled on a uniform grid of 200 points spaced over 10μm around the bleach region. The initial intensity of the junction was corrected for by normalising each

spatial point on the kymograph to its corresponding pre-bleach intensity. Photobleaching due to imaging was corrected by fitting an exponential decay curve to the average intensity of the movie outside of the bleach region and normalising the kymographs to the fitted curve. Selected spatial profiles from the average, normalised kymograph were fitted to a Gaussian profile $I(r) = 1 - A \cdot \exp\left(-\frac{(r-r_0)}{\sigma^2}\right)$ using the Matlab curve fitting toolbox to find the width σ and depth A of the bleached region at that point.

Analysis of large area FRAP kymographs The change in the intensity profile along the membrane of a FRAP bleach region over time can provide an insight into the processes governing the recovery. To quantify this process, we used a model of FRAP recovery considering the effects of lateral diffusion in the membrane and exchange with the cytoplasm (de Beco et al., 2012; de Beco et al., 2009; Goehring et al., 2010) and relate this model to the parameters of the Gaussian fit of the measured spatial profiles. The rate equation which governs the recovery of a photobleaching event in the membrane $c^*(r,t)$, can be written in terms of the effective diffusion coefficient D_{eff} , and the rate constant of endocytosis from the cytoplasm to the membrane, k_{endo}, as

$$\frac{dc^*}{dt} = D_{eff}\nabla^2 c^* - k_{endo}c^*$$

assuming a reaction limited exchange process where diffusion in the cytoplasm is fast compared the rate of endo- and exocytosis (Coscoy et al., 2002). If the initial bleach event has a Gaussian profile with width a, it may be shown by substitution that the bleach profile will evolve according to

$$c^*(x,t) = \frac{\exp(-k_{endo}t)}{\sqrt{a^2 + 4D_{eff}t}} \exp\left(-\frac{r^2}{a^2 + 4D_{eff}t}\right),$$

a Gaussian with width characterised by $\sigma^2 = a^2 + 4D_{eff}t$ whose height decreases over time due to both diffusion and transport. If the recovery is dominated by cytoplasmic exchange, the width of the bleach region will remain uniform during the recovery as illustrated in Figure S4B. If diffusion is present, the width of the bleach region will increase over time. Simulations of recovery via diffusion and cytoplasmic-exchange are shown as line profiles and kymographs in Figure S4A,B. To estimate the diffusion coefficient, we fitted the spatial bleach profiles over time to the equation above. The diffusion coefficient was estimated from a straight line fit of σ^2 against time. Using this approach we were able to monitor and gain an insight into the contributions of lateral motion and cytoplasmic transport to the FRAP recovery process.

Drug treatment *in vitro* and *in vivo* Dasatinib (Bristol-Myers Squibb, Princeton, NJ) was administered daily by oral gavage in 80 mM citrate buffer (10 mg/kg) (or 200 nM in cell culture medium *in vitro*). Saracatenib (Assay Matrix) was used at a concentration of 2μM in cell culture medium *in vitro.* After pancreatic tumor burden was observed in mice (monitored for swollen abdomen, cachexia, reduced mobility with tumor evident by palpation), the respective animals were then administered dasatinib for three days, as previously described (Morton et al., 2010; Nobis et al., 2013). After the final treatments the tumors were subsequently imaged and FRAP recovery curves obtained, n = 3 mice per imaging group (Morton et al., 2010; Nobis et al., 2013).

Whole body OV100 imaging and cell culture Primary tumor cells from the pancreas of non-invasive Pdx1-Cre; $Kras^{G12D}$; $p53^{-/-}$; E-cadherin-GFP and invasive Pdx1-Cre; $Kras^{G12D}$; $p53^{R172H}$; E-cadherin-GFP mice or secondary metastatic tumor cells which had colonized in the liver were identified using whole body OV100 fluorescent imaging (Olympus) and surgically removed prior to disaggregation *in vitro* and outgrowth in growth media (Dulbecco's modified Eagle medium containing 10% fetal bovine serum and 2 mmol/L L-glutamine) to generate cell lines 107118 primary, 101192 primary, 101912 met, 105925 primary, 105925 met and 111375 met, as previously achieved (Morton et al., 2010). Similarly, $p53^{R172H}$ and $p53^{-/-}$ PDAC cell lines were derived from tumors harvested from Pdx1-Cre; $Kras^{G12D}$; $p53^{R172H}$ mice and Pdx1-Cre; $Kras^{G12D}$; $p53^{flox/+}$ mice (which have lost wildtype p53 expression and are referred to as $p53^{-/-}$ cells throughout the manuscript), respectively (Morton et al., 2010). $p53^{-/-}$ R175H and $p53^{-/-}$ vector cells were generated by stable transfection of $p53^{-/-}$ cells with $p53^{R175H}$ or empty vector, and selected using 0.6 mg/ml G418 (Formedium, gift from Prof Karen Vousden). Subsequently, each cell line was stably transfected with E-cadherin-GFP plasmid (Lock and Stow, 2005; Serrels et al., 2009) and selected using 100 µg/ml hygromycin. Cells were maintained in DMEM supplemented with 10 % FCS + 2 mM L-glutamine + 1 % penicillin/streptomycin solution (Invitrogen) and sub-cultured weekly at a split ratio of 1:10 (Morton et al., 2010).

Proliferation assay 1,000 cells were plated in 96-well microtiter plates (six replicates per cell line) and incubated overnight to allow attachment prior to the day 0 time point. All cell lines were cultured as described above. Cell proliferation was determined at day 3 and day 5 using the CellTiter 96 aqueous assay (Promega), in accordance with manufacturer's instructions, and normalized to day 0 after blank correction.

Cell adhesion measurement via TEER and Dispase assay TEER was measured on confluent PDAC cells seeded into 12 well transwell supports (costar) using an EVOM2 epithelial voltohmmeter with an STX2 electrode (World Precision Instruments). The results were reported as electrical resistance (Ohms) ± SEM, P < 0.05 *, P < ** 0.01, unpaired Student's t-test. For Dispase assays (Calautti et al., 1998), confluent PDAC cells were treated with 6 mg/ml Dispase II (Sigma) in PBS, and subjected to mechanical stress, as previously described (Calautti et al., 1998; Canel et al., 2010b). Single cells were counted after passing through a 40 um nylon filter (BD Falcon). The single cell number was calculated as a percentage of the total cell number achieved after treating a control well with trypsin. Primary metastatic lines were treated with 100nM dasatinib or 1µm saracatenib for 2h. Results were reported by normalizing the drug-treated samples to the corresponding vehicle-treated samples. Columns, mean ± SEM, P < 0.05 *, P < ** 0.01, unpaired Student's t-test.

3D rat tail fibrillar collagen I organotypic assay Briefly, ~ 3 x 10^5/ml primary fibroblasts were embedded in a three-dimensional matrix of rat tail collagen I. Rat tail tendon collagen solution was prepared by the extraction of tendons with 0.5 M acetic acid to a concentration of ~ 2 mg/ml. Detached, polymerized matrix (2.5 ml) in 35 mm petri dishes was allowed to contract for approximately 12 days in complete media (DMEM, supplemented with 10% FCS, Invitrogen) until the fibroblasts had contracted the matrix to ~1.5 cm diameter. Subsequently, 1 x 10^5 PDAC cells were plated on top of the matrix in complete media and allowed to grow to confluence for 4 days. The matrix was then mounted on a metal grid and raised to the air/liquid interface resulting in the matrix being fed from below with complete media that was changed every 2 days. For drug treatment, 200 nM dasatinib or 1µM saracatenib was added to the media below the matrix throughout the 13 day invasion period. After 13 days, the cultures were imaged as described, snap-frozen for cryo-sectioning or fixed using 10% formalin and processed by standard methods for hematoxylin and eosin (H&E). PDAC cell invasive index was determined by counting cells that invaded between 200 µm and 400 µm within the matrix, as previously described using 10 independent images per condition, n= 3 (Nobis et al., 2013; Reverter et al., 2014; Timpson et al., 2011). Similarly, cell clustering upon dasatinib treatment was quantified by counting cell clusters > 50 µm throughout the matrix, as previously described (Dawson et al., 2012). Representative images of 3 independent experiments are shown. Columns, mean ± SEM, *, P <0.05; **, P < 0.01, unpaired Student's t-test.

Immunoblot Cells and tissues were lysed in protein extraction buffer (50mM HEPES, 1% Triton-X-100, 0.5% sodium deoxycholate, 0.1% SDS, 0.5mM EDTA, 50mM NaF, 10mM Na_3VO_4 and protease inhibitor cocktail (Roche)). Lysates were resolved by Bis-Tris gel electrophoresis and proteins transferred onto a PVDF membrane (Immobilon-P, Millipore), blocked with 5% milk, incubated with primary antibodies against E-Cadherin (BD Biosciences, 1:1000), phospho-Y416 Src (Cell Signaling, 1:500), total Src (Cell Signaling, 1:1000), β-actin (SigmaAldrich, 1:40000) and GAPDH (Acris, 1:10000) diluted in 5% BSA overnight at 4 °C and probed with HRP-linked secondary antibodies (GE Healthcare, 1:5000) diluted in 1% milk. Signal was visualized with ECL Reagent (Pierce) on X-Ray films (Fujifilm). Cell lysates were analysed using a Bio-Plex MAGPIX Multiplex Reader (BioRad), according to manufacturer's instructions. Assays for the measurement of p-Src-Y416 (171V50039M, BioRad)) and β-Actin (171V60020M, BioRad) were used. Values obtained for p-Y416 Src were normalised to β-actin (mean +/- SEM, n=3).

Immunofluorescence For E-cadherin immunofluorescence, organotypic cryo sections were de-frosted, fixed in 4% PFA and permeabilized in ice-cold methanol. Subsequently, sections were blocked with 10% FBS and incubated with anti-E-cadherin (BD Biosciences) diluted 1:100 in 1% BSA overnight at 4°C. Sections were incubated with Alexa488-coupled anti-mouse antibody (Jackson ImmunoResearch Laboratories Inc.), counter-stained with DAPI and mounted with Vectashield (Vector). For detection of E-cadherin-GFP signal from E-cadherin-GFP mouse-derived cells, primary cell lines were grown on cover slips. Upon confluence, cells were fixed with 4% PFA for 10min and mounted with Vectashield (Vector) for direct GFP imaging via fluorescence.

Carmine and immunohistochemistry staining Mammary glands were whole mounted and fixed in

10% buffered formaline. Staining was performed using carmine alum. 4µm mammary gland sections were baked for 5 min at 80°C and placed in Xylene for de-paraffinization. Antigen retrieval was performed using target retrieval solution low-pH (S1699) 20-min water bath (α-milk, Accurate Chemical and Scientific Corp.); high-pH (S2367) 20-min water bath (α-GFP; Life Technologies). For immunohistochemistry, slides were quenched in 3% H_2O_2 prior to application of 1:12,000 α-milk, and protein block (Dako) 10 min prior application of 1:200 α-GFP. Secondary antibodies (Envision) were applied for 30 min. Visualization was achieved with diaminobenzidine (DAB+). All immunohistochemistry reagents were purchased from Dako Cytomation unless otherwise stated.

Supplemental References

Akaike, H. (1981). Likelihood of a Model and Information Criteria. J Econometrics *16*, 3-14.

Barron, J.L., Fleet, D.J., and Beauchemin, S.S. (1994). Performance of Optical-Flow Techniques. Int J Comput Vision *12*, 43-77.

Burden, R.L., and Faires, J.D. (1997). Numerical analysis, 6th edn (Pacific Grove, CA: Brooks/Cole Pub. Co.).

Coscoy, S., Waharte, F., Gautreau, A., Martin, M., Louvard, D., Mangeat, P., Arpin, M., and Amblard, F. (2002). Molecular analysis of microscopic ezrin dynamics by two-photon FRAP. Proceedings of the National Academy of Sciences of the United States of America *99*, 12813-12818.

Dawson, J.C., Timpson, P., Kalna, G., and Machesky, L.M. (2012). Mtss1 regulates epidermal growth factor signaling in head and neck squamous carcinoma cells. Oncogene *31*, 1781-1793.

de Beco, S., Gueudry, C., Amblard, F., and Coscoy, S. (2009). Endocytosis is required for E-cadherin redistribution at mature adherens junctions. Proceedings of the National Academy of Sciences of the United States of America *106*, 7010-7015.

Goehring, N.W., Chowdhury, D., Hyman, A.A., and Grill, S.W. (2010). FRAP analysis of membrane-associated proteins: lateral diffusion and membrane-cytoplasmic exchange. Biophysical journal *99*, 2443-2452.

Haralick, R.M., Shanmuga.K, and Dinstein, I. (1973). Textural Features for Image Classification. Ieee T Syst Man Cyb *Smc3*, 610-621.

Huo, C.W., Chew, G., Hill, P., Huang, D., Ingman, W., Hodson, L., Brown, K.A., Magenau, A., Allam, A.H., McGhee, E., *et al.* (2015). High mammographic density is associated with an increase in stromal collagen and immune cells within the mammary epithelium. Breast cancer research : BCR *17*, 79.

Istratov, A.A., and Vyvenko, O.F. (1999). Exponential analysis in physical phenomena. Rev Sci Instrum *70*, 1233-1257.

Liu, P., Jenkins, N.A., and Copeland, N.G. (2003). A highly efficient recombineering-based method for generating conditional knockout mutations. Genome Res *13*, 476-484.

Lock, J.G., Hammond, L.A., Houghton, F., Gleeson, P.A., and Stow, J.L. (2005). E-cadherin transport from the trans-Golgi network in tubulovesicular carriers is selectively regulated by golgin-97. Traffic *6*, 1142-1156.

Magin, T.M., McWhir, J., and Melton, D.W. (1992). A new mouse embryonic stem cell line with good germ line contribution and gene targeting frequency. Nucleic Acids Res *20*, 3795-3796.

Miller, B.W., Morton, J.P., Pinese, M., Saturno, G., Jamieson, N.B., McGhee, E., Timpson, P., Leach, J., McGarry, L., Shanks, E., *et al.* (2015). Targeting the LOX/hypoxia axis reverses many of the features that make pancreatic cancer deadly: inhibition of LOX abrogates metastasis and enhances drug efficacy. EMBO molecular medicine *7*, 1063-1076.

Motulsky, H., and Christopoulos, A. (2004). Fitting models to biological data using linear and nonlinear regression : a practical guide to curve fitting (Oxford ; New York: Oxford University Press).

Oehlert, G.W. (1992). A Note on the Delta Method. Am Stat *46*, 27-29.

Reverter, M., Rentero, C., Garcia-Melero, A., Hoque, M., Vila de Muga, S., Alvarez-Guaita, A., Conway, J.R., Wood, P., Cairns, R., Lykopoulou, L., *et al.* (2014). Cholesterol regulates Syntaxin 6 trafficking at trans-Golgi network endosomal boundaries. Cell Rep *7*, 883-897.

Samuel, M.S., Lopez, J.I., McGhee, E.J., Croft, D.R., Strachan, D., Timpson, P., Munro, J., Schroder, E., Zhou, J., Brunton, V.G., *et al.* (2011). Actomyosin-mediated cellular tension drives increased tissue stiffness and beta-catenin activation to induce epidermal hyperplasia and tumor growth. Cancer cell *19*, 776-791.

Samuel, M.S., Munro, J., Bryson, S., Forrow, S., Stevenson, D., and Olson, M.F. (2009). Tissue selective expression of conditionally-regulated ROCK by gene targeting to a defined locus. Genesis *47*, 440-446.

Schachtner, H., Li, A., Stevenson, D., Calaminus, S.D., Thomas, S.G., Watson, S.P., Sixt, M., Wedlich-Soldner, R., Strathdee, D., and Machesky, L.M. (2012). Tissue inducible Lifeact expression allows visualization of actin dynamics in vivo and ex vivo. European journal of cell biology *91*, 923-929.

Serebriiskii, I., Castello-Cros, R., Lamb, A., Golemis, E.A., and Cukierman, E. (2008). Fibroblast-derived 3D matrix differentially regulates the growth and drug-responsiveness of human cancer cells. Matrix biology : journal of the International Society for Matrix Biology *27*, 573-585.

Srinivas, S., Watanabe, T., Lin, C.S., William, C.M., Tanabe, Y., Jessell, T.M., and Costantini, F. (2001). Cre reporter strains produced by targeted insertion of EYFP and ECFP into the ROSA26 locus. BMC developmental biology *1*, 4.

Thevenaz, P., Ruttimann, U.E., and Unser, M. (1998). A pyramid approach to subpixel registration based on intensity. IEEE Trans Image Process *7*, 27-41.
Tucker, K.L., Wang, Y., Dausman, J., and Jaenisch, R. (1997). A transgenic mouse strain expressing four drug-selectable marker genes. Nucleic Acids Res *25*, 3745-3746.
